# 1st German Phage Symposium—Conference Report

**DOI:** 10.3390/v10040158

**Published:** 2018-03-29

**Authors:** Irene Huber, Katerina Potapova, Andreas Kuhn, Herbert Schmidt, Jörg Hinrichs, Christine Rohde, Wolfgang Beyer

**Affiliations:** 1 Hohenheim Research Center for Health Sciences, University of Hohenheim, 70599 Stuttgart, Germany; irene.huber@uni-hohenheim.de (I.H.); Katerina.Potapova@uni-hohenheim.de (K.P.); andikuhn@uni-hohenheim.de (A.K.); Herbert.schmidt@uni-hohenheim.de (H.S.); j.hinrichs@uni-hohenheim.de (J.H.); 2 Institute of Microbiology, University of Hohenheim, 70599 Stuttgart, Germany; 3 Institute of Food Science and Biotechnology, University of Hohenheim, 70599 Stuttgart, Germany; 4 Leibniz-Institute DSMZ—German Collection of Microorganisms and Cell Cultures, 38124 Braunschweig, Germany; Chr@dsmz.de; 5 Institute of Animal Sciences, University of Hohenheim, 70599 Stuttgart, Germany

**Keywords:** bacteriophage, phage, horizontal gene transfer, co-evolution, phage therapy, industrial phage application, antimicrobial resistance (AMR), Germany

## Abstract

In Germany, phage research and application can be traced back to the beginning of the 20th century. However, with the triumphal march of antibiotics around the world, the significance of bacteriophages faded in most countries, and respective research mainly focused on fundamental questions and niche applications. After a century, we pay tribute to the overuse of antibiotics that led to multidrug resistance and calls for new strategies to combat pathogenic microbes. Against this background, bacteriophages came into the spotlight of researchers and practitioners again resulting in a fast growing “phage community”. In October 2017, part of this community met at the 1st German Phage Symposium to share their knowledge and experiences. The participants discussed open questions and challenges related to phage therapy and the application of phages in general. This report summarizes the presentations given, highlights the main points of the round table discussion and concludes with an outlook for the different aspects of phage application.

## 1. Introduction

Phage research and related business activities (e.g., BAYER’s “Polyfagin” preparation) have some tradition in Germany [1,2]. However, with the advent of antibiotics, interest in phage applications declined and for a long time was focused on fundamental questions. Although noticeable efforts have been made to use phages as molecular tools [3,4], the application of phages in medicine [5], veterinary science [6], and for hygienic purposes along the food chain [7] only recently gained wider attention in Germany. The main reason for that is obvious: multiple drug resistant bacteria are a problem worldwide. In order to develop and implement AMR counter measures concerted actions have been initiated by researchers, stakeholders and regulatory authorities in Germany. Federal and State Ministries support these efforts with tailor-made funding programs and by joining international alliances (e.g., JPI-AMR). However, except for one major project, that is, Phage4Cure [8,9] funded by the Federal Ministry of Education and Research (BMBF), practical applications of phages in Germany are rare and still hampered by an unsatisfactory regulatory frame.

In addition, and similar to the situation in France some time ago [10], the German phage community is scattered and many did not know each other. It is in this context that all German phage researchers and stakeholders from industry, regulatory agencies, politics and society were invited to contribute to the 1st German Phage Symposium. Renowned guest speakers from all over the world enriched the conference that was to foster networking, exchange and new project ideas. As a visible result, the German Phage Forum (“Nationales Forum Phagen”, www.nf-phagen.de) was founded; its first workshop will take place on 18 June 2018 at the University of Hohenheim in Stuttgart.

## 2. Summary of Scientific Sessions

During the 3 day conference, 170 participants from 20 countries contributed to five main topics, a plenary session and small workgroup discussions.

### 2.1. Structure-Function Relationship

The session was opened with Dennis Bamford’s (University of Helsinki) perspective on the structural basis of the viral universe and functional consequences. By studying architectural principles, his group found that the variety of virus structures is restricted. Their experiments yielded two other major insights: (1) Host range cannot be used as primary criterion for the classification of viruses. Instead, all viruses on earth could be grouped into a small number of structure-based lineages. (2) Viruses may have existed before the three cellular domains of life have separated and therefore, a polyphyletic origin versus a monophyletic origin should be considered [11].

Johannes Wittmann (DSMZ—German Collection of Microorganisms and Cell Cultures) et al. approached the matter from a taxonomic point of view. First classifications of bacteriophages were based on morphology (capsid size and shape, tail existence and size) and genomic features (ss vs. ds DNA/RNA, genome size). Later, host-specificity provided another criterion for classification but could not bring convincing order into the growing number of newly discovered phages [12]. With new sequencing techniques and proteome-based tools, phage taxonomy has yet again undergone a number of changes. Wittmann and his colleagues from the Bacterial and Archaeal Viruses Subcommittee of the International Committee on Taxonomy of Viruses (ICTV) brought order into the taxonomy chaos [13] and in addition, create a tool box/method to classify higher numbers of phages simultaneously [14].

Tailed bacteriophages that infect gram-negative bacteria have developed a variety of strategies to overcome the rigid barrier of lipopolysaccharide-enforced outer membrane and to transfer their genome into the bacterial host. Pascale Boulanger (Centre National de la Recherché Scientifique) focused her talk on bacteriophage T5. After introducing its genomic composition, Boulanger pointed out how T5 architecture facilitates the sophisticated two-step infection mechanism [15,16]: After docking of T5 tail-tip proteins to the bacterial cell wall, “puncturing” occurs via interaction with *Escherichia coli* outer membrane protein FhuA and allows for limited phage DNA transfer (~8%) into the host cell. This step is accompanied by substantial destruction of bacterial DNA. The subsequent pause that occurs in vivo suggests a host factor mediated defence mechanism, before T5 completely takes over the infected cell and fully degrades the bacterial DNA by a phage-encoded, Mn^2+^-dependent DNase. How this DNase activity is regulated (while maintaining T5 phage genome integrity) is still not known. Interestingly, in vitro infection experiments demonstrated that T5 phage is able to complete DNA transfer into proteoliposomes without interruption, a finding that further strengthens the hypothesis of host-specific defence mechanisms in vivo [16].

Stefanie Barbirz et al. (University of Potsdam) investigated a similar infection mechanism with a set of O-antigen specific dsDNA model phages that infect *Salmonella enterica*. Myovirus Det7, Podovirus P22, and Siphovirus 9NA differ in tail architecture but all anchor to receptors of the bacterial outer membrane and use their enzymatically active tail spike proteins (TSP) to hydrolyse the O-antigen polysaccharide. The group speculates that tail-protruding components are non-specifically pressed against the rigid outer cell membrane and this mechanical signal then transmits to the tail to proceed with the DNA transfer. Non-O-antigen specific phages may follow a different mechanism, but similar opening steps are conceivable [17].

Although tailed bacteriophage SPP1 performs a similarly fast (within 30 min after infection) takeover of host functions, Paulo Tavares et al. from Institut de biologie intégrative de la cellule (I2BC), France showed that mechanisms and proteins involved are quite different: After injection of viral DNA, the viral helicase gp40 seems to be key for high-jacking the host replisome, that is, recruitment of the host replication machinery to the phage DNA replication foci. Moreover, a major remodelling of the host cytoplasm occurs. Most likely, this reorganization optimizes not only the efficiency of phage replication but also the assembly of virions (warehouse model) which are in close proximity to the viral replisomes [18,19,20].

In contrast to lytic phages, filamentous phages are assembled in the inner membrane of their host and secreted across the bacterial envelope without killing its bacterial host [21]. Despite their minimalistic, plasmid-like genome, filamentous phage M13 has quite a complex life cycle. There are only 11 genes and respective proteins allow for infection of host bacteria (*E. coli*), reproduction, and assembly of new phages. Sebastian Leptihn et al. (University of Hohenheim) presented a model for the assembly of filamentous phages, detailing the molecular assembly motor (one gene, two open reading frames) and speculated on how DNA translocation and protein assembly are powered [21].

### 2.2. Host-Phage Interaction & Evolution of Microbial Communities

Bacteriophages regulate and drive the evolution of microbial communities in all the various ecosystems on earth. Part of this interaction is the steady evolvement of defence and anti-defence mechanisms of the host-phage system. Stan Brouns’ (Delft University of Technology) keynote summarized the current knowledge on the diverse types and mechanisms of the Clustered Regularly Interspaced Short Palindromic Repeats (CRISPR) immune system and its application in genome engineering. For the latter, it is essential to know how upon selective pressure phages can circumvent bacterial defence systems. Several mechanisms have already been described, that is, the production of anti-CRISPR proteins [22,23,24], mutations in defence-specific target DNA sequences (https://www.ncbi.nlm.nih.gov/pubmed/10223975) [25,26], deletion of CRISPR target sites [27], genome recombination [28], and DNA glucosylation of target DNA binding sites [29,30]. The degree of CRISPR inhibition by sequence alterations or biochemical modification of DNA is strongly position-dependent. Brouns et al. propose that glucosylation-induced hydrogen bonds between the side groups of the glucosyl moieties and neighbouring bases may alter the topology of base pairs [31] which could impair R-loop formation and thereby prevent cleavage of the modified targets. However, bacteria can rapidly adapt their CRISPR defence systems by ‘priming.’ For this, Cas1–Cas2 protein complexes catalyse the addition of new spacers to the CRISPR memory bank, which in turn enables the bacterial host to recognize viral invaders even with changed sequences [32].

Interestingly, not all viral anti-defence strategies are similarly effective in fighting the different CRISPR-Cas types, implying that they have evolved independently [30,32]. The selective pressure posed upon bacterial defence by anti-defence mechanisms of bacteriophages leads to rapid co-evolution between host and phage [30,33]. Brouns also reflected on the consequences of these findings for genetic engineering, raised open questions and speculated on new applications.

Horizontal gene transfer (HGT) is an important driving force of evolution. It often involves temperate bacteriophages, which dependent on the surrounding conditions can choose between a lytic and lysogenic replication cycle. Once decided on the lysogenic state, most temperate phages [34,35] integrate their genomes as prophage into the bacterial chromosome and replicate vertically with their host. The acquisition of foreign DNA via prophages allows for selective advantages in the host cell, but also bears an additional risk of cell death if toxic phage genes are transferred to the host. Hence, the integration of prophage elements into the genome and into host regulatory circuits requires stringent regulation. Christiane Wolz et al. (University of Tübingen) presented work on such regulatory mechanisms and their consequences for *Staphylococcus aureus* and its host. *S. aureus* possesses a set of virulence factors necessary for infection of its human hosts. Some of these factors are encoded by temperate phages. Sa2 phages, for example, carry genes for Panton-Valentine leukocidin (PVL), that is, one of the β-pore-forming toxins. PVL-enriched *S. aureus* perforate infected human cells and cause necrotic lesions that are very hard to treat. Similarly, Sa3 phages carrying several additional virulence factors invade *S. aureus* by integrating into the staphylococcal *hlb*-gene leading to the loss of β-haemolysin production. The excision of the phage restores the *hlb*-gene and increases the virulence of *S. aureus*. Sa phages also become highly mobile during chronic lung infections in cystic fibrosis patients but little is known about the triggers and molecular mechanisms involved [36]. In order to gain more insight into the role and interference of mobile genetic elements in *S. aureus*, the group of Wolz established molecular tools to analyse the molecular basis for strain specific phage transfer [37]. They could show that the genetic background of bacterial hosts has a substantial impact on lysogenization, induction, and phage gene expression.

In line with this notion are the studies presented by Julia Frunzke et al. (Helmholtz Research Center Jülich), who unravelled the role of xenogeneic silencing of prophages CGP1-3 in the Gram-positive soil bacterium *Corynebacterium glutamicum,* which is important for the industrial production of amino acids [38]. Her group could demonstrate that the small nucleoid-associated protein CgpS is key to the repression of AT-rich genes in horizontally acquired prophage sequences by forming protein-DNA complexes. Overexpressing the N-terminal oligomerization domain of CgpS disrupts the integrity of these protein-DNA complexes, as truncated CgpS proteins outnumber and compete with their native counterparts but cannot bind to their DNA targets anymore. Consequently, such counter silencing leads to the activation of CGP3 prophage sequences by allowing transcription factors to bind to the respective target sequences instead, resulting in bacterial growth defects and a highly increased frequency of CGP3-induced cell death [39,40]. Beyond this role in the control of gene expression, recent preliminary data of Frunzke’s team suggest an even broader function for CgpS in chromosomal organization and replication. Bioinformatics revealed CgpS homologs in almost all actinobacterial species and, remarkably, also in the genomes of several actinobacteriophages and prophages. Despite low sequence conservation, the highly conserved secondary structure suggests an ancient function of these proteins in (pro-) phage-host interaction.

Another example of phage-mediated virulence, tripartite species interaction and phage/bacteria co-evolution was presented by Heiko Liesegang et al. (University of Göttingen). To study how bacterial resistance on phages impacts the bacterial virulence on a eukaryotic host, the group used a tripartite model system consisting of *Vibrio alginolyticus*, its phages and its host, that is, *Sygnatus typhle* (pipefish). When pipefish were challenged with three *Vibrio* strains (highly susceptible (hs), intermediate susceptible (is) and resistant (r) to phages), bacterial counts on the fish did not differ, but the gene expression of the fish clearly showed strain specific patterns. By challenging a culture of hs strains with phage solutions under co-evolutionary conditions, resistant mutants immediately emerged. Bacteria with a phage-susceptible phenotype show an increased virulence on their host fish while under appropriate conditions, phage-resistant and less virulent parasites evolve [41]. Hyperparasitism is thus an important factor for the virulence of bacterial pathogens. Comparative genome analysis shall reveal the genotypes under selection that are responsible for the acquired phage resistance as well as for the modified virulence on the eukaryotic host. In the second part of his talk, Liesegang took a closer look on *Inoviridae* genomics to identify prophages, determine their sequence composition and gene content. By this, they defined a scoring weight matrix consisting of 9 prominent features to identify new *Inoviridae*. The model for such scans and pilot data will be shared by this group on a website and further developed into a General Markov Model for (pro)-phage genomics [42,43].

The focus of Li Deng, Emmy Noether group leader at the Helmholtz Research Center München, is on the ecological role of viruses in the environment. Starting with impressive numbers of microorganisms decomposed by lytic phages, she provided a colourful picture how phages drive biogeochemical cycles, control microbial populations and maintain homeostasis. Employing viral tagging [44], novel purification procedures for environmental probes, microcosm experiments, metagenomics and bioinformatics, the group could assess, quantify and describe the ecological role of phages in the ocean, freshwater environments, groundwater aquifers, and other habitats [45]. When comparing contaminated versus unspoiled sites, an increased abundance of phages as compared to the number of their bacterial hosts was observed. One explanation how HGT contributes to this phenomenon might be the transfer of catabolic enzymes necessary for biodegradation within or across bacterial species [46]. In a strict sense, infected tissues of patients, for example, with Chronic Obstructive Pulmonary Disease (COPD), can also be defined as “contaminated environment.” Based on analyses of a Human Virome Protein Cluster Database, preliminary data suggest that patients with COPD carry more virulence factors than healthy controls [46]. As pollution of our planet increases and leaves only a few unspoiled sites for control experiments, Deng heads towards experiments in the extra-terrestrial space soon. It will be interesting to see which experimental setups she chooses for the ISS, SpaceX and Mars missions, and it will certainly yield interesting findings and new insights.

Jacques Mahillon (Université Catholique de Louvain) and his colleagues focus on the lysogeny of Tectiviridae. This family of tail-less phages with a lipid membrane as the inner layer of their capsid has been found in less than 3% of bacterial isolates. Analysis of the Tectiviridae host range showed that no simple relationship could be established between the infection patterns of these phages and their diversity. However, data revealed that tectiviruses in the *Bacillus cereus* group clustered into two major groups: the ones infecting *Bacillus anthracis* and those isolated from other *B. cereus* group members [47]. Remarkably, tectiviral plasmid-related molecules with recombinant characteristics were also discovered by analyses of whole genome sequences. Additionally, Gillis and Mahillon demonstrated that tectiviral lysogeny had a significant influence on morphology, metabolic profile, growth kinetics, sporulation rate, biofilm formation, and swarming motility of their *Bacillus thuringiensis* host [48]. All these traits are involved in the survival and colonization of Bacillus strains in different environmental habitats. Overall, Mahillon’s findings provide evidence that Tectiviridae are more diverse than previously thought and that they also have ecological roles in the already complex life cycle of *B. thuringiensis* and its kin [48,49]. Current research of the group is directed towards (1) the identification of phage-specific receptors in *B. thuringiensis*, (2) deciphering of phage-resistant mutant bacteria in order to identify and confirm the mutations involved in the resistance to tectiviruses. Preliminary results indicate changes in the sugar metabolism of the bacterial host and a slight difference in growth kinetics and swarming motility. Isolated mutants show twisted cell-chain morphology and highly increased biofilm production [50].

Josué L. Castro-Mejía et al. (University of Copenhagen) described new findings on phage-bacterium interaction in the gut and discussed the influence on intestinal and extra-intestinal disorders. Several environmental factors have been shown to contribute to imbalances in gut microbiome (GM), including diet, drugs, antibiotics and enteric pathogens [51,52]. Less is known about the impact of the virome on the GM composition, its functionality and interactions with age-related comorbidities in older adults. As part of the Danish Counterstrike Initiative [53], the group assessed probands with and without interventions (diet, exercise) in respect to their microbiome composition (prokaryotes, eukaryotes, phages), metagenome (subset), metabolome and physiological parameter. Co-abundance correlation analysis on metagenome data from faecal preparations demonstrated a large number of phage-bacterium interactions. Strikingly, the fluctuations in bacterial abundance (as a function of phage attack) resulted in dramatic variations of the global metabolic potential. It also triggered dysbiosis and influenced host renal function. Together, the data indicate that members of the gut virome are associated with age-related co-morbidities [54].

Health-relevant aspects of Shiga toxin producing *E. coli* and their phages were highlighted by Herbert Schmidt et al. (University of Hohenheim). Enterohaemorrhagic *Escherichia coli* (EHEC) are the causative agents of haemorrhagic colitis and the haemolytic-uremic syndrome (HUS). Shiga toxins (Stx) are responsible for pathogenicity and are encoded by lambdoid prophages at distinct positions in the EHEC chromosome. Other, non-Stx-encoding lambdoid prophages are integrated in the EHEC chromosomes in varying numbers. The foodborne EHEC O157:H7 strain EDL933 harbours 7–10 lambdoid prophages, two of which encode Stx1 and Stx2, respectively. The stx integration sites are close to the anti-terminator Q in the late transcribed region. Upon induction of prophages, stx is co-transcribed with the late transcribed phage genes [55].

Earlier studies demonstrated a large open reading frame 3′ adjacent to the stx genes. This ORF codes for a homologue of the chromosomal nanS gene, a well-described esterase cleaving an acetyl residue from 5-*N*-acetyl-9-*O*-acetyl-neuraminic acid (Neu5, 9Ac2) resulting in Neu5Ac as bacterial carbon source [56]. Bioinformatics revealed varying numbers of phage-encoded nanS homologs (designated nanS-p) in different EHEC strains, some of which already could be confirmed functional esterases [57,58]. Interestingly, growth ability of *E. coli* O157:H7 strain EDL933 and its isogenic mutants is dependent on the number of *nanS-p* genes; deletion of all nanS-p alleles inhibits growth on Neu5,9Ac2 completely. On the other hand, recombinant NanS-p proteins cleave acetyl residues in native mucin from *O*-acetylated neuraminic acids and *O*-acetylated glycolylic neuraminic acids [58]. Schmidt hypothesized that the prophage-encoded *nanS-p* genes represent a mobile gene pool, which ensures effective substrate utilization and therefore growth and preservation of pathogenic EHEC in the large intestine in spite of its thick mucus layer and high turnover rate. Therefore, Stx- and non Stx-prophages of EHEC should be considered a pathogenic principle of EHEC strains causing serious diseases.

### 2.3. Clinical Applications

The keynote by Mzia Kutateladze, Director of the famous G. Eliava Institute of Bacteriophages, Microbiology and Virology, started with a historical view on the use of bacteriophages for treatment of infectious diseases. This historic picture is partially based on the immense screening effort during ISTC Project G-1467: “Preparation of a detailed review article/monograph on the practical application of bacteriophages in medicine, veterinary, environmental research, based on old documents and publications.” However, the data from old studies need to be treated with caution: Experimental setups were not as strict as today in terms of statistical significance, standardized methods, or the use of controls and placebos. This has changed and current data presented for phage treatments at the Eliava Phage Therapy Center are derived from randomized, placebo-controlled, double-blind trials. For example, urinary tract infections (e.g., *Streptococcus*, *E. coli*, *Enterococcus*, *Proteus*) [59] had a phage susceptibility of ~80% and no side effects have been noted in the patients treated [60].

Another focus of the Eliava Institute is the development and use of phages for prophylactic and therapeutic treatment of non-fermenting Gram-negative bacilli (NFGNB; for example, *Pseudomonas*, *Acinetobacter*, *Burkholderia* spp.), related infections as well as their elimination from hospital environment. In vitro screening of 467 multi-resistant *Streptococcus aureus* (MRSA) strains from the UK revealed 98.5% responsiveness to treatment with *Staphylococcus*-specific phages. Similar numbers were obtained on 54 MRSA/38 toxin-producing non-MRSA strains from Germany (99%), 56 MRSA strains from New York University (95%) and 100 MRSA strains from the Royal College of Surgeon in Ireland (97%) [61]. Analogous studies have been performed for β-lactamase producing *E. coli* and *Klebsiella* [62]. Attention is also given to especially dangerous pathogens, like *B. anthracis*, *Brucella* spp. [63], *Vibrio* spp. [64,65], or *Francisella tularensis*. Here, the goal is to find specific phages as well as to unravel their biology, ecology, stability, mechanisms of host interaction and other characteristics.

Research at the Eliava Institute is not solely focused on human phage therapy including wound care products [66], but also on animal health (e.g., phage therapy in aquaculture, against cattle mastitis), and for the purpose of environmental biocontrol (e.g., bacterial blight in cotton and rice, crown-gall disease in grape, diseases caused by *Ralstonia solanacearum*). Kutateladze concluded her talk with selected case reports for irritable bowel syndrome (IBS), cystic fibrosis, post-operational infections, chronic bacterial prostatitis, and chronic *Staphylococcus aureus* skin infections as a complication of Netherton syndrome [67].

“Can phage therapy provide an alternative to antibiotics?” was the initial question of Hans-Peter Horz’s (RWTH Aachen University) presentation but at the end he would not give a general YES or NO because while phages can support overcoming the AMR crisis, they probably will not replace antibiotics. In fact, ongoing studies in Horz’s lab showed remarkable synergism of phages and antibiotics (AB) against bacteria, which otherwise are resistant against the AB alone. When the group compared the efficiency of single phages versus phage cocktails to fight multi-drug resistant *Pseudomonas aeruginosa* strains, they observed that certain phages (e.g., SL2) were functioning equally effective as phage cocktails supplemented with additional phages (SL1–4), that is, neither additive nor multiplying effects could be detected [68,69]. The case suggests that a phage cocktail can only be as effective as its most effective single ingredient. Whether this is a general rule needs to be investigated. Horz also reflected on a number of antibacterial strategies involving phages or products encoded by them [70] and concluded with emerging perspectives on the human virome [71].

Key issues for the working and use of phages in experimental therapy were addressed by Thomas Rose et al. (Queen Astrid Military Hospital Brussels). He reported on the difficult first steps of experimental phage therapy in burn wound infections. As burn wounds are often colonized by multiple bacterial species, doubts about the efficacy of a 3-phage cocktail against *P. aeruginosa* and *S. aureus* were confirmed by a lack of clinical improvement in all 9 patients treated [72]. Moreover, general obstacles like (1) the limited number of evidence based studies according to modern standards, (2) the lack of a suitable regulatory frame [73], and (3) sufficient quantities of GMP-compliant phage products prevent rapid progress in human studies that meet ethical, regulatory and scientific standards. Despite these difficulties, a few European groups (members of the Bactériophages & Phagothérapie—Consortium (PHOSA) [74] and PneumoPhage [75]) strive for, or have already started, clinical studies with phages under special regulatory provisions (PhagoBurn [76] and Phage4Cure [9,10]). At the Queen Astrid Military Hospital, eight patients received phage therapy under the Declaration of Helsinki [77], and a clinical safety study in burn patients was conducted. Several cases were presented [78] and their treatment protocols outlined: urinary bladder infections were treated with phage solutions via a Foley catheter, decontamination of nose cavity and throat was achieved by phage spray application, or soft tissue treatment by washout and rinsing with phage fluid. As a partner of the European PHAGOBURN consortium and as a consequence of their practical experiences, the group also engages in the development of phage products [66] and supports regulatory approaches that will aid the clinical application of phages [79].

Andrzej Górski reported on the experiences, practices and results of the Phage Therapy Unit at the Institute of Immunology and Experimental Therapy (Polish Academy of Science) in Wrocław. In line with Truog’s opinion, “While the most trustworthy advances come through the performance of well-designed trials, sometimes experimental treatments based on theoretical considerations alone may lead to major breakthroughs” [80], Gorski and colleagues performed more than 280 treatments according to current administrative, legislative and ethical requirements. The outcome has been evaluated meticulously and classified into 7 categories. The 40% success rate for patients that were beyond any other treatment refers to complete pathogen eradication or sustainable clinical improvement in the patients. The remaining 60% showed either questionable outcomes, transient responses, no therapeutic effects, or even deterioration as a result of phage therapy [81]. Moreover, efficacy, side effects, resistance and immune response have been assessed dependent on the route of phage administration. Immune responses were shown to be low in patients receiving phages orally and not necessarily adversely affect therapy outcome. Remarkably, phages (and their proteins) downregulated proinflammatory cytokines in mice and reactive oxygen species [82,83]. Similar findings have been noted in patients on phage therapy [83] suggesting that phage therapy in addition to its well-known antibacterial action may also have anti-inflammatory and immunomodulatory effects which may be of use in clinical medicine [84,85,86].

The efficiency of bacteriophage therapy in children with diarrheal diseases was assessed in a clinical trial by Karaman Pagava and co-workers from Tbilisi State Medical University and JSC Biochimpharm. The double-blind randomized, placebo-controlled study included 71 hospitalized children from the age of 6 months to 6 years with moderate to severe diarrhoea. The improvement of symptoms and period of hospitalization after treatment with a polyvalent phage cocktail (SEPTAPHAGE, JSC Biochimpharm) or a placebo was compared. Results were summarized as follows: Phage therapy (1) significantly shortened the hospital stay (on average for 1.9 ± 0.6 days); (2) prevented clinical deterioration (especially in case of positive test on calprotectin) and switching to antibiotic therapy; (3) alleviated the severity of symptoms; (4) did not elicit any measurable side effects. Pagava also reflected on possible immunological reactions which might decrease the efficacy of phage therapy. Taking the results of this study and literature data together, Pagava reasoned that phage therapy for the treatment of diarrheal diseases can be beneficial for both in-patients and out-patients of children of all ages.

Experiences from Phase I and II clinical trials at the International Center for Diarrheal Diseases Research (ICDDRB) in Dhaka were presented by Harald Brüssow (Nestlé Research Center Lausanne). About 60% of the children hospitalized suffered from *E. coli* diarrhoea, as microbiologically determined at local laboratories. Although enterotoxigenic *E. coli* (ETEC) were supposed to be the etiological agent, other pathogens later were found to contribute to the clinical signs. Phage therapy included and compared two phage products: a cocktail of T4-like phages produced on *E. coli* strain K803 (a prophage-free K-12 derivative) and the commercial Microgen product Coli-Proteus, that is, a phage cocktail of 18 distinct phage types [87]. After a series of safety tests, no elevated safety risk was detected for the two phage products in comparison to a placebo [88]. The double-blinded, placebo-controlled, randomized phage therapy trial at ICDDRB included 120 patients, a third of which were either treated with the aforementioned cocktail of T4-like phages, the commercial Coli-Proteus product or a placebo. No adverse effects attributable to oral phage application were observed. Although the faecal coliphage titre was increased in treated over control children, phage therapy did not outperform standard rehydration/zinc treatment. Various factors might have contributed to the therapeutic failure of the phage trial: mixed infections with other pathogens causing the diarrhoea phenotype being most likely. Indeed, subsequent stool microbiota analyses revealed a correlation between diarrhoea and increased levels of Streptococcus [89].

Bacteriophage therapy for the treatment of lung infections was introduced by Martin Witzenrath (Charité—University Hospital Berlin). Multidrug resistant bacteria are a severe threat for patients with nosocomial pneumonia, as there are no therapeutic options. Experiments with phage-derived endolysins [90] demonstrated that inhalative [5] or intraperitoneal application of Cpl-1 [91] was efficient in treating severe pneumococcal pneumonia in mice, but increased inflammation markers IL-1b and IL-6 [5]. In conclusion, Witzenrath recommended the unequivocal identification of the pathogen prior to any endolysin treatment to ensure their efficacy. He also stressed the need for more studies that (1) confirm the therapeutic safety of lysins in humans (e.g., ContraFect Phase IIa Study endolysins against *S. aureus* bacteraemia), (2) address possible risks for the development of resistance, and (3) improve pharmacokinetic and pharmacodynamic properties of therapeutic lysin products.

Whereas *Streptococcus pneumoniae* plays a major role in community-acquired pneumonia, *Acinetobacter baumannii* is causal in many hospital-acquired pneumonia, and often multidrug resistant. Mice infected with *A. baumannii* and treated with a purified phage Acibel004 preparation intratracheally showed significantly reduced bacterial load in broncho alveolar lavage fluid and lung as well as a significantly improved clinical outcome and lung permeability. Neither cellular nor humoral adverse effects were observed [92]. Chronic lung diseases are increasing and complicated by airway infections. Patients suffering from pre-impaired lungs are often chronically infected with *P. aeruginosa*, especially patients with cystic fibrosis or ‘non-CF’ bronchiectasis. The recently started BMBF-funded project “Phage4Cure” [9,10] will realize the development of phage preparations and a clinical trial to test safety, tolerability and efficacy in healthy volunteers and patients with chronic pulmonary *P. aeruginosa* infections.

The European Project PhagoBurn [76] started in June 2013 and aims at evaluating the efficacy of phage therapy for the treatment of burn wounds infected with bacteria *Escherichia coli* and *Pseudomonas aeruginosa*. The coordinator of the consortium, Patrick Jault (Percy Military Hospital of Clamart), introduced the design of the first randomized, single-blinded, multi-centric and controlled clinical trial in human phage therapy in Europe. Initially planned for 3 years, the implementation of the project plan needed much more time because the setup and quality control of a GMP-compliant phage bioproduction chain (CLEAN CELLS, France) and its approval by the French Medicine Agency (ANSM) needed 24 instead of the scheduled 7 months. The clinical trial started when the first patient was treated in July 2015 and ended in December 2016 after phage therapy in a total of 27 patients. Although analyses and discussion of the results are still in progress, some valuable results can already be summarized: (I) The first European GMP-compliant production chain is operating and approved by 3 national regulators (FR, BE, CH); (II) Design and prerequisites of randomized multi-centric clinical trials have been developed and validated; (III) More lessons will be learned and conclusions drawn after the trial results are publicly available and hopefully will shed more light on questions regarding the reliable shelf life of phage cocktails, potential development of phage resistance in patients, immune reactions and possible precautions and interventions.

Christine Rohde (Leibniz Institute DSMZ) summarized the pros and cons of human phage therapy and set it in a broader context [93]. As “stable survivalists” phages co-evolve with or drive the evolution of their bacterial hosts and modulate natural and artificial microbial ecosystems. Their potential as antimicrobials and/or modulator of microbial communities is widely recognized and our knowledge steadily increases.

“Pro phage” criteria included: (1) host specificity avoids dysbiosis in treated environments, (2) no toxic side effects if purified, (3) self-replication/self-limitation, (4) occurrence of resistance to one phage does not cause generalized phage resistance, (5) phage-resistant bacteria are often less fit/virulent, (6) inexhaustible reserves, (7) effective regardless of MDR/ESKAPE bacteria, (8) isolated phage lysins as alternative, (9) comparatively inexpensive, thus public health costs can be reduced, (10) flexible application practices, (11) phages can replace last resort antibiotics once production, purification standards and regulatory pathway are defined, (12) availability of tailor-made phage preps could be done in realistic time. In line with the concept of personalized medicine, Rohde envisages phage therapy as a flexible concept reacting to specific settings, that is, single phage preparations or mixtures may be adapted to fit changing host spectra.

On the “contra phage” side, that is, a list of possible obstacles, Rohde addressed: (I) causative bacterial pathogens must be identified unequivocally, also in mixed infections, (II) bacterial phage resistance/immune system of probands can cause problems, (III) phages hardly reach intracellular pathogens, (IV) shelf life/stability might vary from phage to phage, and requires regular titre controls, (V) phage therapy needs physicians‘ know-how, and (VI) some human body targets might be challenging for phage application, for example, bones/joints, deep wounds.

Rohde also addressed current challenges that prevent wider use and safe application of phages: (a) phage preparations need to be highly specific for the bacterial target, fully characterized, and produced according to GMP standards; (b) protection of intellectual property rights (IPR) and infrastructures are yet to be established, because phage banks, pharmaceutical production facilities, diagnostic laboratories and hospitals conversant with phage use are rare. In a multi-stakeholder approach, model licensing pathways for phage preparations should be developed which are accepted and streamlined by regulatory authorities across national borders [94]. Such prerequisites provided, Rohde suggests that phage therapy should not only be considered in life-threatening situations but could routinely avoid antibiotic treatments.

### 2.4. Application of Phages for Veterinary Practices, in the Food and Environmental Sector

The use of phages as antimicrobials has spread to other sectors, for example, food production, environmental protection and agrobioindustry. “Despite a century of bacteriophage application in medicine and as biocontrol agent, our understanding of the molecular details of the phage infection process is still limited. Few model phages have been studied in detail, but the majority of potentially useful phage-encoded resources remain untapped in this respect.” After this introductory line, Jochen Klumpp’s (ETH Zürich) keynote highlighted how investigation of molecular and structural aspects of the phage infection process can be used and transferred to efficient biotechnological applications. Using long tail fibre proteins of *Salmonella* phage S16 for immobilization, Klumpp and his colleagues developed a rapid detection assay for *Salmonella* [95], which can significantly improve food safety. Likewise, *Listeria* phage A511 [96] was employed as a model for the large family of SPO1-related phages, which attack bacteria involved in foodborne and non-foodborne diseases. Knowledge of the structure of the distal tail apparatus and deduced information about the infection process helped to establish a specific *Listeria* assay that can be easily adapted for other SPO1-related phage applications. For instance, *Erwinia amylovora* phage Y2 has its main use as an efficient biocontrol agent against fire blight in apple and pear trees, but it is also the basis for a low-cost detection assay, which will serve as an environmental monitoring tool for *Erwinia* [97].

Effects of phages in dairy fermentation processes were addressed by Horst Neve (Max Rubner-Institute Kiel). Starter cultures can be inhibited by bacteriophages and subsequently even stop fermentation completely. *Lactococcus lactis* and *Streptococcus thermophilus*, prominent bacteria in mesophilic and thermophilic starter cultures, can be attacked by a broad range of bacteriophages. For *L. lactis* at least 10 different phage groups are known [98], and many strains do also contain prophages. In dairies, there are also hybrid *S. thermophilus* phages with genome regions derived from both, lactococcal and *Streptococcus thermophilus* phages [99], as well as phages attacking only flavour-producing *Leuconostoc* strains. Interestingly, a pilot study shows that phage types differ when comparing raw milk with whey or other dairy products [100], the reasons for which remain to be elucidated. In order to prevent economic losses and maintain process safety, Neve et al. recommend monitoring of all starter cultures and a special focus on thermo-resistant and new phage types. To this end, he and other colleagues focus on fast and reliable detection assays as well as on non-thermal removal processes (e.g., membrane filtration immobilization/UV-C irradiation [101,102]) that can significantly reduce phage load in fermentation processes and products thereof.

*Campylobacter* is an important food-borne pathogen. The family comprises 25 species of which thermophilic *C. jejuni* and *C. coli* are the most common causes of acute bacterial enteritis. In his talk, Stefan T. Hertwig (German Federal Institute for Risk Assessment) focused on the characterization of *Campylobacter* phages and their application along the food chain. Although *Campylobacter* phages have been used to fight this bacterium in chicken since 2003, not much is known about the genetics of these phages, to some extent because of unusual DNA modifications of the *Campylobacter* phage genome. According to genome size, these phages are classified: Group II (180–190 kb) and group III (130–140 kb) are the most common, while group I (320 kb) is rare. Data base searches and genome organization revealed a close relationship of phages belonging to each group. Based on the genome organization of group II phages (4 modules separated by long repeat regions), Hertwig et al. developed a multiplex PCR system [103].

While group II phages lyse strains of *C. jejuni* and *C. coli*, group III phages exclusively infect *C. jejuni*. However, group III phages generally lysed more C. *jejuni* strains than group II phages. In addition, the in vitro kinetics of cell lysis diverged in the two groups, probably caused by the different burst size of phages [104].

Sophie Kittler (University of Veterinary Medicine Hannover) and colleagues presented a practical approach of using phages to reduce *Campylobacter* load in broiler chickens. Risk assessment considers the reduction of *Campylobacter* in primary production to be most beneficial for human health [105,106]. Based on various pilot studies [6,107,108], a phage cocktail of four *Campylobacter* phages was tested during three *in vivo* trials under experimental conditions and in commercial broiler houses. Significant reduction of *Campylobacter* counts was confirmed for all trials under experimental conditions as well as in the two field studies. Reduction of up to log_10_ 3.2 CFU in *Campylobacter* load at slaughter was demonstrated in one field trial; one day after phage application *Campylobacter* numbers in another experimental group were reduced under the detection limit (<50 CFU/g) in faecal samples. Resistance analyses with re-isolates yielded three major results: (1) Phage-susceptible *Campylobacter* overgrow resistant isolates, (2) resistance of *Campylobacter* against phages stabilizes at a low level after an initial increase, and (3) different mechanisms of resistance seem to affect different phages. The latter seems quite plausible considering recent findings published by Doron et al. [109].

In light of modern food technologies, an interesting question was raised by Meike Samtlebe (University of Hohenheim) et al.: Do hygienic measures and subsequent reduced microbial load of our food (and thus also reduced number of phages) influence our intestinal performance and microbial balance? The human gut contains about 10^15^ individual phage particles, but little is known about their impact on gut microbiota [110], health and diseases. It is obvious that phages influence their bacterial hosts in various ways and hence, could specifically be employed to modulate the microbial composition of the gastrointestinal tract to maintain a healthy balance. However, targeted application of phages by integration into food matrixes faces numerous challenges, for example, bacterial resistance, manufacturing issues, suitable delivery systems and the adaptation to gastrointestinal conditions. In vitro experiments with encapsulated *Lactococcus lactis* phage P008 were carried out to test phage viability under various conditions (encapsulation techniques, enzymes/pH of surrounding fluids). In comparison to free phages (surviving pH > 2.5), encapsulated lactococcal phages are protected during their transit through the stomach and are released effectively under intestine conditions. This result could be confirmed in a dynamic in vitro gastrointestinal model (TIM-1). The study also demonstrated a protective effect of dairy matrices resulting in significant higher phage survival rates after undergoing acid gastric conditions. In conclusion, phages may be suitable modulators of human gut microbiota when applied through dairy food matrices [111].

The potential use of bacteriophages in honeybees was presented by Hannes Beims (Lower Saxony State Office for Consumer Protection and Food Safety) and targets *Paenibacillus larvae* as the causative agent of American foulbrood. To combat this most serious bacterial disease in honey bees, the team isolated and characterized *P. larvae*-specific bacteriophages from infected beehives. Whole-genome analysis of the phages allowed for a detailed safety profile and uncovered their lysogenic nature [112]. The bacteriolytic activity of phages HB10c2 and HBχ (*Siphoviridae*) was tested in plaque assays and growth inhibition was found for all genotypes of *P. larvae* tested (ERIC I–IV), as well as for 40 field isolates of the genotypes ERIC I and II. In vivo bioexposure assays showed that the feeding of bee larvae with bacteriophages has no negative effect on the development of the brood. In fact, mortality of bee larvae was reduced by phage application. Therapeutic effect could be improved by daily application.

### 2.5. Phage Lysins and Commercial Perspectives

In some cases, the use of whole phages to eliminate bacterial pathogens is inhibited either by active defence systems of the host [109] or physico-chemical conditions of the surrounding environment [113]. The use of phage-specific enzymes can circumvent such restrictions and moreover, may bypass the limited host range of most phages. Aidan Coffey (Cork Institute of Technology) presented a successful example of this approach by using phage-derived peptidoglycan hydrolases to target MRSA and antibiotic resistant *Clostridium difficile* [114]. After genomic characterization of three anti-staphylococcal phages (DW2, K, and CS1), their genes for peptidoglycan-degrading hydrolases were cloned. One of the phage endolysins displays a modular organisation with three domains: a Cys/His-dependent amido hydrolase peptidase (CHAPk), an amidase, and a cell-wall binding domain [115,116]. The latter facilitates attachment of the enzyme to the bacterial cell wall, while the other two domains catalyse the degradation of the peptidoglycan to mediate rapid bacterial cell death. Deletion analysis showed that full lytic activity against antibiotic-resistant staphylococci was retained, even when truncated to its CHAPk (peptidase) domain. X-ray crystallography and site-directed mutagenesis allowed further insight and modelling of its enzymatic mechanism. CHAPk was successfully tested in vitro on MRSA cultures and in vivo eliminated MRSA colonization in mouse without adverse effects. Ex vivo application showed no inflammatory response in primary human umbilical vascular endothelial cells (HUVECs) but immunogenicity in peripheral blood mononuclear cells (PBMCs) was detected in some subjects [115,117]. Analogous experiments were presented for the amidase endolysin from *Clostridium difficile* bacteriophage CD6356. In order to deliver the designer endolysins to their targets, a host-specific secretion and expression system was developed for dairy application (*L. lactis*) and successfully tested. Additionally, nanoparticle gels and adhesive dressings with anchored CHAPk-nanoparticles were developed for skin application. A thermal trigger concept (activation of CHAPk at 37 °C) was successfully implemented.

Wolfgang Mutter et al. from HYpharm reported on a similar approach using designer lysins to target MRSA. He stated that lysins are as efficient as antibiotics, have a comparable minimum inhibitory concentration (MIC) and work much faster. Their application on surfaces (skin, nasal mucosa) is possible, but requires optimization of the proteins with respect to stability and expression rate. Moreover, one has to keep in mind that in vivo applications can trigger immune responses in some cases, that is, production of anti-lysin antibodies in the host [118]. Based on their unique phage recombinant protein technology, HyPharm holds eight patent families.

One designer lysin HY-133 which is directed against *Staphylococcus aureus* has successfully passed laboratory and animal (cotton rat model) tests. In comparison to PRF-119, another recombinant chimeric bacteriophage endolysin [119], HY-133 displays the same activity and specificity but a significant higher stability. The molecule is currently in GMP production; clinical phase I trials are planned for 2019. The studies are conducted by an interdisciplinary public-private consortium (Fraunhofer ITEM, Coreolis Pharma, Center for Clinical Trials Tübingen, University Hospital Münster, German Center for Infection Research) and get support from German regulatory authorities (Federal Institute for Drugs and Medical Devices—BfArM and Paul-Ehrlich-Institute—PEI) and funders.

The representative of Micreos BV, Steven Hagens, pointed out that not all phages are suitable for bio-controlling. Which selection criteria need to be met was discussed on the basis of two examples, a single phage product against *Listeria* (Listex P100 = PhageGuard Listex) and a two-phage cocktail targeting *Salmonella* (S16 + FO1 = PhageGuard S). Favourable for PG Listex is (a) the extremely broad host range within the *Listeria* genus (b) the high efficacy (94–100% reduction) for various *Listeria*-contaminated food sources after storage for 6 days, and (c) its speed of action (1 min seems sufficient) [119].

Because of its different receptors, phages in PhageGuard S can attach to cell receptors present on all *Salmonella* serovars. Additional DNA modifications protect the phage product against varying bacterial defence mechanisms. An industrial trial consisting of 7 s dip treatment/24 h hold for various meat products resulted in a 1–3 log reduction of *Salmonella* counts [120]. Regulatory authorities have acknowledged safety and efficacy of both products by approving their use in the US, Canada, the Netherlands, Australia and New Zealand. Nevertheless, food manufacturers need to comply with hygienic rules; phages can neither mask poor hygiene nor replace it.

## 3. Plenary Session with Panel Discussion “Quo Vadis, German Bacteriophage Research?”

One of the conference highlights was a panel discussion with participants from several sectors. It mainly covered the phage application in human medicine but also dealt with other related fields. Representatives from two main regulatory bodies the Federal Institute for Drug and Medical Devices (BfArM) and Paul-Ehrlich-Institute (PEI, Federal Institute for Vaccines and Biomedicines), academia and industry engaged in an in-depth discussion around the current state of affairs and the most pressing problems in the field of phage application. The discussion started with the acknowledgement that there is an extensive amount of basic research taking place in Germany. A national phage bank has been set up at Leibniz Institute DSMZ-German Collection of Microorganisms and Cell Cultures in Braunschweig about 25 years ago and is further expanding [121]. Its main aim is to stock phages against the most common pathogens, for example, ESKAPE. The bank is now involved as a pool for the first ever government-funded clinical trial with Charité University Hospital in Berlin (Phage4Cure) [9,10]. Still, a range of problems has been identified during the discussion, such as:

- Lack of a clear regulatory framework;
- Lack of a clearly pre-defined phage product;
- Lack of clinical trials;
- Lack of financial incentives, particularly for start-ups and small companies;
- Lack of involvement of the pharma industry due to the low return on investment [122] as well as liability and reimbursement issues.

Thus, the situation in Germany is similar to most Western countries and lags behind the developments in some EU member states like Poland, Belgium, France or The Netherlands. The current situation in Germany has been described as one where everyone appears to be waiting for the other side to take action and break the vicious circle of a missing approved phage product. Academia pointed to the lack of a clear European regulatory framework for phages and the rather confusing responsibilities regarding the application of phages throughout the various settings like human and animal health, food, plant and environmental protection as well as hygiene.

The regulatory bodies stated that there is no clearly defined product out there prompting an immediate licensing activity. While achievements have been made in the basic research and all the needed technology is available there are still no clinical trials running except for the aforementioned “Phage4Cure” project. It was suggested that efforts should be made to develop pilot like clinical trials targeting clearly defined disease syndromes with limited numbers of patients instead of huge time- and money-consuming multi-centre studies. Medical practitioners, under the umbrella of their respective medical organizations, will not use phages as there is no approved medicinal product available and no health insurance company would cover the costs. Some patients still travel to Georgia or Poland for help and their number is expected to increase in the future. Two more points have been identified as hurdles: (a) there are no patient organisations in Germany who would advocate for phage therapy, and (b) the public awareness of the topic is still low, although the media coverage has been on the rise for the last few years.

Both regulatory bodies declared their general openness for dialogue and willingness to support companies who plan to develop a product and apply for an approval. They urged the interested companies to be more proactive and approach the authorities in advance in order to discuss the issues in detail and avoid costly procedures and prevent errors which could jeopardize the approval process. They reported on their experiences with the European Medicines Agency (EMA), which employs a SME Office, that is, a unit advising small and medium-sized enterprises [123]. Unfortunately, this service is rarely being used. A promising instrument is the so-called PRIME scheme, which allows for an accelerated approval of medicinal products where an urgent unmet medical need exists [124]. Concerted efforts to set up a European regulatory frame are desirable and could benefit from lessons learned during similar initiatives, for example, the regulation of genetically modified plants. The speakers concluded with a statement that any regulatory attempt can be a reactive one *per se*, and thus encouraged companies to be more proactive and engage in an early and intensive exchange with regulatory bodies.

Both small company representatives complained about the lack of interest from the pharma industry. Hansjörg Lehnherr (PTC Phage Technology Center GmbH) pointed out that it is not financially feasible to develop a GMP-compatible phage product for a small business and pleaded for more support from the government. Wolfgang Mutter from Hyglos GmbH reported on their successful cooperation with the authorities and with three university hospitals, all three partners of the German Center for Infection Research (DZIF). The cooperation project aims at testing of HY-133 in three German clinics, an active component against *S. aureus*, which has been designed by the company together with the University Hospital Münster [125,126]. Mutter highlighted the essential role of the DZIF in development and research of phage products, as a government-funded body comprising 35 leading research organisations in the field of infection research, with the needed finances, expertise and flexibility. Both company representatives referred to successful approval cases in the US as a potential model for Germany or the EU. However, there has also been some controversial debate on how to get approval for endolysin products.

All participants agreed that there is an urgent need to establish a dialogue between all stakeholders including regulatory bodies, legal experts, industry (pharma, biotech, food), insurance companies, researchers and academia to define possible solutions. Such a dialogue could lead to a more favourable climate for research and development of phage products throughout all sectors.

Furthermore, general funding possibilities were discussed. A representative of VDI/VDE Innovation + Technology GmbH (a project management agency for BMBF) informed that the German Federal Government was to allocate up to 500 million Euros (i.e., a two-third more than before) in the coming decade to fight antimicrobial resistance [127]. That framework which was agreed upon during the G20 Health Ministers meeting in Berlin [128] could offer the needed support for phage research and application consortia.

In conclusion, it was established that bacteriophages have become a source of hope in the face of ever-increasing AMR problems and that more coordinated efforts are needed to engage all stakeholders in a dialogue and to raise public awareness. Any phage application and regulation efforts should be in line with the WHO’s One Health approach which addresses all settings as one single system [129,130]. Pharma and health insurance companies, medical practitioners as well as patient organisations are expected to be the promotors of innovation in phage therapy. An important key role is placed with the DZIF as the only organisation in Germany today capable of introducing the desired product. Some of the issues are regulated nationally (e.g., blood products, tissue preparations, vaccines under the responsibility of the PEI) [131], however, in the long-run effective regulatory measures should best be aimed at the supra-national level [94].

## 4. Conclusions and Perspectives

The diversity of phages, their properties and functional interactions in various settings are enormous. With the increasing number of studies, our knowledge about the structure, function and interaction of bacteriophages becomes richer in detail and further substantiated. The current “Omics” repertoire and other methods also help to revisit “old” findings and put them into new context. Extra potential comes with the yet “hypothetical proteins” that could complete our picture of structural elements and unravel their functional significance and relations. Interaction of phages within their specific environments opens up exciting new fields of research and application. For instance, the role of phages in modulating the human microbiome has been addressed in several talks and lively discussions during the 1st German Phage Symposium. Since then, a number of papers have shed more light on this topic in humans [132,133,134,135], animals [136,137,138], and the environment [139] making it safe to predict that phage therapy will conquer this emerging field of application rather sooner than later.

One century of ground breaking and experimental phage research has opened new perspectives and set the stage for multifaceted applications of bacteriophages. Many methodical obstacles were removed, “teething problems” of GMP-compliant phage production have been addressed, and some regulatory hurdles have already been taken. And yet, reality does not keep pace with scientific progress. IP protection, licensing and other regulatory issues must be adapted to the new world of personalized medicine and other fields of phage application, not vice versa. Open access to data and suitable infrastructure (phage repositories, GMP-compliant production facilities, diagnostic units and clinics experienced with phage use) is needed to tap the full potential of phage applications. What’s more, close international cooperation can compensate for the still limited number of phage applications worldwide. Conferences and other exchange platforms offer a suitable forum for that. It is in this context, that we would like to thank all participants of the 1st German Phage Symposium for their contributions and we hope to continue this exchange at the 2nd German Phage Symposium in 2019 and at other occasions.

## Supplementary Materials

All abstracts of oral presentations and posters of the 1st German Phage Symposium are available online at http://www.mdpi.com/1999-4915/10/4/158/s1 as supplement (S1).
