# Supplementary material for: 1st German Phage Symposium—Conference Report
Source: Viruses. 2018 Mar 29;10(4):158. doi: 10.3390/v10040158 (PMC5923452; doi:10.3390/v10040158)
Supplement: Supplementary file 1 [file viruses-10-00158-s001.pdf]

UNIVERSITY OF HOHENHEIM
RESEARCH CENTER FOR HEALTH SCIENCES

1st German Phage Symposium

Program and Abstract Book

09 – 11 October 2017

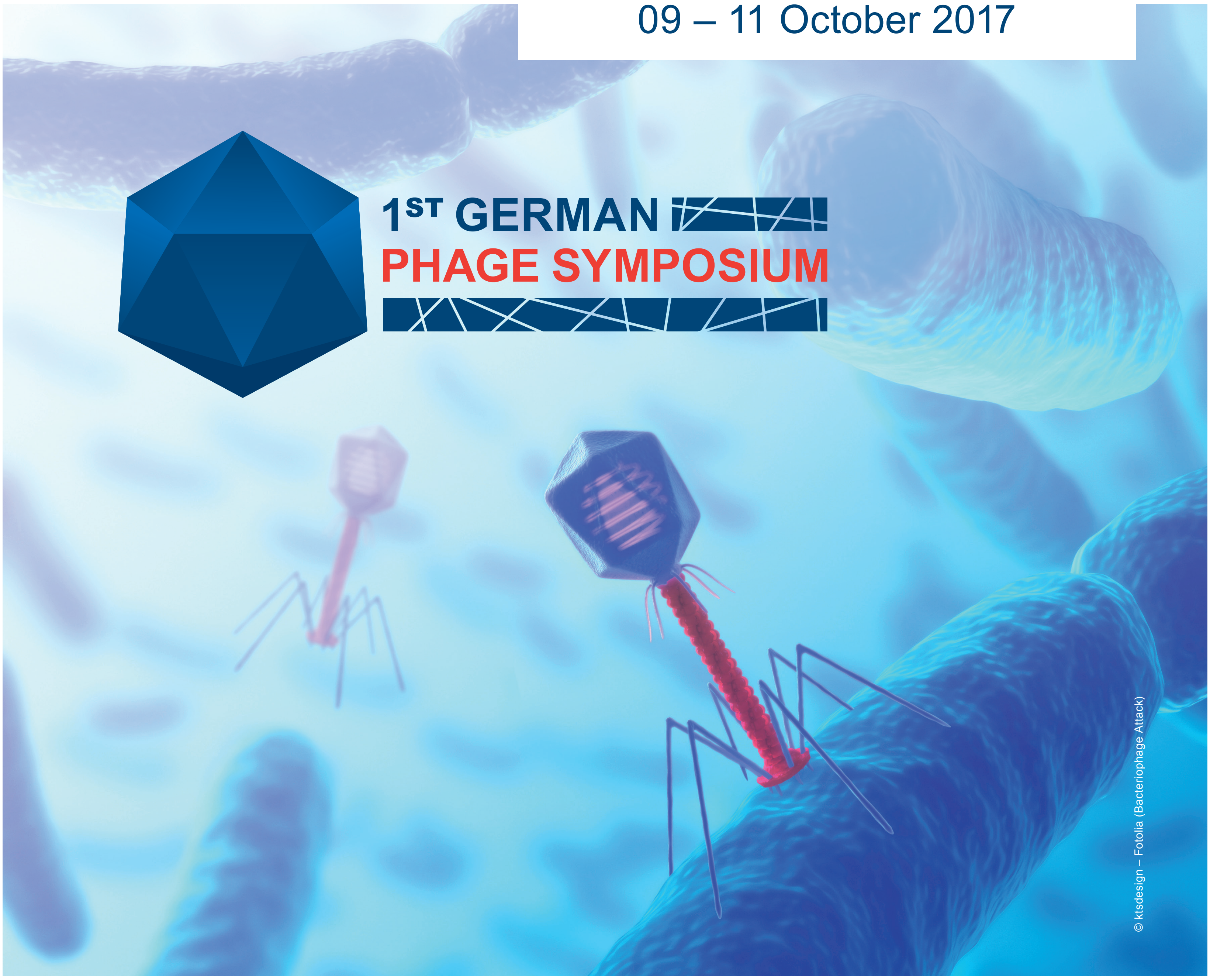

www.1st-german-phage-symposium.uni-hohenheim.de

Thanks to Our Sponsors!

DFG Deutsche Forschungsgemeinschaft

Universitätsbund Hohenheim e.V.
Freunde und Förderer der Universitöät Hohenheim

cc pharma

BASF
We create chemistry

HYpharm

Thanks to Our Supporters!

Boehringer Ingelheim Stiftung
40 years of promoting scientific excellence.

Welcome to the 1st German Phage Symposium

9–11 October 2017, University of Hohenheim, Stuttgart

Since their description as antimicrobial agents by Felix d'Herelle in 1917 bacteriophages have been in the worldwide focus of scientific, medical and biotechnological developments either as antimicrobials to combat a wide variety of human, animal and plant bacterial infections or as useful means of removing bacterial contaminants in the food chain. They have been useful tools for the detection of DNA as carrier of genetic information and in the early and recent developments of genetic engineering.

Recent gains in understanding on what is called "active lysogeny" provide a first glimpse on what we can expect, will be learned in the near future about the actual role of phages in the evolution and their contribution to the equilibrium within bacterial communities in any kind of microbiomes.

The fast rise of multi-resistant bacteria in human and veterinary medicine necessitate the urgent search for alternatives to antibiotics or, at least, supplementary approaches for treatment of bacterial infections. Phage therapy has been accepted as an effective antimicrobial procedure for many decades in Eastern Europe. Before similar acceptance is possible in the West several important scientific questions and regulatory issues need to be addressed and solved in a timely fashion.

The German Phage Symposium, held for the first time at the University of Hohenheim, aims to provide old and young researchers moving into this fascinating field of research the opportunity to meet colleagues, find new partners for future collaborations and enable to discuss their data and opinions in an open-minded atmosphere.

A special session will be held on Day 3 aiming to discuss the opportunities for further promotion of the German phage related research, so as to embed it in the already existing international networks and how to overcome current obstacles hindering a more future-oriented development in Germany. The Panel Discussion „Quo vadis, deutsche Bakteriophagenforschung?" aims to bring together different stakeholders, including companies, funding agencies and regulatory institutions and to raise the general awareness of this extremely fast evolving social topic.

The organizers are certain they have designed an appealing program within a pleasant framework. We are looking forward meeting you at the Symposium.

Wolfgang Beyer
Scientific Director of the 1st German Phage Symposium

Katerina Potapova
Interim Managing Director
Hohenheim Research Center for Health Sciences

Scientific Board Members

SCIENTIFIC DIRECTOR

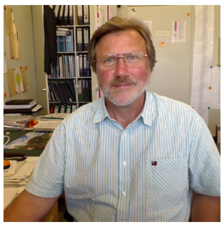

PD Dr. med. vet. habil. Wolfgang Beyer
University of Hohenheim
Institute of Animal Sciences, Department of Livestock Infectiology and Environmental Hygiene

Our research is focused on the role of temperate phages in the live cycle (prevalence, persistence, tenacity, virulence, resistence, and transfer) and of lytic phages in the control of pathogenic members of *Bacillus cereus* sensu lato., in animals and the food chain. Active lysogeny and the participation of phages in horizontal gene transfer drives the evolution of every microbial community their hosts are part of. Topics of our research include phage regulated changes in sporulation and biofilm formation as part of the organization of microbioms. Such features influence the persistence and survivability of the microorganisms in different ecosystems like soil and (waste-)water.

SCIENTIFIC BOARD

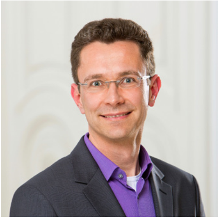

Prof. Dr. rer. nat. Martin Hasselmann
University of Hohenheim
Institute of Animal Sciences, Department of Livestock Population Genomics

Prof. Dr. Martin Hasselmann is head of the Department of Livestock Population Genomics at the Institute of Animal Science. His research interests focus on the natural variation and the evolutionary processes which provide the basics of modified gene function, evolutionary innovations and phenotypic differentiation among organisms. For more than 15 years, he is working on social insects, mainly on honey bees, integrating in the last years especially the dynamics of bee-associated pathogens and intestinal microbes.

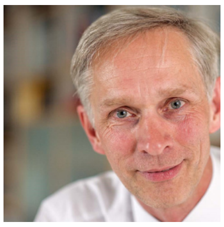

Prof. Dr.-Ing. Jörg Hinrichs
University of Hohenheim
Institute of Food Science and Biotechnology, Department of Soft Matter Science and Dairy Technology

Jörg Hinrichs leads the Department of Soft Matter Science and Dairy Technology at the University of Hohenheim since 2001. The main research topics are innovative processes and analytical tools, soft matter science of food, process and food safety. Some phages are highly thermal resistant as we found and cause fermentation problems in cheese processing being reinforced when whey products are recycled. Process safety research is addressed to minimize phage risk by thermal and non-thermal processes in food and biotechnology.

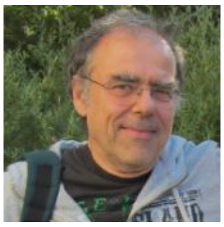

Prof. Dr. rer. nat. Andreas Kuhn
University of Hohenheim
Institute of Microbiology, Department of Microbiology

The YidC mediated membrane insertion of the major capsid proteins of bacteriophage M13 and Pf3 are the major research projects in the last years. In addition, the head assembly of bacteriophage T4, in particular the initiation complex at the membrane involving gp20 is studied. Also, the infection process and DNA ejection of T4 and T7 are studied at the molecular level.

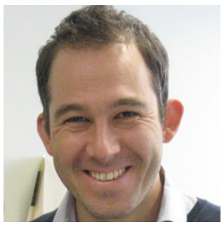

PD Dr. rer. nat. habil Sebastian Leptihn
University of Hohenheim
Institute of Microbiology, Department of Microbiology

My research aims to understand phage proteins that insert into the host membrane where they allow the translocation of molecules across the cellular envelope. We study the "ejectosome" proteins of the T7 phage which form a translocation pore, allowing the release of the viral DNA into the host's cytosol. In addition, we investigate the fascinating molecular motor of filamentous phages. This nano-machine assembles the phage in the inner membrane of the host. Our methods range from in vivo assays to single-molecule techniques.

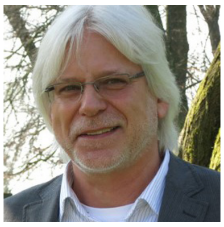

Dr. rer. nat. Horst Neve
Max Rubner-Institut
Federal Research Institute of Nutrition and Food, Kiel Department of Microbiology and Biotechnology

Our main research activities are: Microbiology and genetics of lactic acid bacteria (LAB) with emphasis on bacteriophage-host interactions, molecular identification and typing of LAB phages, gene transfer mechanisms, transmission and scanning electron microscopic analysis of dairy samples, bacteria and phages, gut phages, biocontrol phages.

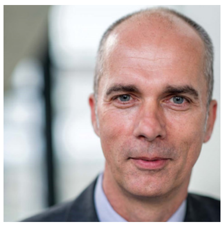

Prof. Dr. Herbert Schmidt
University of Hohenheim
Institute of Food Science and Biotechnology, Department of Food Microbiology and Hygiene

Our research focuses on lambdoid Shiga toxin-encoding and non-Shiga toxin-encoding prophages from enterohemorrhagic *E. coli* (EHEC). We are in particular interested in the function of bacteriophage genes not directly being involved in the bacteriophage replication cycle. Current research support the hypothesis that such genes may be involved in competition and maintenance of EHEC bacteria in the gut.

University of Hohenheim

The oldest University in Stuttgart: the University of Hohenheim is unique in its strong specialization.

Founded in 1818 after devastating famines, the University of Hohenheim is not only engaged in intensive basic research but has traditionally also been committed to developing innovative solutions for some of society‘s pressing problems. To do so, the University of Hohenheim engages in a combination of scientific disciplines that is unique among German universities.

Today, the University of Hohenheim is the leading University in agricultural research and food sciences, as well as strong and unparalleled in natural, social, business, economic, and communication sciences. The combination makes it possible to find solutions for many global challenges.

For more information, visit
www.uni-hohenheim.de

Hohenheim Research Center for Health Sciences

The Hohenheim Research Center for Health Sciences provides a dynamic platform for researchers, lecturers, young scientists and students dedicated to life science and societal health topics and promotes high-level research across several disciplines in accordance with the modified „One Health“ concept by

- joining expertise, e.g. in biology, immunology, health care and medicine, agriculture and food sciences, economics and social sciences
- building bridges between bench scientists, clinical investigators, health researchers, business and public stakeholders
- strengthening national and international research networks for exchange and productive partnerships
- obtaining funds for integrated research projects focusing on major scientific and societal topics, including e.g. growth, development, demographic change, lifestyle, nutrition, aging as well as their social and economic impact.

For more information about the Research Center, visit
www.health.uni-hohenheim.de

Venue

Steinbeis-Haus für Management und Technologie (SHMT)

Filderhauptstraße 142, 70599 Stuttgart, Germany

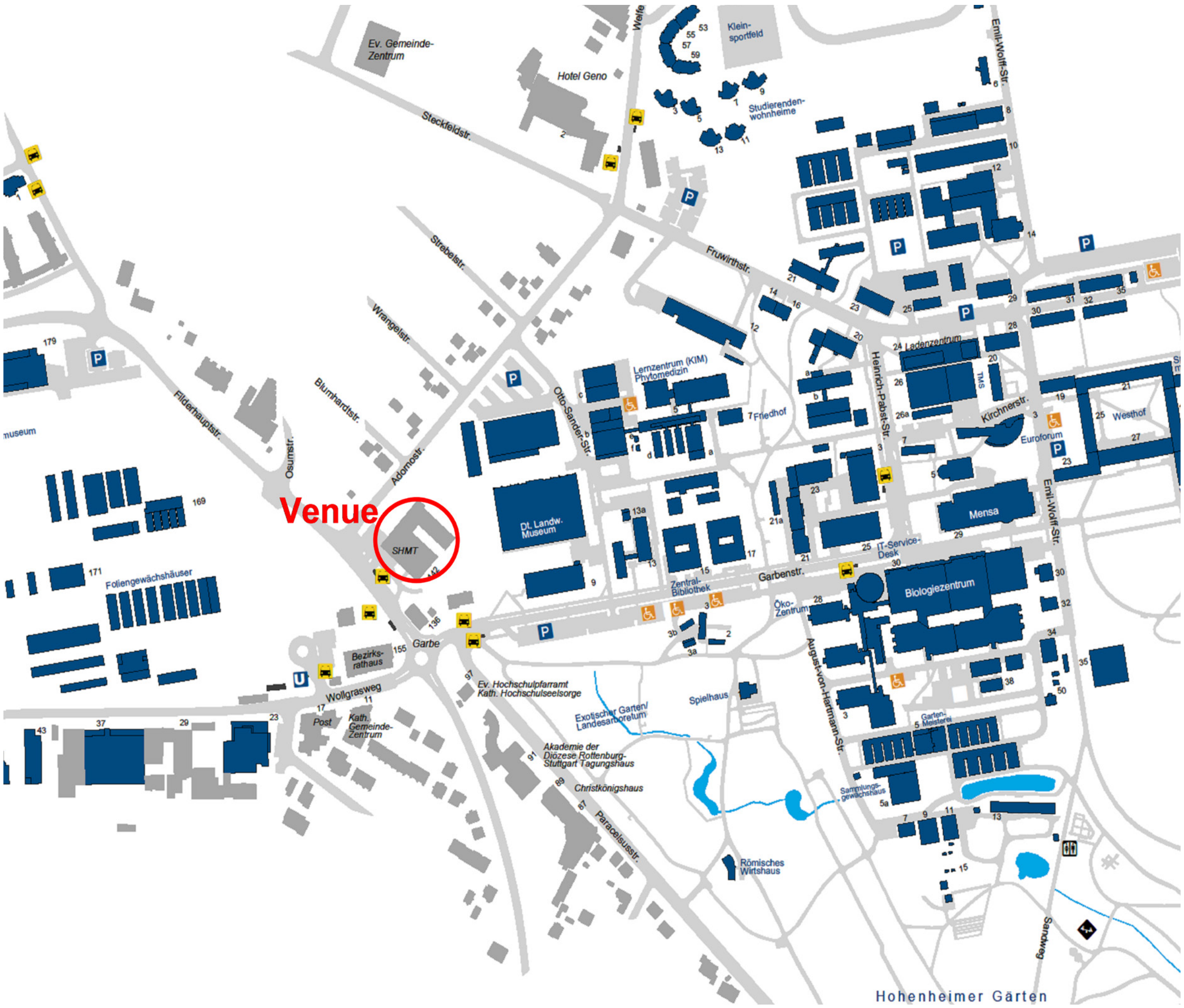

to Airport and Highway

How to reach the venue SHMT

Public transportation

From Stuttgart Airport

From the arrival terminal’s building, turn to the right to reach the bus station in approximately 3 minutes. Please follow the sign with the bus. Take bus 122 (direction „Esslingen (N) ZOB“) to the stop „Plieningen Post“. There, on the other side of the street change to one of the busses 70 or 73 until „Plieningen Garbe“. Travel time: 25 minutes.

Alternative route:

Take the S2 or S3 in Terminal 1, on Level 1 and get off at Vaihingen. There, catch the tram U3 to Plieningen. Travel time: 30 to 45 minutes.

From Stuttgart Railway Station

From the main station at Stuttgart take the tram U7 (Ostfildern) to the station „Ruhbank“. Then change to bus 70 (Plieningen) until the stop „Plieningen Garbe“. Travel time: 30 minutes.

Alternative route:

Take the tram U5 (Leinfelden Bf), U6 (Fasanenhof) or U12 (Dürrlewang) to „Möhringen Bahnhof“, and change to the tram U3 to the end station „Plieningen“. Travel time: 32 minutes.

Table of Contents

Our Sponsors 2

Welcome Address 3

Scientific Board Members 4

University of Hohenheim/Hohenheim Research Center for Health Sciences 6

Venue 7

How to reach the venue SHMT 8

Table of Contents 9

Program 10

Abstracts – Keynote Lectures 14

K1 – K4 Abstracts 16

Abstracts – Oral Presentations 20

O1 – O27 Abstracts 22

Abstracts – Posters 50

P1 – P62 Abstracts 52

Panel Discussion „Quo vadis, deutsche Bakteriophagenforschung?“ – Participants 114

List of Participants 115

Authors & Co-Authors Index 133

Notes 138

Program

MONDAY, 9 OCTOBER 2017 (DAY 1)

REGISTRATION AND WELCOME

08:30 **Registration**

10:00 **Introduction to the Symposium**
Wolfgang Beyer, Conference Chair, University of Hohenheim, Germany

10:15 **Welcome Address**
Heinz Breer, Dean of the Faculty of Natural Sciences, University of Hohenheim, Germany

SESSION 1:
Structure-Function Relationship

Chairs: Dennis H. Bamford & Andreas Kuhn

10:30 **Keynote: Structural organization of the viral universe**
Dennis H. Bamford, University of Helsinki, Finland

11:30 **Phage genomics and taxonomy – bringing order into chaos**
Johannes Wittmann, Leibniz Institute DSMZ Braunschweig, Germany

11:50 **Tectiviruses infecting members of the *Bacillus cereus* group**
Jacques Mahillon, Université catholique de Louvain, Belgium

12:10 **Singularities of bacteriophage T5 structure and infection mechanism**
Pascale Boulanger, Institut de Biologie Intégrative de la Cellule, Paris, France

12:30 **Lunch**

Chairs: Jacques Mahillon & Sebastian Leptihn

13:30 **Genome replication and assembly of the bacteriophage SPP1 particle *in vivo***
Paulo Tavares, Institut de Biologie Intégrative de la Cellule, Paris, France

13:50 **Filamentous phage assembly**
Sebastian Leptihn, University of Hohenheim, Germany

14:10 **Not a barrier but a key: How O-antigen specific bacteriophages exploit lipopolysaccharide as an essential receptor to initiate infection of Gram-negative hosts**
Stefanie Barbirz, University of Potsdam, Germany

SESSION 2:
Host-Phage Interaction & Evolution of Microbial Communities – Part I

Chairs: Stan Brouns & Herbert Schmidt

14:30 **Keynote: From understanding CRISPR biology to CRISPR 2.0 applications**
Stan Brouns, Delft University of Technology, The Netherlands

15:30 **Coffee Break**

16:00 **The role of xenogeneic silencing in (pro-)phage-host interaction**
Julia Frunzke, Forschungszentrum Jülich GmbH, Germany

16:20 **Bacteriophages of *Staphylococcus aureus* and their impact on host evolution**
Christiane Wolz, University of Tübingen, Germany

16:40 **Assess the role of viruses in contaminant biodegradation through metagenomics**
Li Deng, Helmholtz Centre München, Germany

17:00 **Bacteriophages of Shiga toxin producing *E. coli* – small molecules with high impact**
Herbert Schmidt, University of Hohenheim, Germany

SESSION 3:
Clinical Applications – Part I

Chairs: Stan Brouns & Herbert Schmidt

17:20 **Phage therapy: an alternative to antibiotics?**
Hans-Peter Horz, Uniklinik RWTH Aachen, Germany

18:00 **Poster Session & Reception**

TUESDAY, 10 OCTOBER 2017 (DAY 2)

SESSION 2 *(continued)*:
Host-Phage Interaction & Evolution of Microbial Communities – Part II

Chair: Wolfgang Beyer

09:00 **Tripartite species interaction: How phage/bacteria co-evolution impacts the virulence on an eukaryotic host**
Heiko Liesegang, University of Göttingen, Germany

09:20 **Elucidating phage-bacterium interactions that trigger changes in bacterial composition and functional profile in the gut of older adults**
Josué L. Castro-Mejía, University of Copenhagen, Denmark

SESSION 3:
Clinical Applications – Part II

Chairs: Mzia Kutateladze & Martin Witzenrath

09:40 **Keynote: Bacteriophages for treatment of infectious diseases**
Mzia Kutateladze, G. Eliava Institute of Bacteriophages, Microbiology and Virology, Tbilisi, Georgia

10:40 **Coffee Break**

11:00 **Phage therapy: From treating complications to targeting diseases**
Andrzej Górski, Bacteriophage Laboratory & Phage Therapy Center, Polish Academy of Sciences, Wroclaw, Poland

11:20 **Bacteriophage therapy in lung infections**
Martin Witzenrath, Charité Berlin, Germany

11:40 **European Project PHAGOBURN: The first randomized, single-blinded, multi-centric and controlled clinical trial in Human Phage therapy: Design and objectives**
Patrick Jault, Percy Military Hospital in Clamart, France

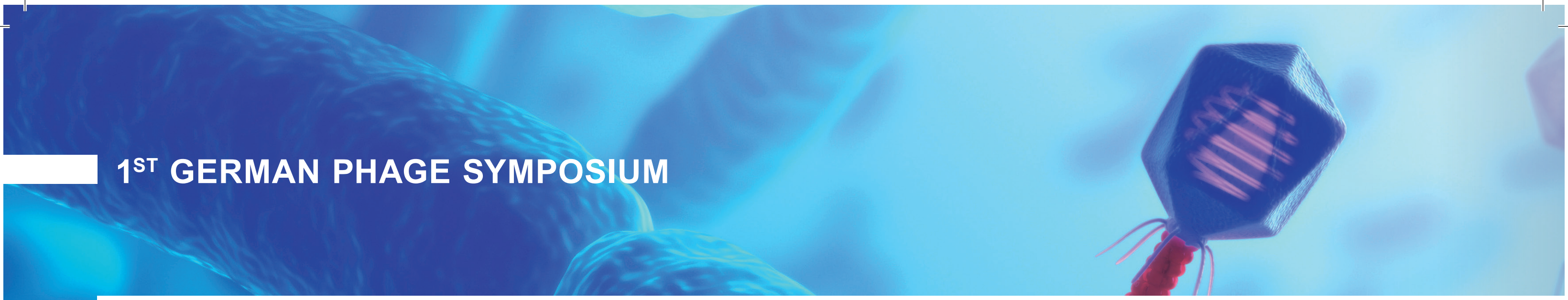

TUESDAY, 10 OCTOBER 2017 (DAY 2) *continued*

SESSION 3 *(continued)*:
Clinical Applications – Part II *continued*

12:00 **Phage therapy: History, working and use in clinical practice**
Thomas Rose, Queen Astrid Military Hospital, Att. P.H.A.G.E., Brussels, Belgium

12:20 **Efficiency of bacteriophage therapy in children with diarrheal diseases**
Karaman Pagavan, Tbilisi State Medical University, Republic of Georgia

12:40 **Lunch**

SESSION 4:
Non-Clinical Applications

Chairs: Jochen Klumpp & Martin Hasselmann

14:20 **Keynote: From genomics to practical application: Molecular biology as the basis for the use of phages in detection and control of bacteria**
Jochen Klumpp, ETH Zürich, Switzerland

15:20 **Application of lysines as an alternative to bacteriophages (Designer Lysine)**
Wolfgang Mutter, HYpharm, Bernried am Starnberger See, Germany

15:40 **Characterization of *Campylobacter* phages and their application**
Stefan T. Hertwig, Federal Institute for Risk Assessment (BfR), Berlin, Germany

16:00 **Coffee Break**

Chairs: Horst Neve & Jörg Hinrichs

16:20 **Impact of phages in dairy industry**
Horst Neve, Max Rubner-Institut, Kiel, Germany

16:40 **Reduction of *Campylobacter* load in broiler chickens by using phage application**
Sophie Kittler, University of Veterinary Medicine Hanover, Germany

17:00 **Bacteriophage application in honeybees infected with *Paenibacillus larvae***
Hannes Beims, Lower Saxony State Office for Consumer Protection and Food Safety, Institute for Apiculture Celle, Germany

17:20 **Characterization and applications of bacteriophage-derived peptidoglycan hydrolase enzymes targeting MRSA and antibiotic resistant *Clostridium difficile***
Aidan Coffey, Cork Institute of Technology, Ireland

17:40 **Carrier systems for phages to supplement food systems**
Jörg Hinrichs & Meike Samtlebe, University of Hohenheim, Germany

18:00 **Poster Session**

19:00 **Dinner** (Speakers & sponsors only)

WEDNESDAY, 11 OCTOBER 2017 (DAY 3)

SESSION 5:
Practical Applications and Regulations

Chairs: Christine Rohde & Harald Brüssow

09:00 **Phage therapy in diarrhea patients – experiences from studies in Bangladesh**
Harald Brüssow, Nestlé Lausanne, Switzerland

09:20 **Phages to combat *Listeria* and *Salmonella***
Steven Hagens, Micreos BV, Wageningen, The Netherlands

09:40 **Pros and cons of phage applications**
Christine Rohde, Leibniz Institute DSMZ, Braunschweig, Germany

10:00 **Coffee Break**

10:30 **Panel Discussion**

QUO VADIS, DEUTSCHE BAKTERIOPHAGEN-FORSCHUNG? (IN GERMAN)

Moderators: Christine Rohde & Wolfgang Beyer

› **Dr. Isabelle Bekeredjian-Ding**, Paul-Ehrlich-Institut (PEI), Langen
› **Dr. Brigitte Brake**, Federal Institute for Drugs and Medical Devices (BfArM), Bonn
› **Dr. Anne Endmann**, VDI/VDE Innovation + Technik GmbH, Berlin
› **Prof. Dr. Andreas Kuhn**, University of Hohenheim, Stuttgart
› **Dr. Hansjörg Lehnherr**, Phage Technology Center (PTC) GmbH, Bönen
› **Dr. Wolfgang Mutter**, HYpharm GmbH, Bernried am Starnberger See
› **Dr. Christine Rohde**, Leibniz Institute DSMZ, Braunschweig

SESSION 6:
11:30 **Small-Group Workshops**

13:00 **End of the Symposium**

CONTACT

Scientific Director | PD Dr. Wolfgang Beyer
Institute of Animal Science, University of Hohenheim
Phone: +49 (0)711 459 22429, wolfgang.beyer@uni-hohenheim.de

Symposium Office | Dr. Yvonne Hagenlocher, Dr. Evelyn Fein & Dipl.-Biol. Jana Tinz
Research Center for Health Sciences, University of Hohenheim
Phone: +49 (0)711 459 24688, phage@uni-hohenheim.de

For Media | Florian Klebs
Department of Media & Marketing, University of Hohenheim
Phone: +49 (0)711 459 23289, presse@uni-hohenheim.de

1ST GERMAN PHAGE SYMPOSIUM

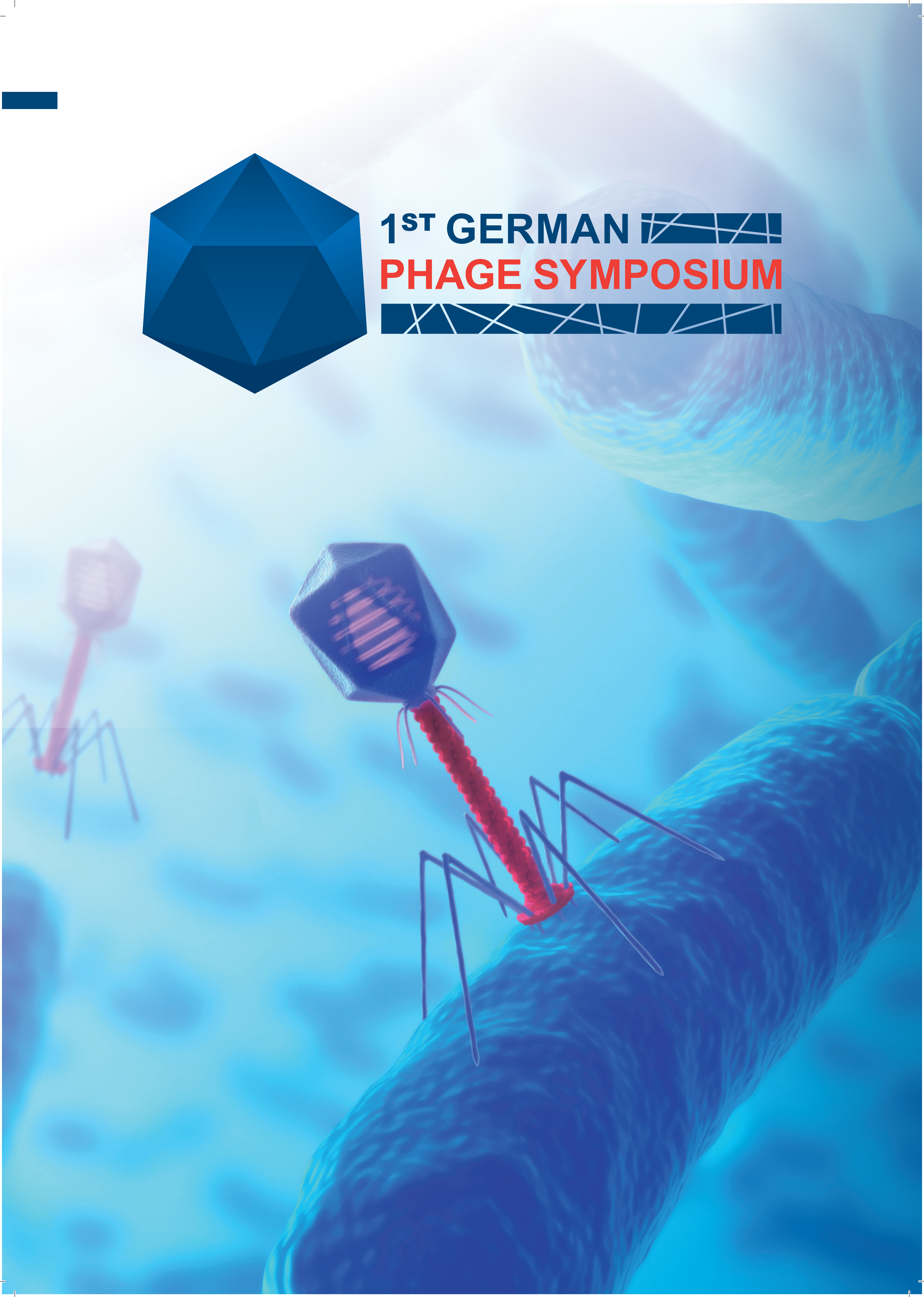

ABSTRACTS

Keynote Lectures

K1 Structural organization of the viral universe

DENNIS H. BAMFORD
University of Helsinki, Department of Biological Sciences, Finland
Corresponding author: dennis.bamford@helsinki.fi

Viruses are the most abundant living entities in the biosphere outnumbering their host organisms by one to two orders of magnitude. It is conceivable that they cause the highest selective pressure their hosts encounter. As obligate parasites viruses are dependent on their hosts but their origins seem to deviate from that of cellular life.

What are the possible structural principles to build viruses is an open question. However, structural studies on virus capsids and coat protein folds propose that there are only a limited number of ways to construct a virion. This limitation most probably is based on the limited protein fold space. Consequently, relatedness of viruses is not connected to the type of cells they infect and the same architectural principle of the capsid has been observed in viruses infecting bacteria as well as humans. Using the viral capsid architecture, it is possible to group viruses to several structural lineages that may have existed before the three cellular domains of life (bacteria, archaea and eukarya) were separated. This would mean that viruses are ancient and that early cells were already infected with many different types of viruses proposing that the origin of viruses is polyphyletic opposing to the monophyletic origin of cellular life.

We have addressed these issues by obtaining atomic level information on virion structures as well as organizing global expeditions to collect environmental samples for isolation of novel viruses. By combining these approaches, it has been possible to define structure based viral lineages giving more order to the entire virosphere. The success is certainly due to the possibility to combine structural virology to environmental one.

K2 From understanding CRISPR biology to CRISPR 2.0 applications

STAN J.J. BROUNS
Department of Bionanoscience, Kavli Institute of Nanoscience, Delft University of Technology, The Netherlands
Laboratory of Microbiology, Wageningen University, The Netherlands
Corresponding author: stanbrouns@gmail.com

The CRISPR immune system protects bacteria and archaea from invading viruses and plasmids. Immunity depends on protein complexes that use small RNA molecules to find matching viral or plasmid DNA. I will show how viruses escape immunity by mutating their DNA, and how a mechanism called priming takes care of these escaped viruses and will quickly update the memory of the immune system leading to rapid co-evolution between host and phage. All of these insights into the biology of CRISPR have led to some of the most revolutionary molecular genetics tools to date, with Cas9 being the most well known example. I will highlight some new CRISPR tools for genome engineering approaches to edit genomes and to knockdown gene expression.

K3 Bacteriophages for treatment of infectious diseases

MZIA KUTATELADZE
G. Eliava Institute of Bacteriophages, Microbiology and Virology, Tbilisi, Georgia
Corresponding author: kutateladze@pha.ge

G. Eliava Institute of Bacteriophages, Microbiology and Virology, Tbilisi, Georgia is a well-known center in the world for bacteriophage research and application. The Institute was established in 1923 by Georgian microbiologist Prof. Giorgi Eliava and French-Canadian Prof. Felix D'Herelle. Historically, the Eliava Institute was performing investigation in several directions, but phage research and applications were the main focus of its activity. Phage preparations elaborated and produced by the Eliava Institute have been successfully used in the entire Soviet Union and other Socialist countries for treatment and prophylaxis of various infectious diseases for decades.

Today, the Eliava Institute continues its activity in selection and detailed studies of phages that are active against various bacterial pathogens, including multi-drug resistant bacteria. The strains (human or animal isolates) are obtained from different geographical zones; phages (commercially available and from the Institute's collection) are being tested against the strains, and active phages are selected for further characterization. Main application of the Eliava phages is directed for treatment and prophylaxis of human bacterial diseases. Phages are successfully used to treat acute, as well chronic infections caused by antibiotic-resistant bacterial strains. Phage preparations are mainly used for treatment of urologic problems, gynecological diseases, gastrointestinal problems, skin and soft tissue diseases, respiratory system diseases, and secondary infections in cystic fibrosis patients.

The author will present several case reports after application of bacteriophages for treatment of various infectious complications.

K4 From genomics to practical application: molecular biology as the basis for the use of phages in detection and control of bacteria

JOCHEN KLUMPP[1], MATTHEW DUNNE[1], JENNA DENYES[1], ROGER MARTI[1], MARIO HUPFELD[1], RICARDO GUERRERO-FERREIRA[2], YANNICK BORN[3], LARS FIESELER[3], PETR LEIMAN[4], MARTIN J. LOESSNER[1]

[1] ETH Zurich, Institute of Food, Nutrition and Health, Switzerland
[2] EPFL, Institute of Physics of Biological Systems, Switzerland
[3] ZHAW, School of Life Sciences and Facility Management, Switzerland
[4] University of Texas at Galveston, Department of Biochemistry & Molecular Biology, USA
Corresponding author: jochen.klumpp@hest.ethz.ch

Despite a century of bacteriophage application in medicine and as biocontrol agent, our understanding of the molecular details of the phage infection process is still very sketchy. Few model phages have been studied in great detail, but the vast majority of potentially useful phage-encoded resources remain untapped in this respect.

In the current presentation, we will establish how molecular- and structural biology investigation of the phage infection process is instrumental for efficient biotechnological application of these viruses. With the 2nd and 3rd generation sequencing technologies, obtaining a complete genome sequence of a bacteriophage has never been easier. We have started from genomic data to obtain better knowledge of the structure and function of the phage adsorption and infection apparatus, and its interaction with the host cell. We will highlight how this data could be translated into practical application of phages, using *Salmonella* phage S16 and *Listeria* phage A511.

S16 is not only part of a phage preparation used in food safety applications, but its tail fibers are highly versatile and efficient detection agents for *Salmonella*. Low-cost end-point detection assays have been developed, which significantly improve food safety.

We also studied *Listeria* phage A511 as a model organism for a large family of SPO1-related phages from different foodborne and/or human pathogenic bacteria. We successfully resolved the structure of the distal tail apparatus in atomic detail, and deduced important information for fine-tuning the infection process, which can also be applied to other SPO1-related phages.

Lastly, another case illustrating the importance of molecular biology and genetics for phage application will be made based on *Erwinia amylovora* phage Y2, which was recently engineered as efficient biocontrol agent against fire blight in apple and pear trees. Y2 also serves as an efficient and low-cost detection agent for *Erwinia*.

1ST GERMAN PHAGE SYMPOSIUM

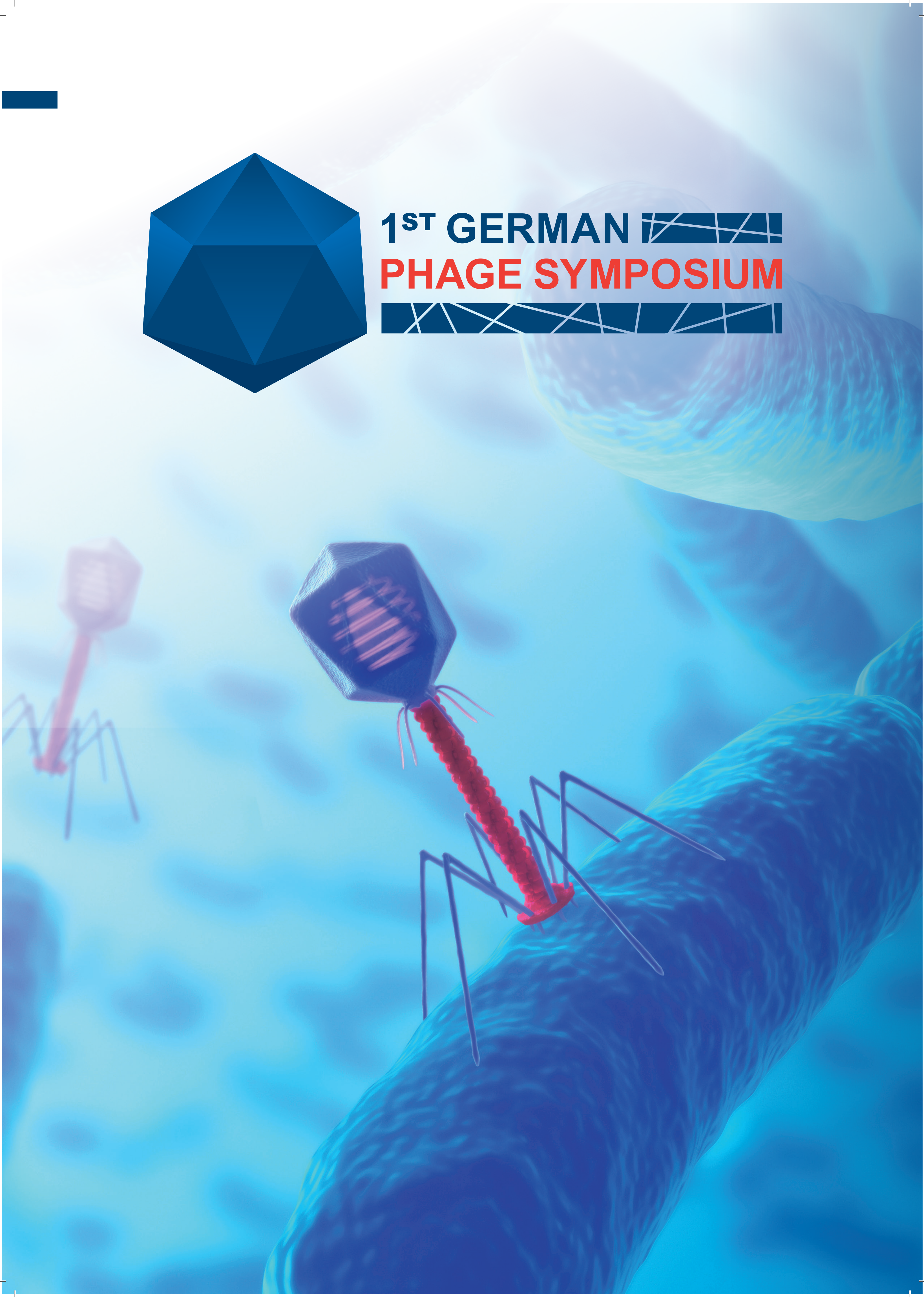

ABSTRACTS

Oral Presentations

O1 Phage genomics and taxonomy – bringing order into chaos

JOHANNES WITTMANN
Leibniz Institute DSMZ – German Collection of Microorganisms and Cell Cultures, Braunschweig, Germany
Corresponding author: jow12@dsmz.de

The Leibniz Institute DSMZ is a governmentally supported institution of the German public services and performs front-line basic and applied research. Phages for pathogenic hosts are not only our main collection focus but also in the center of our scientific interest: Phages against the ESKAPE pathogens came into our focus and intensified phage genomics being both, challenge and routine, represents the state-of-the-art methodology that can be used to shed light on the rich unknown genetic potential of the phage biodiversity. The DSMZ aims at contributing to evolution research, phage application, phage genomics and taxonomy generating further cooperative activities and contributions including those for ICTV. The Bacterial and Archaeal Viruses Subcommittee (BAVS) within the ICTV is currently holding the responsibility of classifying new prokaryotic viruses. By creating and submitting new proposals (TaxoProps), bacterial virus taxonomy is currently undergoing a number of changes since the discovery of bacteriophages in the early 20th century. First classifications were made based on morphology discovered by electron microscopy and/or nucleic acid content resulting in the three families Myoviridae, Siphoviridae, and Podoviridae in the order Caudovirales. Since new sequencing techniques improved the number of publically available bacteriophage sequences, there was urgent need for new genome and proteome-based tools using those information in order to classify phages. Benefiting from those tools, 14 subfamilies, 204 genera, and 873 species were included in the 2015 taxonomy release, since the 8th Report of ICTV.

O2 Tectiviruses infecting members of the *Bacillus cereus* group

JACQUES MAHILLON, ANNIKA GILLIS
Université Catholique de Louvain, Laboratory of Food and Environmental Microbiology, Louvain-la-Neuve, Belgium
Corresponding author: jacques.mahillon@uclouvain.be

Tectiviridae is a rare phage family of non-enveloped tail-less phages, with a double-layer capsid that contains a ~15 kb linear dsDNA located within a lipid-containing membrane. Since tectiviruses are, so far, the only phages infecting the *Bacillus cereus* group able to behave as linear plasmids during their lysogenic cycle [1]. it is important to assess the potential contribution of this type of elements to the genetic diversity of their hosts and to understand the selective pressures experienced by the bacteria when facing these phages. This work focuses on characterizing the interactions between tectiviruses and the *B. cereus* group, mainly by assessing their occurrence, genetic diversity and addressing the question of whether or not temperate tectiviruses influence their hosts' life traits. Screening of a worldwide collection of *B. cereus s.l.* strains and propagation tests indicated that tectiviruses occurred in less than 3 % of the bacterial isolates. Analysis of the tectiviruses host range showed that no simple relationship could be established between the infection patterns of these phages and their diversity. However, the data revealed that tectiviruses in the *B. cereus* group clustered into two major groups: the ones infecting *Bacillus anthracis* and those isolated from other *B. cereus* group members. Interesting tectiviral plasmid-related molecules with recombinant characteristics were also discovered by analyses of whole genome sequences. Additionally, it was found that tectiviral lysogeny had a significant influence on the bacterial growth, sporulation rate, biofilm formation, and swarming motility of their *Bacillus thuringiensis* host [2], all of which are traits involved in the survival and colonization of this bacterium in different environmental habitats. Overall, these findings provide evidence that not only tectiviruses are more diverse than previously thought, but they also have ecological roles in the already complex life cycle of *B. thuringiensis* and kin.

1. Gillis A, Mahillon J. Phages preying on *Bacillus anthracis, Bacillus cereus*, and *Bacillus thuringiensis*: past, present and future. Viruses. 2014;6:2623-72.
2. Gillis A, Mahillon J. Influence of GIL01 and GIL16 tectiviral lysogeny on bacterial growth, biofilm formation, and swarming motility of *Bacillus thuringiensis*. Appl Environ Microbiol. 2014;80:620-30.

O3 Singularities of bacteriophage T5 structure and infection mechanism

PASCALE BOULANGER
Institute for Integrated Biology of the Cell, Virology, Orsay, France
Corresponding author: pascale.boulanger@i2bc.paris-saclay.fr

The *Escherichia coli* phage T5 has long been regarded as a fascinating phage because of its unique mechanism of infection. T5 uses a two-step mechanism to deliver its 121 kb DNA into the host. After binding to its receptor FhuA, only 8 % of the genome enters the cell before the transfer temporarily stops. During the pause, expression of the pre-early genes encoded by this short DNA fragment has devastating effects on the host: the bacterial chromosome is degraded and the defense systems are inactivated. After a few minutes, T5 DNA transfer resumes allowing entry of the complete genome, and the expression of the middle and late genes of T5 is activated for phage production. Although this atypical mode of infection was described 60 years ago, very little is known about the molecular and cellular mechanisms that control the two-step DNA transfer and host takeover.

Our investigations into the structure of the T5 phage particle identified the tail-tip proteins forming the “cell-puncturing” device, which perforates the host envelope to enable DNA delivery. Moreover, our *in vitro* studies showed that binding of T5 to purified FhuA, in bulk or reconstituted into liposomes, results in complete DNA release with no pause. Thus the two-step transfer observed in vivo results from tightly regulated interactions between phage and host factors.

The sequencing of T5 and T5-related genomes revealed that only few of the pre-early genes are conserved and two genes, A1 and A2 were shown to be essential for infection. Both genes are required for resuming and completing phage DNA transfer and additionally, A1 controls the massive degradation of the host chromosome. We have recently characterized the structural and functional properties of the unusual and multitasking A1 protein, which is a manganese-dependent DNAse involved in the regulation of the phage DNA transfer.

O4 Genome replication and assembly of the bacteriophage SPP1 particle *in vivo*

AUDREY LABARDE[1], LINA JAKUTYTE[2], SILVIA AYORA[3], PRISHILA PONIEN[4], ERIC JACQUET[4], CYRILLE BILLAUDEAU[5], RUT CARBALLIDO-LOPEZ[5], PAULO TAVARES[1]

[1] Institut de Biologie Intégrative de la Cellule (I2BC), Department of Virology, Campus CNRS, Gif-sur-Yvette, France

[2] Virologie Moléculaire et Structurale, Campus CNRS, Gif-sur-Yvette, France

[3] Centro Nacional de Biotecnología, Departamento de Biotecnología Microbiana, CSIC, Madrid, Spain

[4] ICSN, Campus CNRS, Gif-sur-Yvette, France

[5] Micalis, INRA, Jouy-en-Josas, France

Corresponding author: paulo.tavares@i2bc.paris-saclay.fr

Tailed bacteriophages deliver their DNA naked to the cytoplasm of bacteria. Early genes expression leads to a fast takeover of host functions and viral genome replication. Assembly of virions follows to yield hundreds of infectious viral particles within 30-60 min in optimal conditions. The molecular mechanisms of phage DNA replication and virion assembly were extensively studied. However, much less is known how these processes are setup in the host cell with concomitant hijacking of bacterial resources and remodeling of the cytoplasmic space. We will describe studies on the temporal and spatial program of the viral replicon and phage particles assembly pathways in *Bacillus subtilis* cells infected with bacteriophage SPP1. SPP1 DNA replication is shown to occur in a defined focus where the host replisome is massively recruited. SPP1 procapsids partially co-localize with the phage replication factory but particles that packaged DNA rapidly accumulate in spatially distinct foci (warehouses).

SPP1 infection thus leads to a major remodeling of the host cytoplasm to optimize, most likely, the efficiency of phage multiplication.

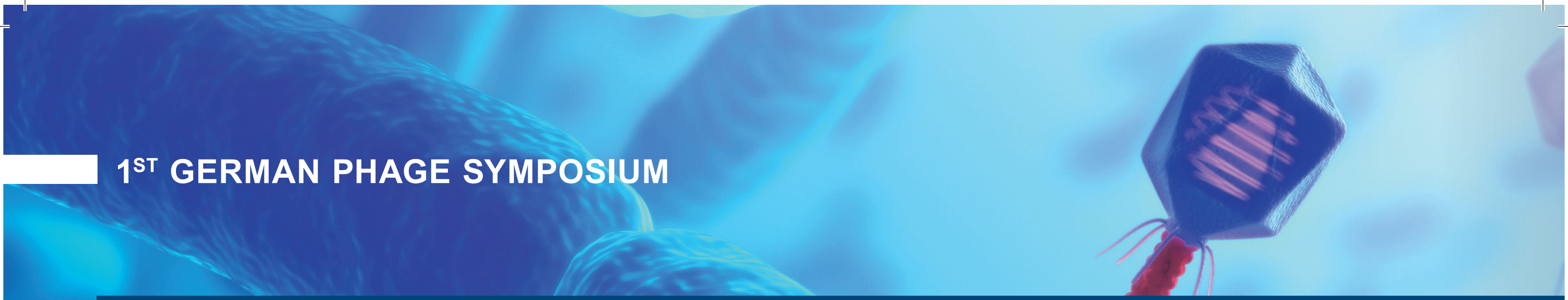

O5 Filamentous phage assembly

BELINDA LOH, MAX HAASE, LUKAS MUELLER, ANDREAS KUHN, SEBASTIAN LEPTIHN
Department of Microbiology & Molecular Biology, University of Hohenheim, Stuttgart, Germany
Corresponding author: sebastian.leptihn@uni-hohenheim.de

In contrast to lytic phages, filamentous phages are assembled in the inner membrane and secreted across the bacterial envelope without killing the host. Filamentous phages code for membrane-embedded proteins that allow the assembly and extrusion of the phage across the host cell wall. We characterized the M13 phage protein gp1 found in the inner membrane of the host and identified Walker A and B motifs with a conserved lysine in the Walker A motif (K14), and a glutamic and aspartic acid in the Walker B motif (D88, E89). Both, Walker A and Walker B, are essential for phage production. The crucial role of these key residues suggested that gp1 might represent a molecular motor driving phage assembly via the hydrolysis of ATP.
To confirm the findings and to characterize the membrane protein complex *in vitro*, we expressed and purified gp1. Even in absence of other phage proteins or phage DNA, gp1 hydrolyses ATP *in vitro*. Using electron microscopy, we could further demonstrate that gp1 and gp11, a N-terminally truncated internal start product of geneI, form a complex with a ring-like structure in the host membrane. Here, we present a model how filamentous phages are assembled.

O6 Not a barrier but a key: How O-antigen specific bacteriophages exploit lipopolysaccharide as an essential receptor to initiate infection of Gram-negative hosts

STEFANIE BARBIRZ
University of Potsdam, Physical Biochemistry, Germany
Corresponding author: barbirz@uni-potsdam.de

Tailed bacteriophages that infect gram-negative bacteria have to overcome the rigid barrier of lipopolysaccharide (LPS). The biochemistry of these early events in the infection cycle is not well understood, especially how host cell recognition is linked to conformational rearrangements in the tail that initiate particle opening. Whereas in many phages binding to an outer membrane protein receptor triggers release of the capsid contents, for O-antigen specific phages it is sufficient to contact the LPS [1]. We have established *in vitro* analyses of particle opening with a set of model bacteriophages infecting *Salmonella spp.* of O-serogroup *Typhimurium* [1-3]. These phages use their tailspike proteins to hydrolyze the O-antigen polysaccharide [4]. This step is a prerequisite for proper membrane attachment; accordingly they only infect smooth strains but not rough species lacking the O-antigen. We therefore propose a general infection model for O-antigen specific phages where a host specific O-antigen contact is linked to subsequent steps initiated by the inherent properties of the LPS outer membrane heterobilayer.

1. Andres D, Hanke C, Baxa U, Seul A, Barbirz S, Seckler R. Tailspike interactions with lipopolysaccharide effect DNA ejection from phage P22 particles in vitro. J Biol Chem 2010;285: 36768-75.
2. Broeker et al. Dettilon tailspike - one tool of myovirus Det7's swiss army knife to infect a broad host range. 2017. Manuscript in preparation.
3. Andres D, Roske Y, Doering C, Heinemann U, Seckler R, Barbirz S. Tail morphology controls DNA release in two *Salmonella* phages with one lipopolysaccharide receptor recognition system. Mol Microbiol 2012;83: 1244-53.
4. Broeker NK, Andres D, Kang Y, Gohlke U, Schmidt A, Kunstmann S, Santer M, Barbirz S. Complex carbohydrate recognition by proteins: Fundamental insights from bacteriophage cell adhesion systems. Perspectives in Science 2017;11: 45-52.

O7 The role of xenogeneic silencing in (pro-) phage-host interaction

EUGEN PFEIFER, MAX HÜNNEFELD, JULIA FRUNZKE
Forschungszentrum Jülich, Institute of Bio- and Geosciences IBG-1: Biotechnology, Germany
Corresponding author: j.frunzke@fz-juelich.de

In their prophage state, temperate bacteriophages are able to maintain a long-term association with their bacterial host. The acquisition of foreign DNA allows for beneficial mutual interactions but also bears a risk to the host potentially causing cell death by the expression of toxic phage genes. Hence, the integration of prophage elements into the genome and into host regulatory circuits requires a stringent regulation. The genome of the Gram-positive soil bacterium *Corynebacterium glutamicum* ATCC 13032 contains three prophages (CGP1-3). Among those, the large, cryptic prophage element CGP3 covers almost 6 % of the entire genome and is still inducible [1]. In recent studies we identified the small nucleoid-associated protein CgpS (Lsr2 homolog), which was shown to act as an essential silencer of cryptic prophage elements in *C. glutamicum* [2]. ChAP-Seq experiments in combination with EMSA studies revealed that CgpS binds to AT-rich DNA and represses gene expression of mainly horizontally acquired genomic regions. Counteraction of CgpS activity by overexpression of the N-terminal oligomerization domain resulted in a severe growth defect and a highly increased frequency of CGP3 induction leading to cell death. Beyond their role in the control of gene expression, recent preliminary data suggested an even broader function in chromosome organization and replication. As revealed by bioinformatics analysis CgpS homologs are found in almost all actinobacterial species but, remarkably, also in the genomes of several actinobacteriophages and prophages. Whereas sequence conservation is low, the highly conserved secondary structure highlights the ancient function of these proteins in (pro-)phage-host interaction.

1. Helfric, S, Pfeifer E, Krämer C, Sachs CC, Wiechert W, Kohlheyer D, Nöh K, Frunzke J. Live cell imaging of SOS and prophage dynamics in isogenic bacterial populations. Mol Microbiol 2015;98: 636-50.
2. Pfeifer E, Hünnefeld M, Popa O, Polen T, Kohlheyer D, Baumgart M, Frunzke J. Silencing of cryptic prophages in *Corynebacterium glutamicum*. Nucleic Acids Res 2016;44: 10117-31.

O8 Bacteriophages of *Staphylococcus aureus* and their impact on host evolution

CHRISTIANE WOLZ
University of Tübingen, Institute of Medical Microbiology and Hygiene, Germany
Corresponding author: Christiane.Wolz@med.uni-tuebingen.de

In *Staphylococci* horizontal gene transfer and species evolution are tightly linked to the activity of bacteriophages. Temperate *Staphylococcus aureus* phages can code for a variety of staphylococcal virulence factors which are important for the success of certain *S. aureus* strains. For instance, Sa-2 phages harbour genes encoding Panton-Valentine leukocidin which is associated with strains causing hard to treat superficial wound infections (especially community-associated Methicillin resistant *S. aureus* strains). Sa3-bacteriophages are present in 90 % of all *S. aureus* isolates of human origin: They integrate into the hlb-gene and therefore prevent the production of β-hemolysin. They provide genes coding for immune-evasion factors which are all highly human host specific and subsequently increase the pathogenicity of the lysogenized bacteria. However, during chronic lung infections in cystic fibrosis patients these phages are highly mobile. These phages are meanwhile rarely found in *S. aureus* strains of animal origin. There is limited knowledge about the molecular mechanisms involved in the integration and excision of temperate phages and also how other mobile genetic elements interfere with the biology of these phages. To this end, we established molecular tools to analyse the molecular basis for strain specific phage transfer. We could show that the bacterial strain background has a high impact on phage gene expression and phage biology. Deeper insights into phage biology will be beneficial for the understanding of bacterial evolution and host adaptation.

O9 Assess the role of viruses in contaminant biodegradation through metagenomics

LI DENG
Helmholtz Zentrum Muenchen – German Research Center for Environmental Health, Institute of Virology, Emmy Noether group leader „Viral Ecology", Neuherberg, Germany
Corresponding author: li.deng@helmholtz-muenchen.de

Viral communities are emerging as fundamental drivers of ecosystems by profoundly shaping microbial populations and processes that go beyond mortality and gene transfer to also include direct manipulation of metabolic pathways integral to host-cell function. Consequently, biogeochemical processes in microbial ecosystems and their potential for novel niche adaptation in response to changing environmental conditions can be understood only when this large dynamic gene pool carried by lytic and temporary viruses is recognized. However, little is known about this gene pool in groundwater ecosystem, especially in the contaminated groundwater.

The newly founded Emmy Noether project aims to elaborate a new perspective, the viral-driven degradation. We present here a powerful toolkit – from the concentration and purification of viral particles to the amplification of the resulting DNA for sequencing preparation – for studying environmental virus communities in the 'omics' era. In addition, "Viral-Tagging" is a high-throughput method to link wild viruses to specific host cells for screening and sequencing, thus allow for much greater access to the tagged viral community to study virus-host interactions in complex communities. We are equipped to study viral ecology by quantitatively linking objectively defined environmental viral populations, and their genomes, to their hosts, thus can better elucidate the processes that drive the population structure of virus and their host in nature. Using these new tools, we are currently studying the viral community in the contaminated groundwater, and our ultimate goal is to underline the mechanism of how viruses impacting contaminants degradation through (i) horizontally transfer host metabolic genes related to contaminant degradation, and (ii) specifically lysing key bacterial degraders.

O10 Bacteriophages of Shiga toxin producing *E. coli* – small molecules with high impact

HERBERT SCHMIDT
University of Hohenheim, Insitute of Food Science and Biotechnology, Department of Food Microbiology and Hygiene, Stuttgart, Germany
Corresponding author: herbert.schmidt@uni-hohenheim.de

Enterohemorrhagic *Escherichia coli* (EHEC) are the causative agents of hemorrhagic colitis and the hemolytic-uremic syndrome (HUS). The major pathogenicity factor of EHEC is the production of one or more Shiga toxins (Stx). Stx are generally encoded in the genome of lamboid prophages, which are integrated into distinct positions in the respective EHEC chromosome. Besides these Stx-phages, further non-Stx-encoding lambdoid prophages are present in the EHEC chromosomes in varying numbers. The foodborne EHEC O157:H7 strain EDL933 harbors 7-10 lamboid prophages, two them encoding Stx1 and Stx2. The stx genes are located in the lambdoid prophages in distinct positions close to the antiterminator Q in the late transcribed region. Upon induction of the prophages, *stx* is cotranscribed with the late transcribed phage genes.
In early studies, we could show that in many EHEC strains, a large open reading frame is located in 3'-direction close to the *stx* genes. These large open reading frames are homologues to the chromosomal *nanS* gene, which is present in the most pathogenic and apathogenic *E. coli* strains. NanS is an esterase, cleaving an acetyl residue from 5-N-acetyl-9-O-acetyl-neuraminic acid (Neu5,9Ac2). The remaining Neu5Ac can then be used as a carbon source.
Bioinformatic analysis has shown that in the chromosome of different EHEC strains large and varying numbers of *nanS* homologs are present, which we have designated *nanS*-p, a number of which have already been demonstrated to function as esterases. Growth experiments with *E. coli* O157:H7 strain EDL933 and isogenic mutants demonstrated that by using these enzymes, the strains are capable to grow well on Neu5,9Ac2, but mutants with deletion of all seven *nanS*-p alleles did not.
We hypothesize that multiple prophage-located *nanS*-p genes represent a mobile gene pool, ensuring the substrate utilization and therefore growth and maintenance of the infecting population in the large intestine where the mucus layer is thick and has a great turnover rate. Therefore, Stx- and non Stx-prophages of EHEC should be considered as a pathogenic principle of such EHEC strains causing serious diseases.

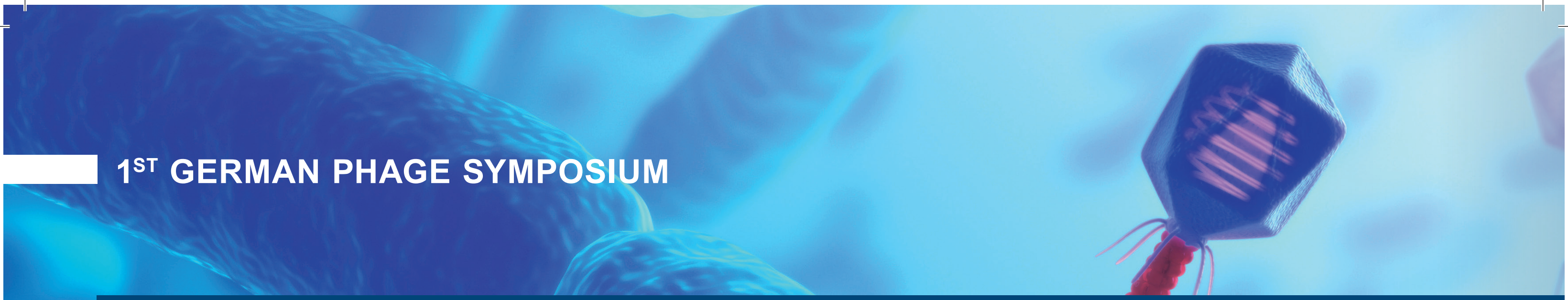

O11 Phage therapy: an alternative to antibiotics?

HANS-PETER HORZ
University Hospital RWTH Aachen, Institute of Medical Microbiology, Division of Virology, Germany
Corresponding author: hhorz@ukaachen.de

The potential of phages as alternatives to antibiotics (AB) against multi-drug resistant bacteria (MDR) is a matter of intensive debate. However, the breakthrough of phages as being officially accepted to fight human infectious diseases still faces many hurdles. Apart from conventional phage therapy there exist a number of anti-bacterial strategies involving phages or products encoded by them. This includes preventive approaches, such as removal of bacterial pathogens from critical hospital surfaces prior to the event of a nosocomial infection, the suppression of horizontal transfer of AB-resistance genes across bacterial species or even phage-mediated reversal of antibiotic resistance. The question whether or not phages are genuine alternatives to antibiotics thus cannot be answered in general terms as to some extent phages may help overcome the crisis with antibiotics rather than replacing them. For instance, in our ongoing studies we observe remarkable synergisms with phage and AB against bacteria which otherwise are resistant against the AB alone. Hence the combinations of different antibacterial agents may represent the clue for successful future approaches. These data along with a variation of other promising concepts making use of phage biological properties will be presented, particularly in the light of the emerging view of the overall presence and protective effect of naturally occurring phages within the human microbiome. Lastly, in order to realize human phage therapy in future it is also important to disentangle true concerns associated with phages from biased skepticism which often accompanies the discussion about the pros and cons of phage application.

O12 Tripartite species interaction: How phage/bacteria co-evolution impacts the virulence on an eukaryotic host

HEIKO LIESEGANG
Georg-August University of Göttingen, Institute of Microbiology and Genetics, Genomic and Applied Microbiology, Germany
Corresponding author: Heiko.Liesegang@biologie.uni-goettingen.de

The virulence of bacterial parasites is often associated with temperent phages that act as hyperparasites. By integrating into the bacterial genome as prophage the virus can introduce virulence factors (lysogenic conversion). Prophages can as well enter the lytic cycle and thereby kill the bacterial parasite. Thus phages can increase and as well decrease the pathogenicity in such a tripartite system. Not many systematic investigations on the phage induced virulence balance have been conducted due to the inherent complexity of the system. Here we present a study on the question how bacterial resistance on phages impacts the virulence on a eukaryotic host. We used a model system consisting of *Vibrio* Phages and *Vibrio alginolyticus* as phage/bacterial parasites and pipe fish (*Sygnatus typhle*) as host.
According to their resistance to phages we identified three classes of Bacteria highly susceptible (hs), intermediate susceptible (is) and resistant (r). We experimentally challenged pipefish with isolates of the different classes and investigated them on their impact on the viability, the fecundity and the survival of the fish. While the *Vibrio* counts on the fish did not differ on the different strains the gene expression of the fish clearly showed strain specific patterns. By challenging a culture of HS strains with phage solutions under co-evolutionary conditions immediately resistant mutants emerge.
Bacteria with a phage susceptible phenotype show an increased virulence on their host fish. Under appropriate conditions phage resistant less virulent parasites evolve. Hyperparasitism is thus an important factor for the virulence of bacterial pathogens.
Currently in detail comparative genome analysis of the evolved bacterial and viral genome is performed. Genome analysis of the phages, the bacterial pathogens and the evolved resistant strains will reveal the genotypes under selection that are responsible for the acquired phage resistance as well as for the modified virulence on the eukaryotic host.

O13 Elucidating phage-bacterium interactions that triggers changes in bacterial composition and functional profile in the gut of older adults

JOSUÉ L CASTRO-MEJÍA[1], RASMUS JACOBSEN[1], ŁUKASZ KRYCH[1], WITOLD KOT[2], LARS H HANSEN[2], SØREN REITELSEDER[3], LARS HOLM[3], SØREN B ENGELSEN[4], FINN K VOGENSEN[1], DENNIS S NIELSEN[1]

[1] Unversity of Copenhagen, Department of Food Science, Food Microbiology and Fermentation, Frederiksberg C, Denmark

[2] Aarhus University, Department of Environmental Science, Denmark

[3] University of Copenhagen, Department of Biomedical Sciences, Denmark

[4] University of Copenhagen, Chemometrics & Analytical Technology, Department of Food Science, Denmark

Corresponding author: jcame@food.ku.dk

Changes in the composition of the gut microbiome (GM) or dysbiosis are linked to a number of intestinal and extra-intestinal disorders, including age-related impairments associated with the metabolic syndrome. Several environmental factors may contribute to imbalances in GM composition, including diet, drugs and antibiotics administration and enteric pathogens. Less is known about the impact of the virome on the GM composition, its functionality and interactions with age-related comorbidities in older adults. Here, co-abundance correlation analysis on metagenome-sequencing of fecal concentrated-preparations of free virions and 16s-rRNA high-throughput sequencing demonstrated a large number of phage-bacterium interactions based on the core members (75 % ubiquity) from both microbial components. Strikingly, as a function of phage attack, the fluctuations in bacterial abundance resulted in dramatic variations in their global metabolic potential and interactions with host renal function, a condition that may rise from a cluster of risk factors associated with the metabolic syndrome. Together, our data demonstrate that members of the gut virome are able to trigger dysbiosis, entangling viral implications associated with age-related comorbidities.

O14 Phage therapy: from treating complications to targeting diseases

ANDRZEJ GÓRSKI, ROMAN MIEDZYBRODZKI, BEATA WEBER-DĄBROWSKA, SŁAWOMIR LETKIEWICZ, WOJCIECH FORTUNA, PAWEŁ ROGÓŻ
Polish Academy of Sciences, Institute of Immunology and Experimental Therapy, Wroclaw, Poland
Corresponding author: agorski@ikp.pl

The increasing antimicrobial resistance (AMR) is believed to be one of the greatest challenges of medicine and society today, exacerbated by the crisis in the search for new antibiotics. At the recent UN General Assembly (21 Sept 2016) WHO Director General compared AMR to "a slow motion tsunami" and added: "Doctors facing patients will have to say, sorry, there is nothing I can do for you". This crisis has revived interest in the potential use of phages – bacterial viruses – to combat AMR.

In 2005, a unit has been established at our Institute which has been carrying out phage treatment as experimental therapy (compassionate use, expanded access) in line with the current administrative, legislative and ethical requirements. Our laboratory and clinical data derived from that unit strongly suggest that phage therapy may be a safe and efficacious treatment in this clinical setting and good results can be achieved in as many as 40 % of cases that had been previously unsuccessfully treated with antibiotics. Antibody responses to phage treatment vary depending on the route of phage administration, are low in patients receiving phage orally and do not necessarily adversely affect therapy outcome. Experimental studies in mice indicate that phage and their proteins do not induce cytokines and may downregulate reactive oxygen species production. Similar findings have been noted in patients on phage therapy. Our data thus suggest that phage therapy – in addition to its well known antibacterial action may also have anti-inflammatory and immunomodulatory activities which may also be useful in clinical medicine. As we have recently pointed out while phage therapy today combats infections it has potential for evolving from merely a treatment for complications to targeting diseases [1]. What is more, our data support the notion that phages may have immunomodulatory functions which may have important clinical implications [2].

1. Górski A, Międzybrodzki R, Weber-Dąbrowska B, Fortuna W, Letkiewicz S, Rogóż P, Jończyk-Matysiak E, Dąbrowska K, Majewska J, Borysowski J. Phage therapy: Combating infections with potential for evolving from merely a treatment for complications to targeting diseases. Front Microbiol 2016; doi 10.3389/fmicb.2016.01515.
2. Górski A, Dąbrowska K, Międzybrodzki R, Weber-Dąbrowska B, Łusiak-Szelachowska M, Jończyk-Matysiak E, Borysowski J. Phages and immunomodulation. Fut Microbiol 2017; doi 10.2217/fmb-2017-0049.

O15 Bacteriophage therapy in lung infections

MARTIN WITZENRATH
Charité – Universitätsmedizin Berlin, Department of Infectious Diseases and Respiratory Medicine, Germany
Corresponding author: Martin.Witzenrath@charite.de

The emergence of multidrug-resistant (MDR) bacteria is a great challenge for modern medicine. The rise of new resistance mechanisms is alarming, and MDR bacteria are spreading globally. Nosocomial pneumonia is increasingly caused by MDR bacteria, for which therapeutic options are lacking. Rediscovery of phage therapy may be a solution to the increasing failure of antibiotics. The advantage of phage-based therapy in treating lung infections was shown experimentally with bacteriophage endolysins, including Cpl-1. The inhalative or intraperitoneal application of Cpl-1 was a save and efficient therapy in severe pneumococcal pneumonia in mice. Whereas *S. pneumoniae* is the major cause of community-acquired pneumonia, *A. baumannii* is a common cause of nosocomial infections, mostly hospital-acquired pneumonia, and frequently possesses resistance against broad-spectrum antibiotics. Mice, infected with *A. baumannii* and treated with a purified phage preparation intratracheally, showed significantly reduced bacterial load in bronchoalveolar lavage fluid and lung as well as a significantly improved clinical outcome and lung permeability. Neither cellular nor humoral unwanted effects of phages were observed. These data further support the concept of developing a phage-based therapy against pulmonary *A. baumannii* infections.

In addition, chronic lung diseases are uprising and complicated by airway infections. Patients suffering from pre-impaired lungs are often chronically infected with *P. aeruginosa*, especially patients with cystic fibrosis or "non-CF" bronchiectasis. A funding application was submitted to the German Federal Ministry of Education and Research (BMBF) in 2017 to realize development of a phage preparation approvable in Germany and the EU and a clinical trial to test safety, tolerability and efficacy in healthy volunteers and patients with chronic pulmonary *P. aeruginosa* infection. After receiving notification about a positive evaluation, the project partners now aim at establishing phages as therapeutic agent for *P. aeruginosa* infections.

O16 European Project PHAGOBURN: The first randomized, single blinded, multi centric and controlled clinical trial in Human Phage therapy: Design and objectives.

PATRICK JAULT
Percy Military Hospital, Clamart, France
Corresponding author: jaultpatrick@gmail.com

Antibiotic resistance has become in few years a major public health issue. WHO estimates that by 2050 infectious diseases will be the leading cause of death worldwide. Despite many policies of prudent use of antibiotics, the incidence of infection by MDR bacteria is still increasing. Therefore, it becomes necessary to find innovative therapeutic alternatives.

PHAGOBURN aimed to asses two objectives, first the efficacy of a cocktail of bacteriophages to reduce the bacterial burden on human burn wounds infected by *Pseudomonas aeruginosa* in comparison with a control group treated by Silver Sulfadiazine, the second to observe the safety of the cocktail.

The project was sponsored by PHERECYDES PHARMA a French Small-Medium Entreprise. The European Commission grated the project for 3.8 Millions Euros. The French Military Health Service oversaw coordination of the project.

Cocktails were developed in research and development by Pherecydes-Pharma, the bio-production in GMP environment was operated by CLEAN-CELLS (France) and inspected by the French Medicine Agency (ANSM).

Eleven investigator centers in Europe were involved in the clinical trial, 3 in Belgium, 1 in Switzerland and 7 in France. The first patient was included in July 2015 and the last in December 2016. The duration of the project was 3 years but benefited of two extensions time to a total length of 48 months. In July 2017, the analyses of the final results are still in progress. The initial target of 110 patients was not reachable for several reasons. Elaborating and operating a full process of a first bio-production chain and its quality control and the validation by a national regulation agency took 24 months.

PHAGOBURN opens the way for further multi centric controlled randomized studies in several fields from research and development of new cocktails to all infectious diseases in human and veterinary medicine.

O17 Phagetherapy: History, working and use in clinical practice

THOMAS ROSE[1], SERGE JENNES[1], DANIEL DE VOS[2], GILBERT VERBEKEN[2], JEAN-PAUL PIRNAY[2]
[1] Queen Astrid Military Hospital, Burn Wound Centre, Brussels, Belgium
[2] Queen Astrid Military Hospital, LabMCT, Burn Wound Centre, Brussels, Belgium
Corresponding author: thomasrose@telenet.be

Felix d'Herelle, one of the discoverers of bacteriophages was the first to propose "Phagetherapy" already in the early 20th century. It was further developed and used in medical practice in all the previous Soviet Republics till now. In the Western world however antibiotics were developed and Phagetherapy was almost forgotten.
Today, seen the antibiotic resistance problematic worldwide, Phagetherapy is back in the picture as a potential complementary or alternative approach in the fight against multi drug resistant infectious bacteria.
The main problem is a lack of evidence based studies in accordance to modern standards as well as the lack of an adapted regulatory frame. So initiating studies in humans in accordance to actual ethical regulatory and scientific standards is difficult. Phagetherapy is applied today in Europe under the Helsinki Declaration, which is not a final solution for common clinical application neither for progressing the scientific way.
Although these difficulties several groups setup studies, while the idea of using phages as antibacterials has already reached the stage of applications in the food and agrobio industry. The application in the clinic is imminent at least at the stage of clinical studies which are urgently needed in the fight against bacterial infections.
We initiated a clinical safety study in burn patients approved by a leading medical ethical committee and published the method for preparing the Phagetherapy cocktail in use as well as the ways to approach it in the regulatory context.
We want to present some different cases of our clinical daily work in which the use of Phagetherapy cocktail, used under the Helsinki formula, has shown its benefit.

O18 Efficiency of Bacteriophage Therapy in Children with Diarrheal Diseases

KARAMAN PAGAVA[1], RATI GOLIJASHVILI[2], SOPO GHOLADZE[2], NANA JAPARASHVILI[2], EKATERINE CHKHARTISHVILI[1]

[1] Tbilisi State Medical University, Pediatrics, Georgia

[2] Biochimpharm JSC, Georgia

Corresponding author: kpagava@yahoo.com

The emergence of antimicrobial resistance becomes a critical problem in modern medicine. Bacteriophage Therapy (BT) is considered as one of the best tools allowing decreasing essentially the usage of antibiotics. The paucity of appropriately conducted, placebo-controlled clinical trials is interfering the implementation of BT in clinical praxis.

Aim of the study: carrying out the clinical trial on BT of diarrheal diseases in children.

Methods. A double-blind randomized placebo-controlled clinical trial was performed in 71 hospitalized children (6 months to 6 years old) with diarrhea. 37 patients had moderate diarrhea (moderate intoxication, fever 37.5-39 °C, calprotectin positive – 12/37, bacteriological culture positive - 0/9), 34 - severe diarrhea (severe intoxication, fever 39-40 °C, calprotectin positive – 33/34, bacteriological culture positive – 16/24). Duration of stay in the hospital and improvement of the integrative index of severity in 48 hours after the beginning of bacteriophage/placebo treatment were compared. 36 patients received treatment according to guidelines plus placebo, 35 - instead of placebo received polyvalent bacteriophage SEPTAPHAGE manufactured by BIOCHIMPHARM JSC. Duration of stay in the hospital and improvement of the integrative index of severity in 48 hours after the beginning of bacteriophage/placebo treatment were compared.

Results. BT significantly shortened duration of stay in the hospital (on average for 1.9±0.6 days); prevented deterioration of the clinical course of disease (especially in case of positive test on calprotectin) and switching to the antibiotic therapy if it was not administered according to the guidelines on admission; improved the integrative index of severity by 39.2±5.7 degree.

Conclusions. BT with SEPTAPHAGE improved clinical course of diarrhea in hospitalized children aged from 6 months to 6 years.

O19 Application of lysines as an alternative to bacteriophages (Designer Lysine)

WOLFGANG MUTTER
HYPharm GmbH, Bernried am Starnberger See, Germany
Corresponding author: wolfgang.mutter@hyglos.de

To escape from their bacterial host phages produce a lytic enzyme (lysine) which is able in the late stage of replication to destabilize the bacterial cell wall. Finally, due to the intracellular pressure, the bacteria is destroyed and the phages escape into the extracellular environment.
In the case of gram-positive bacteria, the lysine is able to destabilize the bacterial cell-wall also from the outside. In this case lysines could be used for selective elimination of certain bacteria.
The lysines normally consist of two protein domains: one responsible for specific binding to the bacteria (CBD = cell binding domain), the other domain contains the enzymatic activity (EAD = enzymatic active domain) which is able to cut between amino acid and sugar residues. The molecular weight of the proteins is between 20 and 40 kD.
Due to their activity, lysines could be used as an alternative antimicrobial. They are as efficient as antibiotics, have a comparable MIC and work much faster. However, they could not be used for *in vivo* application as they are immunogenic and the immune system generates anti-lysine antibodies.
The application on surfaces (skin, gut) is possible, requiring that the proteins have to be optimized regarding stability and expression rate.
One lysine-molecule (HY-133) which is directed against *Staphylococcus aureus* is currently in GMP production; clinical phase I trials are planned for 2019.

O20 Characterization of *Campylobacter* phages and their application

STEFAN T. HERTWIG
German Federal Institute for Risk Assessment, Berlin, Germany
Corresponding author: Stefan.Hertwig@bfr.bund.de

Campylobacter is an important foodborne pathogen worldwide. The genus comprises 25 species of which the thermophilic *C. jejuni* and *C. coli* are the most common causes of acute bacterial enteritis. Human infections occur mainly by the consumption of undercooked poultry. Virulent phages are a promising tool to reduce pathogenic bacteria along the food chain. Though, whereas lytic phages have already been used to reduce the numbers of *Campylobacter* in chicken, only little is known about the genetics of these phages. The analysis of *Campylobacter* phage DNA is hampered by unusual DNA modifications. According to their genome size estimated by PFGE, *Campylobacter* phages have been allocated to three groups, of which group II (180 kb) und group III (140 kb) are the most common. We isolated six group II and five group III phages from the environment and from food samples, all of them belong to the family *Myoviridae*. While group II phages lysed strains of *C. jejuni* and *C. coli*, group III phages exclusively infected *C. jejuni*. However, group III phages generally lysed more *C. jejuni* strains than group II phages. In addition, the *in vitro* kinetics of cell lysis diverged in the two groups, probably caused by the different burst size of the phages. Restriction analyses and Southern hybridization revealed a close relationship of phages belonging to each group. By contrast, group II and group III showed almost no DNA homology. We sequenced one group II (CP21) and one group III (CP81) phage. Both phages are distantly related to T4-like phages. No virulence-associated genes were detected on the genomes. The genome of group II phages is composed of large modules separated by long DNA repeats, which may be arranged differently. On the basis of the genome organization two subgroups of group II phages exhibiting different host ranges were assigned.

O21 Impact of phages in dairy industry

HORST NEVE
Max Rubner-Institut, Federal Research Institute of Nutrition and Food, Department of Microbiology and Biotechnology, Kiel, Germany
Corresponding author: Horst.Neve@mri.bund.de

All fermentation processes in dairies rely on actively growing lactic acid bacteria as starter cultures. However, bacteriophages infecting these cultures may cause serious fermentation delays or even complete failures. *Lactococcus lactis* and *Streptococcus thermophilus* are prominent bacteria used in mesophilic and thermophilic starter cultures and can be attacked by a broad range of diverse phage populations. For *L. lactis*, 10 different phage groups are currently known, and many lactococcal strains do also contain prophages that can be released as temperate phages either spontaneously, or by induction with mitomycin C. New *S. thermophilus* phage populations have recently been identified as hybrid phages with genome regions derived from lactococcal and from *S. thermophilus* phages. Phages attacking flavor-producing *Leuconostoc* strains are also common in dairies.

Lactococcal phages of the widespread 936 group may possess intrinsic high thermal stabilities, and pasteurization of raw milk is therefore not a hurdle for these phage derivatives. Recycling of whey or whey components (contaminated by thermo-resistant phages) in subsequent fermentation processes requires non-thermal treatments for minimizing phage titers, e.g. by membrane filtration. Phages of *L. lactis*, *S. thermophilus* and of dairy *Leuconostoc* strains are also present in high titers in spray-dried whey powders, and a remarkable long-term stability has been shown for lactococcal phages in such samples which were stored for several years. Lactococcal phages of the 936 group are clearly dominating in whey and whey powder samples. This, however, may not be the case for raw milk samples where non-936 lactococcal phages were frequently detected in titers of 10 – 10^6 plaque-forming units per ml. Hence, raw milk can also be considered as a reservoir of new atypical phage populations.

O22 Reduction of *Campylobacter* load in broiler chickens by using phage application

SOPHIE KITTLER[1], SAMUEL FISCHER[2], GERHARD GLÜNDER[2]
[1] University of Veterinary Medicine Hannover, Reseach Center for Emerging Infections and Zoonoses (RIZ), Institute of Food Quality and Food Safety, Hannover, Germany
[2] University of Veterinary Medicine Hannover, Clinic for Poultry, Hannover, Germany
Corresponding author: Sophie.Kittler@tiho-hannover.de

Campylobacteriosis is the most common bacterial food-borne zoonosis in Europe. *Campylobacter spp.* are commensals in the avian gut and can contaminate broiler meat during slaughter. Risk assessments consider the reduction of *Campylobacter* in primary production to be most beneficial for human health. The aim of this study was to gain more detailed knowledge about the potential of bacteriophages in reducing *Campylobacter* colonization of broilers. A phage cocktail of four phages for *Campylobacter* reduction was tested in three in vivo trials under experimental conditions and in commercial broiler houses during the first field trials using phage application against *Campylobacter*. Significant reductions of *Campylobacter* counts were detected in all trials under experimental conditions and in two field trials. Reductions of up to log10 3.2 CFU in *Campylobacter* load at slaughter were detected in one field trial and one day after phage application, *Campylobacter* counts of one experimental group were reduced under the detection limit (< 50 CFU/g, P=0.0140) in fecal samples. Subsequently, the susceptibility to phage infection of the *Campylobacter* population in samples of all trials was determined by examining ten re-isolates per sample. Resistance analyses of isolates deriving from the *in vivo* trials under experimental conditions revealed that if a resistant or less susceptible *Campylobacter* subpopulation was present, levels of *Campylobacter* colonization were still reduced compared to phage free control birds. We could show for the first time that resistances of *Campylobacter* against phages stabilized at a low level after an initial increase. Resistance analyses of *Campylobacter* isolates from the field trials indicate that a phage susceptible *C. jejuni* subpopulation with increased motility and GGT activity could overgrow a non- susceptible subpopulation in the presence of phages. Considering our findings, we believe that phage bio-control could play a promising role in combating *Campylobacter* at farm level and thus in reducing human campylobacteriosis.

O23 Bacteriophage application in honeybees infected with *Paenibacillus larvae*

HANNES BEIMS
Lower Saxony State Office for Consumer Protection and Food Safety, Institute for Apiculture Celle, Germany
Corresponding author: Hannes.Beims@laves.niedersachsen.de

The American Foulbrood (AFB) is caused by the Gram-positive bacterium *Paenibacillus larvae*. Larvae of honeybees (*Apis mellifera*) are infected by incorporation of spores of this pathogen. Spores germinate in the midgut and kill the host by using different virulence mechanisms. After the killing of the host, the entomopathogen starts to digest the larvae. In a last step of the infection cycle the residues, called foulbrood scale, consisting mainly of *P. larvae*-spores, are removed and spread by the bees. In order to prevent antibiotic residues in honey it is not allowed to treat infected bee colonies with antibiotics in Germany. In case of an infection colonies have to be sanitized by laborious treatments. A possible alternative to these treatments might be the phage therapy.

P. larvae-specific bacteriophages were isolated from environmental samples and thoroughly characterized. The bacteriolytic activity of the phages was shown in plaque assays. Furthermore, the growth inhibition was shown against all four genotypes of *P. larvae* (ERIC I – IV) and 40 field isolates of the genotypes ERIC I and II in growth experiments. *In vivo* bioexposure assays showed that the feeding of bee larvae with these bacteriophages has no negative effect on the development of the brood. Moreover, the mortality of the bee larvae, infected with *P. larvae* ERIC I and II was reduced by the application of the phages.
Taken together these results demonstrated the potential of phage therapy as an effective biological treatment strategy against AFB.

O24 Characterization and applications of bacteriophage-derived peptidoglycan hydrolase enzymes targeting MRSA and antibiotic resistant *Clostridium difficile*

AIDAN COFFEY
Cork Institute of Technology, Department of Biological Sciences, Bishopstown, Ireland
Corresponding author: aidan.coffey@cit.ie

Staphylococcus aureus is a major cause of infection in humans and animals causing a wide variety of conditions from local inflammations to fatal sepsis. The bacterium is commonly multi-drug resistant and thus many front-line antibiotics have been rendered practically useless for treating human infections. Bacteriophages (phages) are viruses that are natural predators of bactéria [1]. We sequenced the genomes of three anti-staphylococcal phages, and then cloned the genes for the different peptidoglycan-degrading hydrolases, enzymes that degrade the bacterial cell wall during phage infection of bacterial cells. One of the enzymes, a phage endolysin was found to have a modular organisation with three domains, a cysteine/histidine-dependent amido hydrolase peptidase (CHAPk), an amidase, and thirdly a cell-wall binding domain [2, 3]. The latter facilitates attachment of the enzyme to the bacterial cell wall, while former two domains catalyse the degradation of the peptidoglycan, mediating rapid bacterial cell death. Deletion analysis of the enzyme showed that full lytic activity against live antibiotic-resistant staphylococci was retained when the endolysin was truncated to its CHAPk (peptidase) domain. The enzyme was purified by ion-exchange chromatography and characterized in detail including a high-resolution crystal structure obtained as native crystals diffracted to a maximum resolution of 1.8 Å. Addition of the enzyme to a turbid bacterial MRSA culture resulted in rapid elimination of turbidity. The peptidase was used in *in vivo* studies in mouse models where it successfully eliminated MRSA colonization in animals without adverse effects; and furthermore, *ex vivo* studies confirmed a low immunogenicity [2]. In a parallel approach, an active amidase endolysin was also cloned from the genome of a *Clostridium difficile* bacteriophage CD6356 and subjected to similar analysis. This enzyme has been cloned and expressed in a gut bacterium with the aim of delivering the agent to the site of *Clostridium difficile* infections.

1. O'Flaherty S, Ross RP, & Coffey A. Bacteriophage and their lysins for elimination of infectious bacteria. FEMS Microbiol Rev 2009;33: 801-819.
2. Keary R, Sanz-Gaitero M, van Raaij MJ, O'Mahony J, Fenton M, McAuliffe O, Hill C, Ross RP & Coffey A. Characterization of a Bacteriophage-derived Murein Peptidase for Elimination of Antibiotic Resistant *Staphylococcus aureus*. Curr Protein Pept Sci 2015;17:183-190.
3. Fenton M, Casey PG, Hill C, Gahan CGM, Ross RP, McAuliffe O, O'Mahony J, Maher F, & Coffey A. The truncated phage lysin CHAPk eliminates *Staphylococcus aureus* in the nares of mice. Bioengineered Bugs 2010;1: 1-4.

O25 Carrier systems for phages to supplement food systems

MEIKE SAMTLEBE[1], SYLVAIN DENIS[2], SANDRINE CHALANCON[2], ZEYNEP ATAMER[1], NATALIA WAGNER[3], HORST NEVE[3], CHARLES FRANZ[3], STÉPHANIE BLANQUET-DIOT[2], JÖRG HINRICHS[1]

[1] University of Hohenheim, Institute of Food Science and Biotechnology, Department of Soft Matter Science and Dairy Technology, Stuttgart, Germany

[2] Université Clermont Auvergne, UMR UCA-INRA MEDIS, Microbiology, Digestive Environment and Health, Clermont Ferrand, France

[3] Max Rubner-Institut, Federal Research Institute of Nutrition and Food, Department of Microbiology and Biotechnology, Kiel, Germany

Corresponding author: samtlebe@uni-hohenheim.de

Interest in the use of bacteriophages as antibacterial agents is nowadays increasing. One area where bacteriophages are particularly important and where they used as nonmedical application is in food processing. Phages reveal a number of valuable properties, as they are i) ubiquitous in the environment, ii) can be easily isolated and propagated and iii) have specific host ranges. The human gut contains about 10^{15} individual phage particles. However, little is known about the gut phageome and the impact on gut microbiota, health and diseases. It is obvious that phages may modulate the human gut microbiota. They can therefore indirectly influence the natural microbiota and maintain its balance. However, the targeted application of phages in the human gastrointestinal tract faces numerous challenges, i.e., their limited host ranges, bacterial resistances to phages, manufacturing issues, delivery systems and sensitivity to gastrointestinal conditions.

Our hypothesis is that phages that are specifically integrated into food matrixes may shape and modulate the microbiota associated with the human gastrointestinal tract. Hence, a preliminary research study aimed at encapsulating phages to improve their viability under gastrointestinal acid conditions and, hence, delivering them to the intestine in active form. *Lactococcus lactis* phages were selected as a simple model system. The effects of different encapsulation techniques, gastrointestinal pH and enzymes were investigated in *in vitro* experiments simulating human digestive conditions. The data obtained indicated that - in comparison to free phages - encapsulated phages are not inactivated during their transit of the stomach. Furthermore, under simulated intestine conditions, an effective release of phages from the capsules was achieved. In a subsequent study, the stability of encapsulated and non-encapsulated phages was analyzed in a dynamic in vitro gastrointestinal model simulating conditions of the human upper gastrointestinal tract. First results of this study will be presented and discussed.

O26 Phage therapy in diarrhea patients – experiences from studies in Bangladesh

HARALD BRÜSSOW
Nestlé Research Center, Lausanne, Switzerland
Corresponding author: harald.bruessow@rdls.nestle.com

Antibiotic resistance is rising in important bacterial pathogens. Phage therapy (PT) is a potential alternative. T4-like coliphages, a Russian coliphage product (total dose of 109 phages) or placebo was orally given to Bangladeshi children. Children were hospitalized at the International Center for Diarrheal Diseases Research in Dhaka for acute bacterial diarrhea. Sixty per cent of the children suffered from a microbiologically proven *E. coli* diarrhea; the most frequent diagnosis was enterotoxigenic *E. coli* (ETEC) infections. Other bacterial co-pathogens were detected. Half of the patients contained phage-susceptible *E. coli* colonies in the stool. No adverse events attributable to oral phage application were observed. Fecal coliphage titer was increased in treated over control children. Titer increases were weaker for T4 phages than for the Russian cocktail, containing many different phage types by metagenome analysis. Overall, the titers did not reflect substantial *in vivo* intestinal phage replication. Notably, *E. coli* represented less than 5 % of fecal bacteria. Stool ETEC titers showed only a short-lived peak and were with an average titer of 10^5/g stool close to the replication threshold for T4 phage. An interim analysis after the enrollment of 120 patients showed no amelioration in quantitative diarrhea parameter by PT over placebo. All patients received standard care (oral rehydration and zinc supplements). Stool microbiota was characterized by an overgrowth with commensal streptococci during the acute phase of diarrhea. Streptococcal abundance correlated with quantitative diarrhea outcome, but genome sequencing did not identify virulence genes. Subsequent screening of diarrhea patients revealed that antibiotic-treated malnourished diarrhea patients showed an expansion of fecal *E. coli*. *E. coli* abundance in these patients was inversely related to the abundance of *E. coli* Myovirus phAPEC8, suggesting *in vivo* amplification of endogeneous coliphages when intestinal *E. coli* crosses a concentration threshold.

O27 Phages to combat *Listeria* and *Salmonella*

STEVEN HAGENS
Micreos BV, Wageningen, The Netherlands
Corresponding author: s.hagens@micreos.com

Bacteriophages are a novel tool in food-safety. Not all phages are suitable for bio-control and criteria that need to be met will be discussed on the basis of two examples, a single phage product against *Listeria* and a two phage cocktail effective against *Salmonella*. Laboratory data demonstrating the efficacy of phages will be discussed. Food manufacturers need to comply with rules governing the presence of these pathogens in food and the possibilities of integrating phages into these frameworks will be shown. The issue of resistance will be addressed. It will be shown that phages cannot mask bad hygiene and that phage use certainly cannot replace hygiene in any way. Lastly, the transition from laboratory bench and the challenges thereof will be discussed, from a simple application such as in smeared cheeses to treatment of composite foodstuffs. This will show both possibilities as well as limitations of using phages as bio-control agents in food manufacture.

O28 Pros and cons of phage applications

CHRISTINE ROHDE
Leibniz Institute DSMZ – German Collection of Microorganisms and Cell Cultures, Braunschweig, Germany
Corresponding author: chr@dsmz.de

Pros and cons of phage applications should not only be discussed in comparison to antibiotics but in broader context. Nature is an inexhaustible resource of bacteriophages (phages) for most bacteria, phage use is appreciated where bacteria are unwanted. Phages are stable survivalists co-evolving with bacterial hosts and keeping natural balances, also in our microbiome. As the antibiotic multidrug resistance (MDR) crisis is a serious global problem, phages as biological alternatives are increasingly well-received. If a phage preparation is fully characterized, purified and targets a specific bacterial isolate, it may be used as tailor-made medicine without side-effects. Phages are self-controlling dependent on their bacterial hosts' presence. Phage therapy could be re-introduced in the western world more straightforward especially targeting the most frequent ESKAPE bacteria. This should not only be considered in ICUs or when multimorbidity becomes life-threatening but also more routinely to avoid antibiotics. But, tailor-made phage therapy only becomes realistic if a model licensing pathway for phage preparations is defined, accepted and streamlined by the authorities, optimally across national borders. Phage use doesn't mean products but a flexible concept reacting to specific infections. Monophage preparations or mixtures may be adapted to enlarge host spectra. Phages will probably be comparably lower-priced. Nevertheless, like all other medication, phage application should be farseeingly careful. Phages cannot replace antibiotics, both should complement each other. Knowledge on phage biology and phage-host interaction accumulated as demonstrated by impressive numbers of international publications. Why doesn't reality keep pace with scientific progress? Apart from the licensing pathway and unquestionable difficulties in IP protection, infrastructures are yet to be established: phage banks, pharmaceutical production facilities, diagnostic laboratories and hospitals conversant with phage use. It is necessary to discuss on broad level and with the EMA how phage application can be regulated efficiently whilst clinical trials should be granted.

1ST GERMAN PHAGE SYMPOSIUM

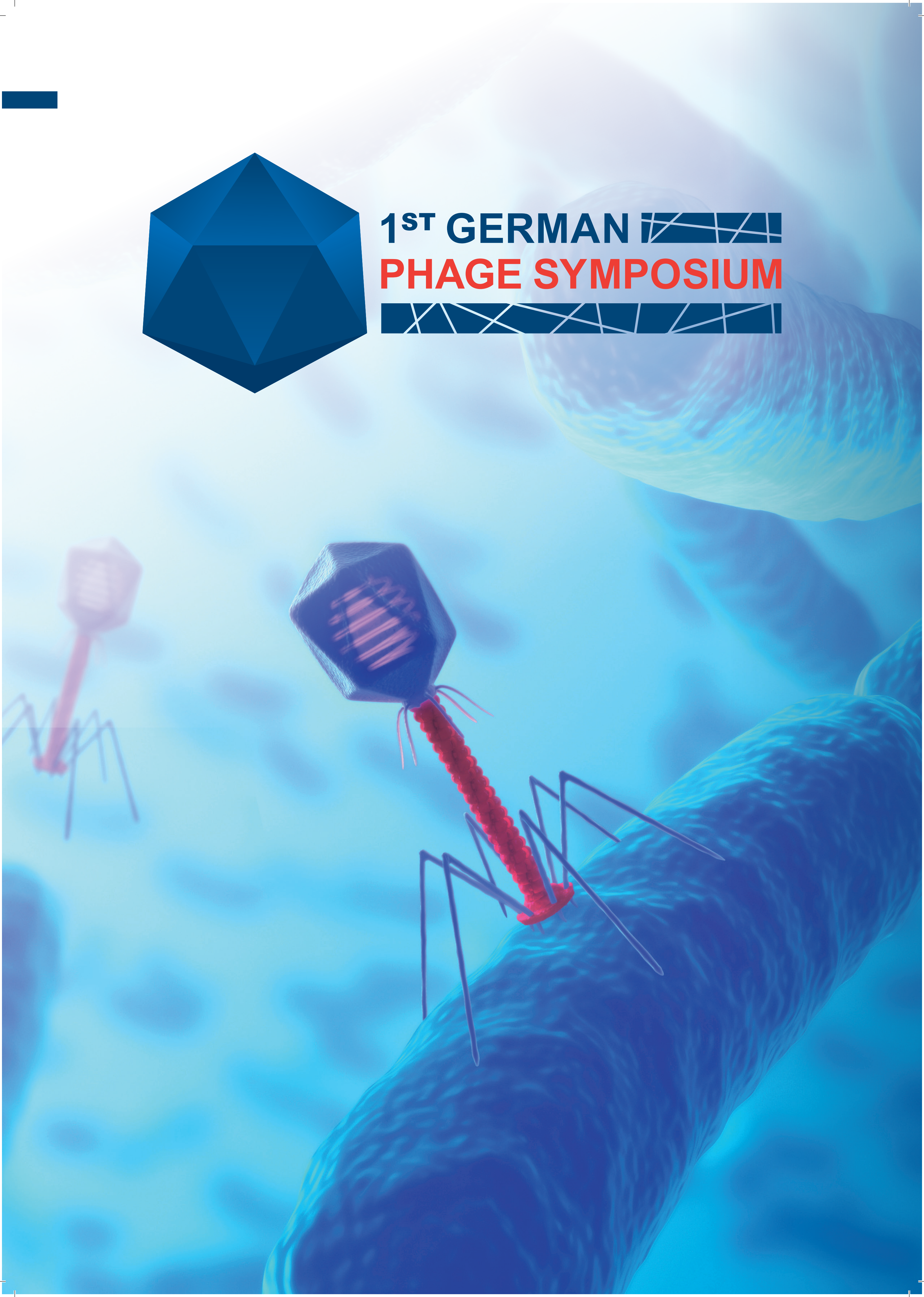

ABSTRACTS

POSTER

P1 Control and maintenance of prophages in *Salmonella enterica*

ASTRID WAHL
CNRS, LCB, Marseille, France
Corresponding author: awahl@imm.cnrs.fr

Thanks to increasing genomic data, the importance of bacteriophages is highlighted in horizontal gene transfer (HGT). Bacterial genomes typically harbor multiple prophages carrying a wide range of genes that potentially provide new abilities. On one hand, these genetic elements may be selected because they confer an ecological advantage, such as new virulence factors or adaptive traits [1]. On the other hand, uncontrolled expression of prophage genes could be detrimental. Thus, genome evolution by HGT requires a precise equilibrium between the repression and expression of newly acquired genes.

Salmonella enterica serovar *Typhimurium* is a primary enteric pathogen. Usually, *S. enterica* strains possess 4-5 functional prophages and prophage genes make up ca. 30 % of the pool of accessory genes [1]. Of note, some prophages are known to provide virulence factors to the host [2]. Our goal is to identify general strategies, other than phage repressor based, that are used to maintain prophages in the host genome. In this context, we have previously shown that the transcription terminator Rho is involved in prophage maintenance in *E. coli* [3].

A combination of targeted (mutants of major transcription regulators) and global (transcriptomics) approaches will help to identify (i) which host factors are involved in prophage maintenance and (ii) how pleiotropic regulators act on prophage gene expression and excision process in *Salmonella*. To do so, we have already tested various regulator mutants using multiplex and quantitative PCR. These preliminary results have shown that prophage maintenance is linked to both gene silencing and cell metabolism. Understanding how the host prevents prophage induction and controls the lysogenic conversion will give a clue on the mechanisms involved in cooptation and co-evolution of prophages and their host.

1. Bobay LM, Rocha EP, Touchon M. The adaptation of temperate bacteriophages to their host genomes. Mol Biol Evol. 2013;30:737-51.
2. Figueroa-Bossi N1, Bossi L. Inducible prophages contribute to *Salmonella* virulence in mice. Mol Microbiol. 1999;33:167-76.
3. Menouni R, Champ S, Espinosa L, Boudvillain M, Ansaldi M. Transcription termination controls prophage maintenance in *Escherichia coli* genomes. Proc Natl Acad Sci U S A. 2013;110:14414-9.

P2 Phage-host interactions: what have we learned from studying *Campylobacter* phages

LONE BRØNSTED, MARTINE HOLST SØRENSEN, Y. EMRE GENCAY
Unversity of Copenhagen, Veterinary and Animal Sciences, Frederiksberg C, Demark
Corresponding author: lobr@sund.ku.dk

Campylobacter jejuni remains to be the leading cause of bacterial foodborne illness in the western world and is seriously affecting child health and mortality in developing countries. *Campylobacter* phages are members of the Myoviridae and belong to the two genera of the Eucampyvirinae subfamily; Cp220virus and Cp8virus. Using our large phage collection, we showed that *Campylobacter* phages are either dependent on CPS or motile flagella for infection. Interestingly, the receptor type dependency correlated with the phage genus; Cp220virus are dependent on motile flagella, whereas Cp8virus rely on CPS for infection. While the actual receptor for flagelloptropic phages is under investigation, further analysis of the CPS-dependent phage F336 identified the MeOPN modification of GalfNAc present in the capsular polysaccharide (CPS) as a phage receptor in strain NCTC11168. This lead to the hypothesis that MeOPN is a common receptor for all CPS-dependent phages. To test this, we deleted the only MeOPN transferase gene in strain NCTC12662 sensitive to all our CPS-dependent phages. By HR-MAS NMR we showed that the mutant is deficient of MeOPN of the CPS and seven phages did not form any plaques on this strain. On the other hand, 33 phages infected the mutant, although at a lower efficiency compared to the wild type. To investigate the phage diversity further and identify receptor-binding proteins all our CPS phages are currently being sequenced using PacBio. Nevertheless, all our CPS-dependent phages were affected by the lack of MeOPN, indicating the vast importance of this unique surface modification for phage infection of *C. jejuni*.

P3 Different morphologies show common themes: How bacteriophages conquer the LPS-barrier of Gram-negatives

NINA BRÖKER[1], MAREIKE MEYER[1], YVETTE ROSKE[2], UDO HEINEMANN[2], STEFANIE BARBIRZ[1]
[1] University of Potsdam, Physical Biochemistry, Germany
[2] Max Delbrück Center for Molecular Medicine in the Helmholtz Association (MDC), Berlin, Germany
Corresponding author: nina.broeker@uni-potsdam.de

Bacteriophages of Gram-negatives encounter a membrane environment that is dominated by the complex properties of the LPS glycolipid molecule. Many phages exclusively infect hosts with LPS carrying long O-antigen polysaccharide. These O-antigen specific phages are equipped with tailspike proteins (TSP) which recognize and cleave the O-antigen. However, the role of this initial step for the subsequent events in the infection cycle is not well understood. We have previously shown that the enzymatic activity of phage TSP as well as a sufficient length of O-antigen chains in LPS is a prerequisite for successful DNA-ejection in podovirus P22 and siphovirus 9NA. In order to further analyze the molecular trigger for in vitro particle opening we have analyzed viunalikevirus Det7. We found that a swiss army knife-like equipment of cell surface recognizing TSP enables myovirus Det7 to infect a broad host range. Thus, we could measure time-resolved *in vitro* DNA release in the presence of LPS preparations from different hosts. One of these hosts is *S. anatum*. We have isolated and structurally characterized Det7s TSP specific for *Salmonella anatum*. Our experiments emphasize that TSP-LPS recognition are a prerequisite for DNA release in the myovirus Det7. We found the size of LPS-aggregates in solution to be crucial for successful DNA-ejection *in vitro*, supporting the hypothesis that a membrane contact is sufficient to trigger particle opening.

P4 MSV, a novel archaeal lytic virus targeting *Methanosarcina* strains

KATRIN WEIDENBACH[1], HORST NEVE[2], OMER S. ALKHNBASHI[3], ANNE KUPCZOK[1], THORSTEN BAUERSACHS[4], ANDREAS THOLEY[5], RUTH A. SCHMITZ[1]
[1] Christian-Albrechts-University, Institute for General Microbiology, Kiel, Germany
[2] Max Rubner-Institut, Department of Microbiology and Biotechnology, Kiel, Germany
[3] Albert-Ludwigs-University, Institute of Informatics, Freiburg, Germany
[4] Christian-Albrechts-University, Institute of Geosciences, Department of Organic Geochemistry, Kiel, Germany
[5] Christian-Albrechts-University, Institute for Experimental Medicine, Department of Systematic Proteomics and Bioanalytics, Kiel, Germany
Corresponding author: kweidenbach@ifam.uni-kiel.de

A novel archaeal lytic virus targeting species of the genus *Methanosarcina* was isolated using *Methanosarcina mazei* strain Gö1 as host. Due to its spherical morphology the virus was designated *Methanosarcina spherical virus* (MSV). Molecular analysis demonstrated that MSV contains double stranded linear DNA with a genome size of 10,567 bp containing 22 open reading frames (ORFs) all oriented in the same direction. Functions were predicted for some of these ORFs, i. e. like DNA polymerase, ATPase, DNA-binding protein, as well as envelope (structural) proteins. MSV-derived spacers in CRISPR loci were detected in several published *Methanosarcina* draft genomes using bioinformatic tools, revealing the potential PAM motif (TTA/T). Transcription and expression of several predicted viral ORFs were validated by RT-PCR, PAGE analysis and mass spectrometry. Analysis of core lipids by APCI mass spectrometry showed that MSV and *M. mazei* both contain archaeol and glycerol dialkyl glycerol tetraether without cyclopentane moiety (GDGT-0). The MSV host range is limited to *Methanosarcina* strains growing as single cells (*M. mazei*, *M. bakeri* and *M. soligelidi*). In contrast, strains growing as sarcina-like aggregates were apparently protected from infection. Heterogeneity related to morphology phases in *M. mazei* cultures allowed acquisition of resistance to MSV after challenge by growing as sarcina-like aggregates. CRISPR/Cas mediated resistance was excluded since neither of the two CRISPR arrays showed MSV-derived spacer acquisition. Based on these findings, we propose that changing the morphology from single cells to sarcina-like aggregates upon rearrangement of the envelope structure prevents infection and subsequent lysis by MSV.

P5 Thermophilic bacteriophage *Aeribacillus pallidus* AP45 isolated from the Valley of Geysers from Kamchatka peninsula

VERA MOROZOVA[1], YULIYA KOZLOVA[1], OLGA BOKOVAYA[2], IGOR BABKIN[1], ANASTASIYA YUNUSOVA[3], NINA TIKUNOVA[1]

[1] Russian Academy of Sciences, Siberian Branch, Institute of Chemical Biology and Fundamental Medicine, Laboratory of Molecular Microbiology, Novosibirsk, Russia
[2] Novosibirsk State University, Faculty of Natural Sciences, Novosibirsk, Russia
[3] Russian Academy of Sciences, Siberian Branch, Institute of Chemical Biology and Fundamental Medicine, Group of Microscopy, Russia
Corresponding author: vera_morozova@ngs.ru

Bacterial communities of extremal biotopes were investigated more intensively recent years, than bacteriophages specific to bacteria from these habitats were studied.

Thermophilic bacteriophage AP45 and its host bacterium *Aeribacillus pallidus* CEMTC 656 were isolated from soil samples the Valley of Geysers from Kamchatka peninsula. According electron microscopy phage was identified as Siphoviridae family member. NGS-sequencing revealed phage genome with the length 51606bp. AP45 genome [KX965989] contained 74 putative ORFs, encoding recombinases and DNA-metabolism proteins, lysin, structural phage proteins, and 41 hypothetical ORFs, that had no similarity with GenBank Database sequences. Structural proteins of phage AP45 demonstrated similarity with sequences of thermophilic phage D6E [GU568037]. Non-structural proteins were similar to sequences, encoded by genomes of thermophilic bacteria belonging Bacillaceae family.

The optimal growth temperature for host strain *Aeribacillus pallidus* CEMTC 656 was 55-65 °C, and phage production was maximal at this temperature. Phage AP45 was thermostable at 55-65 °C and kept viable at 95 °C for more 2 hours.

Thereby, a novel thermophilic bacteriophage AP45, specific to *Aeribacillus pallidus* was investigated.

P6 Novel phage isolates infecting *Bacillus*

CYNTHIA MARIA CHIBANI, HEIKO LIESEGANG, ROBERT HERTEL
Georg-August University of Göttingen, Institute of Microbiology and Genetics, Genomic and Applied Microbiology, Germany
Corresponding author: cchiban@gwdg.de

Phages are considered as virus of bacteria. They infect their host and take over its metabolism for reproduction (lytic cycle), or silently integrate into its genome and become prophages (lysogenic cycle). Members of the genus *Bacillus* includes a range of versatile species of particular interest.

Genomic analysis of genomes of the genus revealed that prophages represent a major source of species specific genes and thus are contributing to the evolution of the strains. On the other hand, lytic phages of *Bacillus* can be used to suppress pathogenic strains within an infection, which is of significant importance due to the current antibiotic crisis. However, lysogenic prophages can switch to the lytic cycle within a growing culture and thereby erase most of the vegetative cells. Induced phage lysis is a disaster within biotechnological production systems.

Here we present new virus isolates infecting strains of *Bacillus subtilis*, *thuringiensis*, *amylolicifaciens* and *megaterium*. Morphological investigation using transmission electron microscopy revealed particles with a head tail morphology belonging to the Myoviridae, Siphoviridae and Podoviridae families. Genomes of all isolates were sequenced and their genomic content and structure were investigated.

The data we present here will help expand the knowledge of *Bacillus* related phages and in turn lead to a greater understanding of these organisms and their genomics, which promises better methods to either fight virulent *Bacilli* as well as to protect biotechnological production systems.

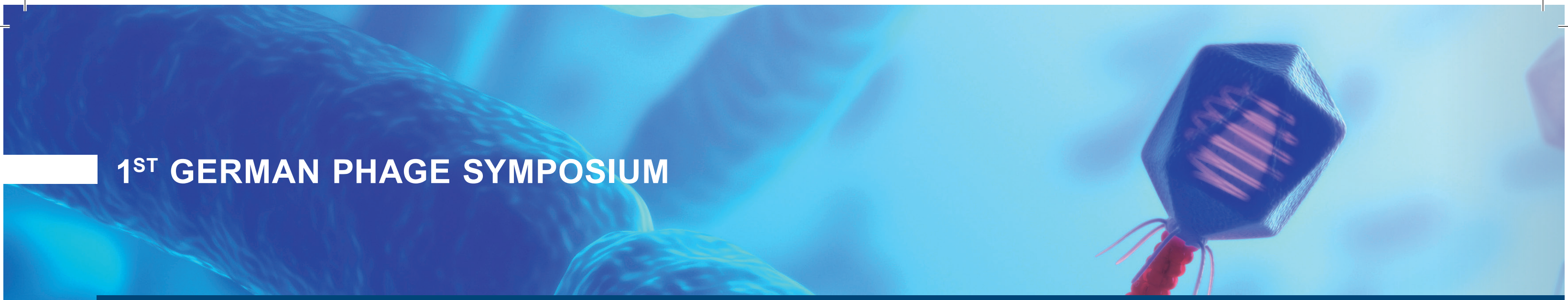

P7 Complete genome sequences of bacteriophages Sole and Sato expand the view of the genetic diversity found in the family Tectiviridae

ANNIKA GILLIS, LOUISE HOCK, JACQUES MAHILLON
Université Catholique de Louvain, Laboratory of Food and Environmental Microbiology, Louvain-la-Neuve, Belgium
Corresponding author: annika.gillis@uclouvain.be

Bacteriophages are an important repository of genetic diversity and source of unexplored genes. In the particular case of tectiviruses infecting the *Bacillus cereus* group, these bacteriophages represent part of the bacterial mobilome and they are, so far, the only ones able to behave as linear plasmids during their lysogenic cycle. Recently, several novel tectiviruses have been found infecting diverse strains belonging to this bacterial lineage (1, 2). Among them, partial DNA sequencing of tectiviruses Sole and Sato, isolated from *B. cereus sensu lato* and emetic *B. cereus* strains, respectively, uncovered a highly variable region potentially involved in lysogeny maintenance (1). Here, we analyze the complete genome sequences of bacteriophages Sole and Sato. The genome of Sole spans 14,445 bp with 28 putative CDSs, whereas the one of Sato has 14,852 bp comprising 31 putative CDSs. DNA sequence identity comparisons of Sato and Sole against *Bacillus* virus Bam35 (reference tectivirus) indicated 86 and 89 % sequence identity, respectively. Additionally, phylogenetic analyses and genome alignments suggested that both isolates represent novel tectivirus species. Hence, tectiviruses in Gram-positive bacteria have shown to be more diverse than those infecting Gram-negative bacteria, as the latter ones display a very high level of sequence identity. In an effort to assess the host range of Sole and Sato against emetic *B. cereus* and *Bacillus cytotoxicus*, two species involved in food poisoning outbreaks, 50 strains of each species devoid of tectiviral-like elements were tested. The results showed that Sole and Sato have a narrow host range, only infecting particular strains. Overall, genome sequencing and comparative analysis of tectiviruses Sole and Sato have expanded the view of the genomic diversity occurring in plasmidial prophages found in members of the *B. cereus* group.

1. Jalasvuori M, Palmu S, Gillis A, Kokko H, Mahillon J, Bamford JKH, Fornelos N. Identification of five novel tectiviruses in *Bacillus* strains: analysis of a highly variable region generating genetic diversity. Res Microbiol. 2013; 164:118-126.
2. Gillis A, Mahillon J. Prevalence, genetic diversity and host range of tectiviruses among members of the *Bacillus cereus* group. Appl Environ Microbiol. 2014; 80:4138-4152.

P8 Defining a core genome for the herpesvirales and elucidating their evolutionary relationship with the caudovirales

JUAN SEBASTIÁN ANDRADE MARTÍNEZ, ALEJANDRO REYES
Universidad de los Andes, Biological Sciences, Bogotá, Colombia
Corresponding author: js.andrade10@uniandes.edu.co

The order Herpesvirales encompasses a great variety of important and widely distributed human pathogens, including the Varicella-Zoster virus, Human Cytomegalovirus and Epstein-Barr virus. During the last decades, similarities in the viral cycle and the structure of some of their proteins with those of the tailed phages have brought speculation regarding the existence of an evolutionary relationship between the two clades. To evaluate such hypothesis, we used over 700 Herpesvirales and 2000 Caudovirales genomes downloaded from the NCBI genome and nucleotide databases, which were first de-replicated both at the nucleotide and amino acid level. Following this, they were screened for the presence/absence of clusters of orthologous viral proteins, and a dendrogram was constructed based on their compositional similarities. The results obtained strongly suggest that the Herpesvirales are indeed the closest viral order with eukaryote hosts to the Caudovirales, and allows putting forth hypotheses concerning the specific details of such relationship (i.e. whether they are sister clades or one stems from a minor clade within the other). Moreover, the identification of clusters that were abundant amongst the Herpesvirales made it possible to propose a Core Genome for the entire order, composed of 5 proteins, including the ATPase subunit of the DNA-packaging terminase, the only one with previously verified conservation in this clade. The fact that a phylogenetic tree constructed with sequences derived from the clusters associated to these proteins describes with fidelity the general architecture of the Herpesvirales strongly endorses the proposed Core Genome. Overall, this work simultaneously provides important results supporting the long-held hypothesis that the two orders are evolutionary related and contributes to the understanding of the evolutionary history of the Herpesvirales themselves.

P9 Takeover of *E. coli* by phage T5 is controlled by the DNase A1

OMBELINE ROSSIER[1], LEO ZANGELMI[1], MADALENA RENOUARD[2], PASCALE BOULANGER[2]

[1] University Paris-Sud, Institute for Integrated Biology of the Cell, Gif-sur-Yvette, France

[2] Centre National de la Recherche Scientifique, Institute for Integrated Biology of the Cell, Gif-sur-Yvette, France

Corresponding author: ombeline.rossier@u-psud.fr

Phage T5 infects *Escherichia coli* and injects its genome in an original two-step mechanism: in the First Step Transfer (FST), only 8 % of the phage DNA enters the host cell. The transfer pauses then for several minutes before DNA entry resumes to completion (Second Step Transfer, SST). The FST is accompanied by a very rapid and massive destruction of bacterial DNA (50 % decrease in labeled DNA within 4 min of infection). The identity of the phage T5 nuclease has remained elusive for sixty years, as none of the phage proteins encoded on the FST-DNA resemble known nucleases. However, A1, a gene carried by FST-DNA, appears to control host DNA degradation as well as the SST. In this study we investigated the role of the 62 kDa protein A1 in DNA degradation *in vitro* and in the bacterial cell.

In the C-terminal half of A1, we identified several motifs that are conserved in a large family of metallophosphatases including the DNA repair and recombination nucleases Mre11/SBcD/gp46. Purified A1 exhibited manganese-dependent DNase activity on linear or plasmid DNA *in vitro*, suggesting that A1 is a Mn-dependent nuclease. Using fluorescence microcopy of *E. coli* cells, we observed a rapid decrease in bacterial DNA staining with DAPI upon ectopic expression of A1. Moreover, we frequently saw the formation of a focus of fluorescence, suggesting a dramatic reorganization of the bacterial nucleoid. Mutations in putative catalytic amino-acid residues abolished nuclease activity *in vitro* as well as *in vivo*. Taken together, our results indicate that A1 is the long elusive early-encoded DNase of phage T5. Interestingly, T5 phage DNA is not modified and is sensitive to A1 *in vitro*. How the DNase activity of A1 is regulated to control the SST without digesting the T5 genome remains to be elucidated.

P10 A membrane-embedded molecular motor drives filamentous phage assembly

BELINDA LOH, MAX HAASE, ANDREAS KUHN, SEBASTIAN LEPTIHN
University of Hohenheim, Department of Microbiology & Molecular Biology, Stuttgart, Germany
Corresponding author: belinda.loh@uni-hohenheim.de

In contrast to lytic phages, filamentous phages are assembled in the inner membrane and secreted across the bacterial envelope without killing the host. The genome of filamentous phages codes for membrane proteins that are thought to facilitate the assembly and extrusion of the phage across the host cell wall. We characterized the M13 phage morphogenesis protein gp1 found in the inner membrane of the host using both, *in vivo* and *in vitro* methods. We identified Walker A and B motifs with a conserved lysine in the Walker A motif (K14), and a glutamic and aspartic acid in the Walker B motif (D88, E89) and showed that these residues are essential for assembly. For the *in vitro* characterization of the membrane protein complex, we expressed and purified gp1 and analyzed structure and function of this fascinating molecular motor. Here, we present the formation of a pore-like structure by the gene I products gp1 and the internal open reading frame gp11, a N-terminally truncate of gp1. The cytoplasmic domain of gp1, which is lacking in gp11, displays ATPase activity which is likely to be the driving force for the process of phage assembly.

P11 The *Ochrobactrum* genome, an important source of temperate phages

JENS ANDRE HAMMERL[1], CLAUDIA JAECKEL[1], STEFAN HERTWIG[1], HOLGER SCHOLZ[2], KARSTEN NOECKLER[1], JOCHEN REETZ[1]

[1] German Federal Institute for Risk Assessment, Biological Safety, Berlin, Germany

[2] Bundeswehr Institute of Microbiology, Germany

Corresponding author: jens-andre.hammerl@bfr.bund.de

Ochrobactrum and *Brucella* are closely related bacteria that, however, live in different habitats and diverge regarding their pathogenic properties. Only little is known about mobile genetic elements in these genera which might be important for survival and virulence. Previous studies on *Brucella* lysogeny indicated that active phages are rare in this genus. In this work temperate phages of *Ochrobactrum* were investigated for the first time. *In silico* analyses disclosed numerous prophages in published *Ochrobactrum* genomes. Induction experiments showed that *Ochrobactrum* prophages can be induced by various stress factors and that some strains released phage particles even under non-induced conditions. Sixty percent of lysates prepared from 125 strains revealed lytic activity. The host range and DNA similarities of 19 phages belonging to the families Myoviridae, Siphoviridae or Podoviridae were determined suggesting that they are highly diverse. Some phages showed relationship to the temperate *B. inopinata* phage BiPB01. The genomic sequences of the myovirus POA1180 (41,655 bp) and podovirus POI1126 (60,065 bp) were analysed. Phage POA1180 is very similar to a prophage recently identified in a *Brucella* strain isolated from an exotic frog. The POA1180 genome contains genes, which may confer resistance to chromate and the ability to take up sulfate. Phage POI1126 is related to podoviruses of *Sinorhizobium meliloti*, *Erwinia pyrifoliae* and *Burkholderia cenocepacia* and almost identical to an unnamed plasmid of the *O. intermedium* strain LMG 3301. Further experiments revealed that the POI1126 prophage indeed replicates as plasmid. The data demonstrate that mobile genetic elements are common in *Ochrobactrum*.

P12 A novel strategy for exploitation of host RNase E activity by a marine cyanophage

CLAUDIA STEGLICH, DAMIR STAZIC, IRENA PEKARSKI, MATTHIAS KOPF, DEBBIE LINDELL
University of Freiburg, Biology, Germany
Corresponding author: claudia.steglich@biologie.uni-freiburg.de

Previous studies have shown that infection of *Prochlorococcus* MED4 by the cyanophage P-SSP7 leads to increased transcript levels of host endoribonuclease (RNase) E. However, it has remained enigmatic if this is part of a host defence mechanism to degrade phage mRNA or if this single-strand (ss)RNA-specific RNase is utilized by the phage. Here we describe a hitherto unknown means through which this cyanophage increases expression of RNase E during phage infection and concomitantly protects its own RNA from degradation. We identified two functionally different RNase E mRNA variants, one of which is significantly induced during phage infection. This transcript lacks the 5'UTR, is considerably more stable than the other transcript, and is likely responsible for increased RNase E protein levels during infection. Furthermore, selective enrichment and *in vivo* analysis of double-stranded (ds)RNA during infection revealed that phage antisense (as)RNAs sequester complementary mRNAs to form dsRNAs, such that the phage protein-coding transcriptome is nearly completely covered by asRNAs. In contrast, the host protein-coding transcriptome is only partially covered by asRNAs. These data suggest that P-SSP7 orchestrates degradation of host RNA by increasing RNase E expression while masking its own transcriptome from RNase E degradation in dsRNA complexes. We propose that this combination of strategies contributes significantly to phage progeny production.

P13 *E. faecalis* uses quorum sensing to release prophages and differentially regulate important steps of host-pathogen interactions

LUIS FELIPE ROMERO-SAAVEDRA[1], FRIEDERIKE ROSSMANN[1], ANTONI P.A. HENDRICKX[2], AXEL HARTKE[3], KIRSTEN JUNG[4], JOHANNES HÜBNER[1]

[1] LMU Munich, Dr. von Hauner Children´s Hospital, Department of Pediatrics, Germany

[2] University Medical Center Utrecht, Department of Medical Microbiology, The Netherlands

[3] Université de Caen, Equipe Stress Virulence, Unité de Recherche Risques Microbiens (U2RM), France

[4] Ludwig-Maximilians Universität München, Center for Integrated Protein Science, Department of Biology I, Microbiology, Germany

Corresponding author: frsquim@gmail.com

The universal quorum sensing molecule autoinducer 2 (AI-2) is used by bacteria to regulate group dynamic behaviors. Here we show that AI-2 controls dose-dependently different steps during bacterial infection and release of prophages. An AI-2-negative mutant Δpfs did not produce endogenous AI-2 but responded to exogenous AI-2, forming strong biofilms at low (20 µM) AI-2 concentrations while dispersing biofilms at high (100 µM) AI-2 through the release of phages. At low exogenous AI-2, biofilm formation was twice as much compared to *E. faecalis* strains treated with high AI-2. At low AI-2, bacteria were strongly resistant to phagocytosis (39 % opsonic killing), while 67 % of the bacteria were killed when treated with high AI-2. TNF-α secretion of macrophages activated by bacteria exposed to low AI-2 was reduced by more than half compared to bacteria treated with high AI-2. Conversely, high AI-2 resulted in the induction of prophages which lead to biofilm dispersal. Additionally, a strong inflammatory response and strong binding to colonic epithelial cells were observed. Adhesion of bacteria to Caco-2 cells was significantly higher at high AI-2 compared to bacteria treated with low AI-2. We believe that AI-2 regulates expression of specific target genes (i.e. gls24-like protein at 20 µM and prophages at 100 µM) and allows enterococci to differentially express and distribute their virulence mechanisms through phages during attachment, colonization, and infection.

P14 High-frequency plasmid transduction to phage-insensitive recipient strains of *Staphylococcus aureus*

ROMAN PANTUCEK, IVANA MASLANOVA, SABINA STRIBNA, JIRI DOSKAR
Masaryk University, Department of Experimental Biology, Brno, Czech Republic
Corresponding author: pantucek@sci.muni.cz

Staphylococcus aureus is a major human and veterinary pathogen causing local and systemic infections. The evolution of this pathogen does not only take place in small steps through the accumulation of point mutations, but primarily through the uptake of foreign DNA by horizontal gene transfer. The transfer of DNA in a bacterial population is carrying through transformation, conjugation, and transduction. The primary driving forces in horizontal gene transfer in staphylococci seems to be the transduction mediated by bacteriophages. For a transduction to be effective, it is generally accepted that the recipient strain should be susceptible to the transducing phage.

We demonstrated that the plasmid DNAs are effectively transduced into the recipient *S. aureus* strains in spite of their insensitivity to the lytic action of the transducing phage, provided that these phages adsorb effectively to the bacterial cells. The tetracycline and penicillinase plasmids were transduced to insensitive laboratory and clinical strains by bacteriophages Φ29, Φ52A and Φ80α as well as by prophage Φ53 and naturally occurring prophages induced from donor lysogenic strains. The transduction frequencies from all experiments ranged from 1.6×10^{-6} to 1.2×10^{-11}. Comparable frequencies of transduction were achieved in both phage-sensitive and phage-insensitive recipient strains.

We have demonstrated that the restriction of DNA and lysogenic immunity which are responsible for insensitivity of cells to phages may not be a barrier to the transfer, maintenance and effective spread of plasmids to a wider range of potential recipients in the staphylococcal population even among different clonal clusters.

This work was supported by grants GP13-05069P, NT16-29916A and QJ1510216.

P15 Characterization of novel *Staphylococcus sciuri* bacteriophages participaing in interspecies plasmid transduction, and packaging mecA gene

IVANA MASLANOVA, MICHAL ZEMAN, ADELA INDRAKOVA, JIRI DOSKAR, ROMAN PANTUCEK
Masaryk University, Department of Experimental Biology, Brno, Czech Republic
Corresponding author: iva.maslanova@gmail.com

Staphylococcus sciuri is a mostly animal-associated bacterial species, but its clinical relevance for humans is increasing because it represents a reservoir for the mecA gene encoding methicillin-resistance in staphylococci. Although *S. sciuri* bacteriophages have not been characterized in details yet, it is accepted, that they may be involved in antimicrobial resistance gene transfer in staphylococcal population.

Here we present two novel *S. sciuri* temperate phages of Siphoviridae family designated Φ575 and Φ879. Their genomes and genomes of their host strains were sequenced on the Ion Torrent™ platform. Genomes of the phages are 42,203 and 41,491 bp long and encode 58 and 55 ORFs, respectively, arranged in functional modules. Their head-tail morphogenesis modules are similar to those of *Staphylococcus aureus* Φ13-like serogroup F phages, suggesting their common evolutionary origin.

Genes that can enhance the virulence of the bacterial host were identified in both phage genomes. Phage Φ575 harbours genes for staphylokinase and phospholipase that might enhance the virulence of the bacterial hosts. In addition, both of the phages package a homologue of mecA gene that is a prerequisite for its lateral transfer, which was proven by using real-time PCR. The packaging frequency of the mecA part of SCCmec was about three orders of magnitude lower than that of the plasmid-borne aadD gene from tetracycline and aminoglycoside pSTS7-like resistance plasmids. Phage Φ879 transduces tetracycline and aminoglycoside pSTS7-like resistance plasmids from its host to *S. sciuri* and to *S. aureus* with a frequency of about 10^{-11}. Furthermore, both of the phages efficiently adsorb on the cells of different staphylococcal species supports the speculation of the interspecies horizontal transfer of mobile elements or its parts by phages.

This work was supported by grant s 16-29916A and QJ1510216.

P16 Isolation and characterization of bacteriophages from *Bacillus anthracis*

CARINA ROHMER[1], WOLFGANG BEYER[2]

[1] University of Tübingen, Interfaculty Institute of Microbiology and Infection Medicine, Germany

[2] University of Hohenheim, Institute of Animal Science, Department of Livestock Infectiology and Environmental Hygiene, Stuttgart, Germany

Corresponding author: Carina.Rohmer@med.uni-tuebingen.de

Bacillus (B.) anthracis, the causing agent of anthrax mainly infects herbivores but is also a serious threat for humans due to its potential as a biological weapon [1]. Investigations of environmental and animal *B. anthracis* isolates indicate that at least 20 % are stable infected with one or more bacteriophages [2]. The aim of this work was the isolation and characterization of bacteriophages from *B. anthracis* isolated from animals which died from anthrax and environmental strains as well which were collected in South Africa (SA) and Namibia (AF). Several virulent isolates of the *B. anthracis* strain collection were tested for spontaneous release of bacteriophages or by induction. For phenotypic analysis phages were stably introduced in an avirulent recipient to form lysogens. Phage morphology was determined via electron microscopy. Specificity test with several species of the *B. cereus* group was performed for identifying the bacteriophage host range. Further experiments were carried out for investigation of any influence of the phage onto the biology of *B. anthracis* - like biofilm test and sporulation assay. So far 27 bacteriophage lysates were collected. All phages belong to the order of Caudovirales, representing either Myoviruses or Siphoviruses. Specificity tests resulted in different infection capabilities for 17 species tested. 75 % of the phages enabled biofilm formation in the experimental lysogens. 7 bacteriophages seem to have an influence on sporulation capacity of the tested lysogens. Additionally, phenotypic changes in colony morphology were observed which were attributable to stable phage infection.

In summary, our results indicate that i) bacteriophages are present in *B. anthracis* isolated directly from the blood of dead animals and ii) such phages may have an impact on the live cycle and survival strategies of its host by active lysogeny [3,4].

1. WHO (2008). Anthrax in humans and animals. 4th edition.

2. Schuch R, Fischetti VA. The secret life of the Anthrax agent *Bacillus anthracis*: Bacteriophage-mediated ecological adaptions. PLoS ONE. 2009;4: e6532.

3. Feiner R, Argov T, Rabinovich L, Sigal N, Borovok I, Herskovits AA. A new perspective on lysogeny: prophages as active regulatory switches of bacteria. Nat Rev Microbiol. 2015;13:641-650.

4. Argov T, Azulay G, Pasechnek A, Stadnyuk O, Ran-Sapir S, Borovok I, Sigal N, Herskovits AA. Temperate bacteriophages as regulators of host behavior. Current Opinion in Microbiology. 2017;38:81–87.

P17 Staphylococcal factors influencing the biology of Sa3-bacteriophages

CARINA ROHMER, CHRISTIANE WOLZ
University of Tübingen, Interfaculty Institute of Microbiology and Infection Medicine, Germany
Corresponding author: Carina.Rohmer@med.uni-tuebingen.de

Staphylococcus aureus possesses a set of virulence factors necessary for infection of the human host some of which are encoded on bacteriophages. Sa3-phages integrate into the *hlb*-gene therefore leading to loss of β-hemolysin production but provide additional virulence genes to their host bacterium. About 90 % of all *S. aureus* strains of human origin carry these *hlb*-converting phages, whereas animal strains usually are devoid of them. We aim is to investigate how the bacterial host interferes with the biology of these Sa3-phages, especially Φ13. Phage cured derivatives of *S. aureus* strain 8325 (8325-4, Clonal complex 8) and of the Methicillin-resistant *S. aureus* strain MW2 (MW2c, Clonal complex 1) were lysogenized with Sa3-bacteriophage Φ13 carrying a kanamycin resistance cassette (Φ13Kana). Phage induction was quantified using phage titering and qPCR to quantify integrated and excised phage copies after induction with subinhibitory concentrations of Mitomycin C. *In vitro* phage transfer was performed by co-culturing lysogens with phage free recipients. Phage induction was significantly higher in MW2c compared to 8325-4 background. Phage transfer was also significantly higher in MW2c background and occurred without the need of induction by Mitomycin C. Correct phage integration into the *hlb*-gene was proven by Multipex-PCR and pulsed-field gel electrophoresis (PFGE). PFGE analysis followed by Southern hybridization revealed concatemer formation in the new lysogens. In summary, there are strain specific differences regarding the induction and lysogenization rate of Φ13.

P18 Role of phages in *Shewanella oneidensis* MR-1 biofilm formation

MAXIMILIAN KREIENBAUM, LUCAS BINNENKADE, KAI THORMANN
Justus-Liebig University Gießen, Microbiology and Molecular Biology, Germany
Corresponding author: maximilian.kreienbaum@mikro.bio.uni-giessen.de

Prophages, the dormant form of temperate phages, are found ubiquitously in bacterial genomes. While it was previously believed that induction of these are solely detrimental to bacteria, more recent studies have demonstrated it to be a possible competitive advantage under certain conditions, aiding the bacterial populations in colonization of surfaces and increasing the pathogenicity towards their host.

Shewanella oneidensis MR-1 harbors three prophages: λSo, MuSoI and MuSoII with MuSoI being cryptic. In this species, the prophages are required for proper biofilm formation of *S. oneidensis* MR-1 by mediating cell lysis and consequential release of extracellular DNA as crucial structural biofilm matrix component. Further experiments on λSo demonstrated that induction and subsequent cell lysis occur as a consequence of cell stress which is likely induced by increased iron acquisition by a cellular subpopulation during early biofilm formation.

In order to better understand timing and mechanism of phage production, we introduced an additional copy of the gene encoding the major capsid protein fused to yfp into the prophage chromosome, yielding highly fluorescent but still active phage particles. By this, we are able to observe synthesis of the phage particles starting with their construction within the host cell, and, subsequent to cell lysis, to track the phage particles' spreading through the surrounding to finally engage new host. Current experiments use fluorescently labeled λSo as a tool to further elucidate the interaction between bacteriophages and their hosts in biofilm formation.

P19 When more is not better: A generalist predator dampens the impact of a phage and a predatory bacterium on their preferred prey and enables coexistence

ANTONIS CHATZINOTAS[1], JULIA JOHNKE[1], MAAYAN BARON[2], MARINA DE LEEUW[2], ARIEL KUSHMARO[2], EDOUARD JURKEVITCH[3], HAUKE HARMS[1]

[1] Helmholtz Centre for Environmental Research – UFZ, Environmental Microbiology, Leipzig, Germany

[2] Ben-Gurion University of the Negev, Beer-Sheva, Israel

[3] The Hebrew University of Jerusalem, Israel

Corresponding author: antonis.chatzinotas@ufz.de

The therapeutic use of bacteriophages to combat bacterial pathogens in an agricultural, biotechnological or even clinical context requires knowledge about the ecological and evolutionary consequences of microbial interactions in complex systems. Natural, engineered and host-related habitats usually harbour multiple micro-predator and prey species whose direct and indirect interactions are mostly understudied. In particular, the combined effects of different predators and their varying prey preference on prey removal are not known. For instance, one may assume that combining a phage with other potentially complementary predators would result in an increased reduction of target prey species.

To better understand the effect of interspecies interactions, combinations of micro-predators, i.e. protists (generalists), predatory bacteria (semi-specialists), and phages (specialists), and bacterial prey were tracked over a 72-hour period in miniature bioreactors. While specialist predators, e.g. the phage, alone drove their preferred prey to extinction, the inclusion of a generalist resulted in similar losses among the prey species. Most importantly, presence of a generalist predator basically neutralized the impact of the phage and even enabled coexistence of all predators and prey.

The appearance of resistant prey strains and subsequent coevolution of the specialist predators implies that multitrophic communities are able to persist and stabilize themselves. Interestingly, coevolution was observed for the phage (and the predatory bacterium) in the absence of additional predators or prey species indicating that competition between predators might influence coevolution dynamics.

P20 Spatial distribution and dynamics of viral communities along a river as revealed by metagenomic

RENÉ KALLIES, CHRISTIAN GRIEBLER
Helmholtz Centre for Environmental Research – UFZ, Leipzig, Germany
Corresponding author: rene.kallies@ufz.de

Rivers are vital aquatic habitats and networks in the terrestrial environment acting as drainage channels and carrying carbon and nutrients. Due to the increasing impact from climate change and human activities many rivers have been changing.

These impacts influence the population dynamics and diversity of microorganisms. These microorganisms code important for many ecosystem functions, such as nutrient cycling, mineralization of organic matter or the purification of the habitat. However, these microorganisms are strongly affected by their “micro-predators”, such as viruses. Prokaryotic viruses are a key factor for bacterial mortality, drive diversification and evolution of their bacterial hosts through gene transfer and are, via lysis of microbes, important contributors to the recycling of carbon and nutrients. Compared to marine ecosystems, the composition and impact of viral communities in freshwater environments still remains largely unexplored.

In this study, we aimed to reveal the spatial dynamics of viral communities in the Holtemme River along a 40 km long section that starts in the Harz Mountains and discharges in the Bode River. Water was collected from thirteen sampling sites reflecting specific influences, such as arriving tributaries, outlet of waste water treatment plants (WWTP), areas of intense agriculture and restored river sections. Samples were analysed for the total number of virus-like particles (VLP) and viral metagenomics.

The number of VLPs increased over the course of the river exhibiting distinct peaks at areas impacted by outlets of WWTP, ranging between $2x10^5$ and 10^8 VLP/ml. Metagenome data revealed a high number of unknown sequences. Viral communities were dominated by unclassified viruses and within the virus sequences affiliated, members of Caudovirales and Microviridae. Few sequences belonged to plant-, protist- and insect-infecting viruses. Highest diversity of viral sequences was found in urban areas. K-mer analysis showed no correlation of virus community structure and course of the river.

P21 Estimation of ecological coherences between phages and metal resistant *Streptomyces* from metal contaminated sites by genomics

THOMAS KRAUßE[1], ANNEKATRIN VOIT[1], KARIN MARTIN[2], ERIKA KOTHE[1]

[1] Friedrich-Schiller-University Jena, Institute of Microbiology - Microbial Communication, Jena, Germany

[2] Leibniz Institute for Natural Product Research and Infection Biology - Hans Knöll Institute, Bio Pilot Plant, Jena, Germany

Corresponding author: thomas.krausse@uni-jena.de

The continual increase in heavy metal contaminated soils asks for concepts for handling and remediation. In this framework, microbially assisted phytoremediation has been proposed to alter metal bioavailability which can be applied to reduce concentrations and toxic effects. A major group of bacteria involved in this processes are aerobic, gram-positive and filamentous *Streptomyces* species, a dominant group of soil bacteria specifically enriched at metal containing sites. Their growth, distribution and activity is influenced by specific bacteriophages, making investigations of major ecological influences of phages on the diversity of soil microbial populations necessary to evaluate the impact of streptomycetes on bioremediation approaches. In addition, phages are the largest genetic pool on earth, and therefore can be used as tools for genetic and biotechnological processes, especially since they are naturally involved in horizontal gene transfer. In consequence, streptophages (infecting species of *Streptomyces*) may be active in distributing important metal resistance genes such as methallothioneins, chelators (e.g. siderophores) or efflux transporters among *Streptomyces* species. To better understand the processes active between members of *Streptomyces* and their specific phages, the polyvalent phage S7, isolated from compost, the narrow host range phage S3 from garden soil, and phage L44 from forest soil were sequenced in addition to two metal resistant *Streptomyces* strains E13 and P16B-1. In addition, morphological investigations of the life cycle and phage particles via TEM allowed a taxonomic classification of the streptophages. Structural genes have been identified by LC-MS.

Genetic manipulation of *Streptomyces*, e.g. with a streptophage-*Streptomyces* transduction system, is desired, opening up possibilities to investigate the ecological role in metal resistance of streptomycetes. Our investigations are adding to the understanding of the role of phages for soil microbiology in general, as well as with specific impact for metal contaminated site ecology, bioremediation, and bioactive natural compounds.

P22 Closing the gap: Phage research at the Helmholtz Centre for Environmental Research – UFZ

ANTONIS CHATZINOTAS, BÄRBEL KIESEL, RENÉ KALLIES
Helmholtz Centre for Environmental Research – UFZ, Environmental Microbiology, Leipzig, Germany
Corresponding author: antonis.chatzinotas@ufz.de

The relevance of phages for their hosts' diversity, ecology and evolution are now widely recognized and accepted. In addition, interest in the use of phages to control potentially pathogenic (or in some other way undesired) bacterial hosts has again gained momentum not only in a medical but also in an environmental and biotechnological context. Most progress in the field of phage ecology and diversity has been made in marine systems, while terrestrial, freshwater or host-associated systems have been largely neglected so far.

This "info-poster" aims to present the activities of our group at the UFZ-Department of Environmental Microbiology in Leipzig to close this particular gap. We will briefly summarize several research activities and third-party funded projects which seek to reveal the ecological and evolutionary consequences of phage-bacteria interactions on bacterial communities and their functions. Experimental setups range from controlled lab-systems to field sites, from short-term studies on the arms race between phages and their hosts to studies addressing the spatio-temporal dynamics of phages.

The method tool box available in our group covers classical as well as high-throughput sequencing approaches: these include the isolation and TEM-description of phages and their hosts from diverse environments as well as the omic-methods which provide insight into the genomes of isolates or the metagenomes (i.e. viromes) in distinct habitats. This brief overview will be concluded with an outlook on planned activities focusing on host-associated phages and viromes.

P23 Broad host-range T5-like *Yersinia enterocolitica* phage phiR2-01

MARIA I. PAJUNEN[1], LOTTA J. HAPPONEN[2], JIN WOO JUN[1], MIKAEL SKURNIK[1]
[1] University of Helsinki, Department of Bacteriology and Immunology, Finland
[2] Lund University, Division of Infection Medicine, Lund, Sweden
Corresponding author: maria.pajunen@helsinki.fi

Here we report the complete genome sequence and morphological characterization of the T5-like siphovirus vB_YenP_phiR2-01 (in short phiR2-01) infecting *Yersinia enterocolitica*, a zoonotic food-borne pathogen causing yersiniosis in humans and animals. Bacteriophage phiR2-01 encodes for 152 open reading frames (ORFs) and 19 tRNA-molecules on its 122,696 bp-long double-stranded DNA genome including 9,901 bp terminal repeats. A total of 115 of the ORFs are similar to genes of the well-characterized bacteriophage T5, including all genes involved in phage morphogenesis. The genome of phiR2-01 is mostly syntenic to that of T5, and the major differences between the genomes reside in areas with genes encoding for hypothetical phiR2-01 proteins. Other phages similar to phiR2-01 are the *Salmonella enterica* serovar Typhimurium phage Stitch, the *Escherichia* phage EPS7, the *Salmonella* phage Shivani, the *Escherichia* phage vB_EcoS_FFH1, the *Enterobacteria* phage DT57C and DT571/2, the *Escherichia* phage AKFV33, and the *Salmonella* phage SPC35. The host range of the phage is broad among *Y. enterocolitica* and it infects strains of both pathogenic and non-pathogenic serotypes. It does not infect strains of *Y. pseudotuberculosis*, *Y. nurmii*, *Y. pekkanenii*, *Y. mollaretii*, *Y. frederiksenii*, *Y. intermedia*, *Y. ruckeri*, *Y. bercovieri*, *Y. kristenseni* (except one strain), and *Y. aleksiciae*. The phage also failed to infect *Escherichia coli* and *Shigella* strains. Isolation of phage-resistant mutants from a transposon-insertion library revealed that the phage receptor is the BtuB protein.

P24 Efficacy of bacteriophage-antibiotic combinations against MRSA biofilm *in vitro* evaluated by isothermal microcalorimetry

TAMTA TKHILAISHVILI, ANDREJ TRAMPUZ, MARIAGRAZIA DI LUCA
Charité – Universitätsmedizin Berlin, Berlin-Brandenburg Centre for Regenerative Therapies, Germany
Corresponding author: tamta.tkhilaishvili@charite.de

Background: The potential application of phage therapy for the control of bacterial biofilms has received increasing attention as the resistance to conventional antibiotics reduces treatment options. This study was designed to assess the synergistic antimicrobial effect of phages combined with antibiotics against MRSA biofilm in real-time using a highly sensitive method measuring growth-related heat production.
Material/methods: *Staphylococcus aureus* specific phage Sb-1 with rifampin, fosfomycin, vancomycin and daptomycin were tested against MRSA (ATCC 43300). Biofilm was formed on porous glass beads and incubated for 24 h at 37 °C in brain-heart infusion. Then beads were washed in sterile saline trice and incubated in fresh medium containing sub-inhibitory bacteriophages concentrations for 24 h. Antibiotics were exposed simultaneously together with the phage or after 24 h phage treatment. After antimicrobial challenge, the beads where rinsed thrice and placed in microcalorimetric ampoules. Viable bacteria in biofilm were detected by measuring heat production at 37 °C for 48 h.
Results: The minimum biofilm bactericidal concentration for all antimicrobials tested alone were above reachable concentrations in clinical practice and ranged from 128 to 4096 µg/ml. Synergistic activity against biofilm MRSA was observed when bacteria were exposed to antibiotics only after 24h phage treatment. Bacterial growth-related heat was decreased by subinhibitory concentrations of antibiotics in combination with all titers of Sb-1 (10^5, 10^4, 10^3, 10^2 pfu/ml), but complete inhibition of heat production was showed with 10^5 and 10^4 pfu/ml titers. High synergistic activities were observed with all the tested combinations.
Conclusions: These findings indicate that synergy of antibiotics and bacteriophage Sb-1 is significantly more effective than the current treatment of MRSA biofilm based on conventional antibiotic alone. Therefore, bacteriophage-antibiotic synergies have potential for improving the treatment of implant-associated infections.

P25 The lactococcal P335 phage TP901-1 requires the host-encoded Gtf41, a putative glycosyltransferase, for early stages of infection

ANDREA CAROLINA ERAZO GARZON[1], JENNIFER MAHONY[1], FRANCESCA BOTTACINI[1], FINN K. VOGENSEN[2], HORST NEVE[3], KNUT J HELLER[3], CHRISTIAN CAMBILLAU[4], DOUWE VAN SINDEREN[5]

[1] University College Cork, School of Microbiology, Ireland
[2] University of Copenhagen, Dep. of Food Science, Microbiology and Fermentation, Denmark
[3] Max Rubner-Institut, Department of Microbiology and Biotechnology, Kiel, Germany
[4] Aix-Marseille Université, Architecture et Fonction des Macromolécules Biologiques, France
[5] University College Cork, APC Microbiome Institute, Ireland
Corresponding author: 114224203@umail.ucc.ie

Lactococcal phages are ubiquitous in the dairy environment. The most widely used bacterial species in the dairy fermentation industry is *Lactococcus lactis* which is susceptible to infection by lactococcal phages. Phage infection of lactic acid bacteria (LAB) starter and adjunct strains is a major concern in the dairy industry as it negatively impacts on the final product quality and production regimes with significant associated economic implications. Therefore, an improved understanding of the interactions of these problematic phages and their hosts is necessary for the construction of robust starter cultures and/or the selection of rational strain rotations/blends. Three *Lactococcus lactis subsp. cremoris 3107* derivatives known as *E119*, *E121* and *E126* had previously been shown to have obtained resistance against the lactococcal P335 phage TP901-1, while remaining sensitive to infection by phage phiLC3. The genomes of the parent strain and the phage-resistant mutants were sequenced and compared resulting in the identification of several mutations that may have caused TP901-1 resistance. Subsequent complementation assays showed that the introduction of a plasmid harbouring the gtf41 gene, which encodes a predicted oligosaccharide transferase, from *L. lactis 3107* wild-type, which is mutated in *E119* and *E126* mutants, restored the phage sensitivity phenotype of both mutants. Furthermore, the lysogenization frequency increased from 10^{-3} to 10^{-4} suggesting improved DNA injection efficiency. Moreover, silencing of gtf41 by an anti-sense mRNA interference strategy was shown to interfere with infection by phage TP901-1 in strain 3107. Therefore, gtf41 is involved in the early stages of infection of the lactococcal P335 phage TP901-1.

P26 Insights into bacteriophage – *Salmonella* coevolution

MICHELA GAMBINO, JONAS BYLOV HEDEGAARD ANDERSEN, Y. EMRE GENCAY, LONE BRØNSTED
University of Copenhagen, Department of Veterinary and Animal Sciences, Denmark
Corresponding author: mgambino@sund.ku.dk

The use of phages as alternatives to traditional antibiotics is becoming increasingly attractive, due to the rise and spread of antibiotic resistant bacteria. Nevertheless, bacteria can develop resistance to phages too. These adaptation strategies are much less known and need to be investigated to be able to use phages to control pathogens in human and veterinary medicine as well as in food production.

In this project, we studied the coevolution of an environmental phage, S118, dependent on the O-polysaccharide as bacterial receptor, and his host, the Gram-negative foodborne pathogenic bacterium, *Salmonella typhimurium* (*Salmonella enterica* serovar *Typhimurium LT2* prophage cured). Salmonella resistance mechanisms were investigated by transferring coevolved phages and bacteria every 24 h, for 9 days. The experiment was performed as 3 biological replicates by inoculating 3 microcosms with 10^6 cfu/ml *Salmonella* cells and 10^8 pfu/ml phages. For each transfer, 5 different colonies were isolated from the coevolved *Salmonella* populations and their resistance were tested against phages from each transfer.

Our preliminary results showed: I) the emergence of resistance to S118 phage already after 24 h; II) transfer after transfer, the increasing rise of *Salmonella* colonies completely resistant to all phages both from past, present and future transfers; III) the change of plaque morphology after 24 h in the evolution experiments and after 48 h in the coevolution; IV) the reduced infectivity (but not the extinction!) of evolved and coevolved S118 against coevolved *Salmonella*.
Further insights about the evolved phages are coming from the screening of a collection of *Salmonella typhimurium* mutant strains in the main receptors (O-PS, flagellum, vitamin B membrane transporter). Currently we investigate the resistance mechanisms *Salmonella* evolved in so short time by performing adsorption assays, studying the resistance to a collection of other *Salmonella* phages and whole genome sequencing.

P27 Bacteriophage PMBT2 with lytic activity against multidrug-resistant *Enterococcus faecalis* strains harbors a pseudo-metallo-ß-lactamase

SABRINA KOBERG, **ERIK BRINKS**, **HORST NEVE**, **CHARLES FRANZ**
Max Rubner-Institut, Department of Microbiology and Biotechnology, Kiel, Germany
Corresponding author: sabrina.koberg@mri.bund.de

A putative metallo-ß-lactamase (MBL) gene was identified in the genome of the virulent *Enterococcus faecalis* phage PMBT2 isolated from sewage. Homologous genes were reported to be present in other *Enterococcus* phages, but not in bacteria genomes. When cloned and expressed in *Escherichia coli*, *Lactococcus lactis* and *Streptococcus thermophilus* using two different expression vectors, the gene product did not mediate any resistance towards tested ß-lactam antibiotics and was consequently designated as a pseudo-metallo-ß-lactamase. Phage PMBT2 showed morphological characteristics of the Siphoviridae family and specifically lysed multidrug resistant *E. faecalis* strains. Its dsDNA genome comprised 41 489 bp with a mol% G+C content of 34.7, encoding 68 putative open reading frames (ORFs) and no tRNAs. It showed closest (90 %) sequence similarity to that of *E. faecalis* phage EfaCPT1. After verification that the phage genome did not encode any toxin genes, PMBT2 fulfilled the prerequisites to be used as a therapeutic agent for the biocontrol of multidrug-resistant *E. faecalis*.

P28 Effective phages infecting Livestock-Associated Methicillin-Resistant *Staphylococcus aureus* CC398 strains are found from pig faeces and environments

ANNAMARI HEIKINHEIMO
University of Helsinki, Faculty of Veterinary Medicine/Dept of Food Hygiene and Environmental Health, Finland
Corresponding author: annamari.heikinheimo@helsinki.fi

Livestock-associated methicillin resistant *Staphylococcuss aureus* (LA-MRSA) representing clonal complex CC398 has emerged worldwide during the last decade and can be found at especially in pigs. In Finland, carriage of LA-MRSA CC398 has become more common in pigs as well as in persons with occupational contacts to pigs; especially among farmers and veterinarians. LA-MRSA CC398 was found in 2.9 % of human MRSA infections in Finland in 2016.

Phage therapy is a potential treatment for *S. aureus* infections and a reduction of MRSA CC398 in livestock might be achieved by application of virulent phages. We have previously isolated three *S. aureus*–specific phages (fPf-Sau02, fPf-Sau03, and fPf-Sau04) from pig faeces from two Finnish farms. We have shown that these phages were not able to infect any of the human clinical *S. aureus* strains tested, but rather infected 80 % of the tested LA-MRSA strains. However, clonal complexes, spa types, resistance phenotypes as well as virulence and resistance gene profiles of those strains were not known.
In the present study, a collection of MRSA strains (N=16) originating from Finnish slaughter pigs were screened for their genotypes as well as resistance and virulence phenotypes. Altogether four (25 %) methicillin susceptible (MSSA) and 12 (75 %) methicillin resistant (MRSA) strains were detected. Of MRSA strains, a total of 8 (75 %) belonged to clonal complex CC398. The preliminary results showed that phages fPf-Sau02, fPf-Sau03, and fPf-Sau04 infected 7 out of 8 (88 %) of LA-MRSA strains belonging to CC398 and only 1 (13 %) of those strains belonging to other clonal complex than CC398. Two phages fPf-Sau02 and fPf-Sau03 possessed broader phage profile against CC398 strains than fPf-Sau04. These preliminary findings suggest wide variation of different MSSA and MRSA strains in Finnish slaughter pigs and that effective phages infecting these strains may be found from pig faeces and environments.

P29 A novel phage phiE72: an alternative therapeutic against *Staphylococcus epidermidis* infection and a potential research tool

XIN DU[1], VOLKER WINSTEL[2], PETRA KÜHNER[1], YORK-DIETER STIERHOF[3], ANDREAS PESCHEL[1]
[1] University of Tübingen, Infection Biology, Germany
[2] University of Chicago, Cummings Life Science Center, Chicago, USA
[3] University of Tübingen, Center for Plant Molecular Biology, Germany
Corresponding author: xin.du@student.uni-tuebingen.de

Staphylococcus epidermidis is one of the most common pathogens causing various types of nosocomial infections in hospitals, mainly by forming biofilms on medical devices. Nowadays, the situation of increasing number of *S. epidermidis* developed resistance to antibiotics is calling for alternative therapeutics. Besides, a novel research tool is also expected since study of the pathogenicity of *S. epidermidis* is limited due to genetic manipulation failure caused by strong genetic barrier mechanisms, especially the clinical ones. Recently, we isolated a new bacteriophage named phiE72 from a *S. epidermidis* strain in an infected tooth of a clinical patient. Electron microscopy revealed characteristics as bacteriophages of the Siphoviridae family. Phage infection assay using different bacterial species showed that phiE72 has a narrow host range and is specific to *S. epidermidis*. It showed a more drastical decrease of turbidity of bacterial host cell culture even compared to the widely studied antibiotic reagent member lytic polyvalent phage phiK. PhiE72 remained stable at pH values between 5.0 and 8.0 and up to the temperature of 60 °C. PhiE72 also showed tolerance to chloroform. The fast and strong lyse property, and specificity for *S. epidermidis* indicates the novel phage phiE72 an attractive candidate for phage therapy or as a biofilm eradication agent against *S. epidermidis*. Moreover, phiE72 can transduce plasmid DNA efficiently even to strains refractory to electroporation. Therefore, phiE72 might also become a valuable research tool for plasmid transduction for S. *epidermidis* strains, which are often difficult to transform.

P30 vB_SauM-fRuSau02, a Twort-like phage used for phage therapy

SAIJA KILJUNEN[1], KATARZYNA LESKINEN[1], HENNI TUOMALA[1], ANU WICKLUND[1], JENNI HORSMA-HEIKKINEN[1], PENTTI KUUSELA[2], MIKAEL SKURNIK[1]

[1] University of Helsinki, Research Programs Unit, Finland

[2] Helsinki University Hospital, Division of Clinical Microbiology, HUSLAB, Finland

Corresponding author: saija.kiljunen@helsinki.fi

Staphylococcus aureus is a commensal and pathogenic bacterium that causes infections in humans and animals. It is a major cause of nosocomial infections worldwide and WHO currently regards antibiotic-resistant *Staphylococcus aureus* as one of the major concerns for public health. Due to increasing prevalence of multi-drug resistance, alternative ways to eradicate the pathogen are necessary. In this respect, polyvalent Staphylococcal myoviruses have been demonstrated to be excellent candidates for phage therapy. Here we present the characterization of bacteriophage vB_SauM-fRuSau02 (fRuSau02) that was isolated from a commercial *Staphylococcus* Bacteriophage cocktail produced by Microgen. The genomic analysis revealed that fRuSau02 is very closely related to phage MSA6, possesses a large genome (148,464 bp) with typical modular organization and a low G+C (30.22 %) content and therefore can be classified as a new virus among the genus Twortlikevirus. The genome contains 236 predicted genes, four of which were interrupted by insertion sequences. Altogether 78 different structural and virion-associated proteins were identified from purified phage particles by LC-MS/MS. The host range of fRuSau02 was tested with 135 strains, including 51 and 54 *S. aureus* isolates from humans and pigs, respectively, and 30 coagulase-negative *Staphylococcus* strains of human origin. All clinical *S. aureus* strains were at least moderately sensitive to the phage, in contrast to only 39 % of the pig strains being infected. Also some strains of *S. intermedius*, *S. lugdunensis*, *S. epidermidis*, *S. haemolyticus*, *S. saprophyticus* and *S. pseudointer* were infected. We conclude that fRuSau02, a successful phage therapy agent in Russia, can serve as an alternative to antibiotic therapies against *S. aureus*.

P31 Use of bacteriophages for preventing methicillin resistant *Staphylococcus aureus* biofilm formation

MARIAGRAZIA DI LUCA, TAMTA TKHILAISHVILI, ANN-BRIT KLATT, ANDREJ TRAMPUZ
Charité – Universitätsmedizin Berlin, Germany
Corresponding author: mariagrazia.di-luca@charite.de

Biofilms on both soft tissues and implants are causing persistent infections that are often difficult to eradicate with the current antibiotic therapy. Bacteriophages have emerged as a promising alternative to antibiotic because of their high selectivity and rapid bactericidal activity. Here, we investigated the activity of lytic bacteriophages (Sb-1 and Pyo-bacteriophage) against planktonic and in preventing biofilm formation of methicillin resistant *Staphylococcus aureus* (MRSA).

Plaque-assay was used to test Sb-1 and Pyo-bacteriophage against MRSA ATCC 43300 and different clinical strains of *S. aureus*. Increasing titers of both phages (10^2 to 10^6 PFU/ml) were assayed against planktonic MRSA (10^6 CFU/ml) and in preventing biofilm formation (on porous glass beads) in BHI using isothermal microcalorimetry. After microcalorimetry experiments, beads were sonicated and pleated into BHI agar for colony counting. Confocal laser scanning microscopy was used to evaluate the presence of MRSA biofilm on glass bottom petri dishes after co-incubation with phages.

Both Sb-1 and Pyo-phage exhibited lytic activity against approximately the 75% *S. aureus* clinical strains. Microcalorimetry measurement showed that the highest titers of Sb-1 and Pyo-phage (10^6 and 105 PFU/ml, respectively) were able to inhibit planktonic growth of susceptible bacterial strains. In biofilm prevention experiments, the heat production was completely abolished in the presence of 10^4 Plaque-Forming-Units (PFUs)/ml of bacteriophages, with 10^2 PFUs/ml already inducing 50 % reduction of MRSA heat production. Confocal laser scanning microscopy analysis did not reveal the presence of any adherent cells on the surface of the glass bottom petri dish.

Sb-1 and Pyo-phage are active against *S. aureus* clinical isolates and able to prevent MRSA biofilm formation *in vitro*, suggesting that phage therapy may be a promising approach for preventing device colonization and controlling biofilms on surface.

P32 Isolation and production of *Staphylococcus aureus* lytic bacteriophages displaying broad host range spectrum for multiple strain types including clinical isolates: methodological challenges

JULIA SABIROVA[1], STEFANIE LETZEL[1], MANFRED ROHDE[2], JOHANNES WITTMANN[1], CHRISTINE ROHDE[1]

[1] Leibniz Institute DSMZ – German Collection of Microorganisms and Cell Cultures, Department of Microbiology, Braunschweig, Germany

[2] Helmholtz Centre for Infection Research, Central Facility for Microscopy, Braunschweig, Germany

Corresponding author: jus16@dsmz.de

Staphyloccoccus aureus belongs to ESKAPE pathogens causing significant problems in clinical and community settings. Here we have isolated a number of *S. aureus* lytic phages that could serve as starting points for the development of phage-based diagnostics and/or treatment applications. Success of our enrichment procedure critically depended on the number of consecutive enrichment rounds and on the choice of an appropriate host strain for the enrichment. Employing either a restriction-modification (RM)-minus, or an RM prophage-minus host strain, resulted both in the isolation of phages from sewage water. However, plaques obtained on the RM prophage-minus strain were turbid and eventually disappeared upon restreaking, possibly indicating this strain might become lysogenic. By contrast, the RM-minus strain gave rise to plaques that could reliably be proliferated. When testing clinical isolates as possible target strains, only few of these yielded plaques and this only after three enrichment rounds. EM observations of the filtrates confirmed presence of phages. When subsequently testing host range specificity of the phages against a battery of diverse *S. aureus* strains, three different outcomes were observed for target strains infected with a given phage: (1) strains forming lytic zones and plaques upon serial dilution of the filtrate; (2) strains forming lytic zones but never individual plaques; (3) strains forming neither lytic zones nor plaques. Evaluation of host specificity of newly isolated phages that trigger not only primary lysis but also reliably proliferate further (group 1) and hence represent the group of interest for application purposes, yielded coverage rates of the tested strain battery of up to 83 %. On-going work focuses on further characterization of new phages, striving to obtain as broad as possible ranges of strain specificity, and also on the determination of the most appropriate host strain for a given phage, allowing for its reliable and scalable production.

Sb-1 and Pyo-phage are active against *S. aureus* clinical isolates and able to prevent MRSA biofilm formation *in vitro*, suggesting that phage therapy may be a promising approach for preventing device colonization and controlling biofilms on surface.

P33 Characterization of biology and genetics of newly isolated bacteriophages active against *Proteus spp.*

NINA TIKUNOVA, VERA MOROZOVA, YULIA KOZLOVA, ELIZAVETA SHED‘KO, TATIANA USHAKOVA, IGOR BABKIN
Institute of Chemical Biology and Fundamental Medicine SB RAS, Laboratory of molecular Microbiology, Novosibirsk, Russia
Corresponding author: tikunova@niboch.nsc.ru

Proteus bacteria, mostly *P. mirabilis*, are etiological agents of intestinal and urinary tract infections and can infect surgical wounds and contribute to diabetic foot ulcers. *Proteus spp.* can cause chronic renal inflammation being one of the main causes of catheter-associated urinary tract infections. Clinical isolates of *P. mirabilis* often have multidrug resistance that requires the development of novel therapeutics for *Proteus* infections. Lytic bacteriophages could be a part of the combine antibacterial therapy. However, studies on bacteriophages specific for *Proteus spp.* are rather limited and genome sequences of only ten *Proteus* phages are available in the GeneBank database. Of the available *Proteus* phages, we have isolated and characterized seven bacteriophages. Electron microscopy and complete genome sequencing demonstrated that bacteriophages PM16 and PM75 belong to the genus Phikmvvirus, bacteriophages PM85, PM93, and PM116, to the SP6virus, bacteriophage PM135 to the T5virus, while PM87 is unclassified member of the Siphoviridae family. Genome sequences of bacteriophages PM16, PM 75, and PM 135 demonstrated low nucleotide homology with sequences of other bacteriophages belonging to Phikmvvirus and T5virus genera, respectively, while the genomes of PM85, PM93, and PM116 are differ from each other but similar to the *Proteus* phage genome presented in the GeneBank database. All isolated phages were specific to various *P. mirabilis* strains, including clinical isolates. In addition, bacteriophages PM116 and PM135 exhibited lytic activity on *P. vulgaris* strains. Phage adsorption, burst size, and one-step growth experiments have been carried out. Based on lytic activity, host range and the occurrence of low phage resistance bacteriophages PM16, PM75, PM85, PM93, and PM116 could have a potential to be used as a part of therapeutics in the case of infections caused by *P. mirabilis* with multidrug resistance.

P34 Our experience in personalized phage therapy of patients with diabetic ulcers

NINA TIKUNOVA, VERA MOROZOVA, YULIA KOZLOVA, DENIS GANICHEV, VALENTIN VLASSOV
Institute of Chemical Biology and Fundamental Medicine SB RAS, Laboratory of molecular microbiology, Novosibirsk, Russia
Corresponding author: tikunova@niboch.nsc.ru

Antibiotic-resistant bacteria become a global threat now. A promising approach to solve the problem is implementation of bacteriophages - viruses that can destroy bacteria, but not able to infect humans. Bacteriophages usually do not cause side effects and are not toxic. Unlike antibiotics, bacteriophages are mostly species- and strain-specific and therefore, do not disrupt the normal human microbiome. In Russia, there are approved phage preparations and cocktails, but most of them were developed in the last century and their characteristics do not meet recent requirements. In addition, the main criterion for the application of therapeutic bacteriophage should be sensitivity of bacterial agent to the bacteriophage. This requires a fast and qualitative microbiological analysis, rapid selection of the appropriate therapeutic phage or a cocktail, and microbiological control of treatment. In ICBFM SB RAS, the approach to personalized implementation of phage therapy was developed and tested. A number of original and unique bacteriophages that are effective against a broad spectrum of bacteria were isolated and characterized. Several new phage cocktails, including a unique polyspecific cocktail containing bacteriophages against 11 species of pathogenic and nosocomial bacteria, were elaborated. Clinical data on personalized application of phage preparations for the treatment of diabetic foot ulcers were analyzed. The use of phage therapy in the clinic provided the healing of most patients, whose diseases were caused by resistant bacteria and previous antibiotic therapy was unsuccessful.

P35 SicoCare Bacteriophage Solution for Skin Health Care

IMA GHAELI[1], JOANNES JANSSEN[2]
[1] SicoCare (under incorporation), Research, Porto, Portugal
[2] SicoCare (under incorporation), Finance, Porto, Portugal
Corresponding author: ema.ghaeli@gmail.com

Chronic wounds: Prevalence of chronic skin conditions, such as pressure ulcers, diabetic foot, also burn injuries and other skin related infections is on the rise. Worldwide, 15 million patients with access to reasonable health care services are in need of chronic wound treatment. This number will rise to around 25 million patients in year 2025, demanding bioactive wound care products that provide efficient cure for chronic wounds, optimizing each stage of the healing process.

Wound Healing: Wound healing is a dynamic and complex process of tissue regeneration. It requires a suitable environment to facilitate and speed up the healing process.

The SicoCare solution: A dressing is a wound healing biomaterial applied to a wound to accelerate healing and protect the wound from further harm. SicoCare is developing bioactive wound care dressings, containing bacteriophages that act efficientlyin curing extensive burn injuries and chronic wounds. The first step will be to develop a smart product with these advanced characteristics:

- automatic release of the antibacterial agent (bacteriophage) as long as the infection exists
- pain relief through decreasing the frequency of wound dressing renewal
- healing at a faster rate
- „smart dressing“ with colour alteration, alerting staff to change the dressing
- continuous moist environment
- elasticity / conformability

The company SicoCare is being established in Portugal and Germany. It is presently in the “seed” (first) stage of the startup. The research center will be in Portugal:

- availability of highly skilled staff
- low labour cost
- strong venture capital participation, especially in biotech

Marketing will focus on Germany

- innovative market
- large market for the product line
- very high health care standards

SicoCare is looking for:

- co-operation for EU- and German research projects
- combined marketing efforts
- product development opportunities
- financing solutions, access to venture capital, cross-participations

P36 Spontaneous mutants of staphylococcal polyvalent bacteriophages with broad lytic host-range on *Staphylococcus aureus* strains are suitable for phage therapy

JIRI DOSKAR[1], TIBOR BOTKA[1], RENÁTA KARPISKOVA[2], IVANA KOLACKOVA[2], IVANA MASLANOVA[1], VLADISLAVA RUZICKOVA[1], ROMAN PANTUCEK[1]
[1] Masaryk University, Dep.of Experimental Biology, Brno, Czech Republic
[2] Veterinary Research Institute Brno, Czech Republic
Corresponding author: doskar@sci.muni.cz

Due to a high lytic activity and broad host range towards *Staphylococcus aureus* strains, the polyvalent Twort-like bacteriophages of genus Kayvirus, family Myoviridae represented by phage 812 are already used for phage therapy in the Czech Republic.
From this phage spontaneous mutants can be isolated as rare plaques on resistant staphylococcal strains. Recently 15 phage mutants were characterized on genomic, proteomic and structural level. We determined their lytic effect on a set of 200 human and livestock-associated MRSA isolates. Phage 812 or its mutants were able to lyse 97 % of the MRSA livestock strains and 86 % of human MRSA strains. The lytic effect is preserved also in phage cocktails, therefore the novel mutants could be used for innovation of recent phage preparations in order to increase their effect on currently circulating strains.
To assess the safety of phage 812 mutants for phage therapy, complete genome sequences were determined and compared to the wild-type phage 812. We have found that the mutants differ from the original genome mostly by short indels. Mutations seem to affect the host range in two ways: either interfere with adsorption genes (tail-fibre) and lysis (endolysin), or affect sequences that are the target of bacterial defence mechanisms such as RM-systems or CRISCP-Cas. Mutations do not induce lysogenic conversion and transduction nor virulence factors production. Therefore, the mutants thus obtained can be considered safe for phage therapy.

Acknowledgement: Research was supported by a grant of Ministry of Agriculture of the Czech Republic No. QJ1510216.

P37 Wax moth (*Galleria mellonella*) *in vivo* model exposes therapeutic potential of *Shigella sonnei* phage cocktails

JOANNA KACZOROWSKA, DOUWE VAN SINDEREN, JENNIFER MAHONY
University College Cork, School of Microbiology, Ireland
Corresponding author: jkaczorowska.blur@gmail.com

Ten percent of the global child deaths are caused by diarrhoeal diseases during the first days of life. Most of these deaths occur in the so-called developing countries, especially in the sub-Saharan Africa and south Asia. *Shigella ssp.* and enterotoxigenic *Escherichia coli* are the most common etiological agents of these diseases. Continued development of antibiotic resistance in pathogens and the potential negative impact on the development of the infant commensal microbiota are the main drawbacks of antibiotic therapies. Bacteriophages (or phages) represent an alternative tool which can be used to specifically treat various bacterial infections, including diarrhoeal diseases. In this study, we screened waste water samples for the presence of phages and isolated 28 individual phages infecting *Shigella sonnei* 53G. These 28 phages showed wide spectrum of infection against ECOR library, covering almost all of the 72 *E. coli* strains. Genome sequencing of 10 chosen phages revealed three distinct groups of phages, with significant diversity evident in the receptor binding protein region of phages. These 10 phages have been tested regarding their survivability in low pH. The phages have been pooled together to create a cocktail, which was tested *in vivo* using the *Galleria mellonella* (wax moth) larvae model. A single dose of the phage cocktail administered two hours before (prophylaxis model) or after (remedial model) infection with *E. coli* ECOR62 successfully improved survival rate of the larvae. On the other hand, the phage cocktail applied against a mixture of five ECOR strains increased the larvae survival rate in the prophylaxis model only.

P38 Microbiome and stable core virome after human fecal transfer

KARIN MOELLING[1], FELIX BROECKER[2]
[1] University Zürich, Institute of Medical Microbiology, Switzerland
[2] Icahn School of Medicine at Mount Sinai, Microbiology, New York, US
Corresponding author: moelling@molgen.mpg.de

We recently described the 4.5-year time course of the enteric bacterial microbiota and virome of a patient cured from recurrent *Clostridium difficile* infection (rCDI) by fecal microbiota transplantation (FMT). We analyzed bacterial and viral compositions donor using 16S rRNA gene and metagenomic sequencing. The virome contained dsDNA viruses, mainly Caudovirales phages. Unexpectedly, sequences related to giant algae-infecting *Chlorella* viruses were also identified. Our findings indicated that intestinal viruses can be implicated in the establishment of gut microbiota, as phages and their host bacteria were frequently co-detected [1, 2]. Moreover, we found the patient's phage population to exhibit highly donor-similar characteristics, which remained stable for up to 7 months. This was unexpected since enteric viromes are normally highly variable, assumed to influence the bacterial host community and change with environmental conditions. In contrast to the virome, the bacterial microbiota varied indeed for more than seven months with ongoing dysbiosis before it reached donor similarity 4.5 years post-FMT [3, 4]. Our findings that are based on sequence information and protein domain analysis seem to suggest that stable phage properties correlate with successful FMT better than the changing bacterial communities. We speculate that we here preferentially detected a stable core virome, which dominated over a variable flexible virome that may have been too heterogeneous for experimental detection, or was underrepresented in the databases. The virome is possibly the determining factor in the composition of the gut microbiome and stool transfer.

It will be interesting to analyze whether the enteric virome allows for predicting the clinical outcome of FMT for rCDI and other diseases such as inflammatory bowel disease or obesity.

1. Broecker F, Kube M, Klumpp J, Schuppler M, Biedermann L, Hecht J, Hombach M, Keller PM, Rogler G, Moelling K. Analysis of the intestinal microbiome of a recovered *Clostridium difficile* patient after fecal transplantation. Digestion 2013;88(4): 243-51.
2. Broecker E, Klumpp J, Schuppler M, Russo G, Biedermann L, Hombach M, Rogler G, Moelling K. Long-term changes of bacterial and viral compositions in the intestine of a recovered *Clostridium difficile* patient after fecal microbiota transplantation. Cold Spring Harb Mol Case Stud 2016;2(1): a000448.
3. Broecker F, Russo G, Klumpp J, Moelling K. Stable core virome despite viable microbioame after fecal transfer. Gut Microbes 2017;8(3):214-20.
4. Moelling K, Broecker F. Fecal microbiota transplantation to fight *Clostridium difficile* infections and other intestinal diseases. Bacteriophage 2016;6(4):e1251380.

P39 Intratracheal application of a lytic phage against *Acinetobacter baumannii pneumonia* in mice

SANDRA-MARIA WIENHOLD[1], MARKUS BRACK[1], GERALDINE NOUAILLES[1], NORBERT SUTTORP[1], CLAUDIUS SEITZ[2], ANTON ROSS[2], HOLGER ZIEHR[2], KRISTINA DIETERT[3], ACHIM D GRUBER[3], MANFRED ROHDE[4], CHRISTINE ROHDE[5], MARTIN WITZENRATH[1]

[1] Charité – Universitätsmedizin Berlin, Dep. of Infectious Diseases and Pulmonary Medicine, Germany

[2] Fraunhofer Institute for Toxicology and Experimental Medicine (ITEM), Dep. of Pharmaceutical Biotechnology, Braunschweig, Germany

[3] Freie Universität Berlin, Dep. of Veterinary Pathology, Germany

[4] Helmholtz Centre for Infection Research, Braunschweig, Germany

[5] Leibniz Institute DSMZ – German Collection of Microorganisms and Cell Cultures, Braunschweig, Germany

Corresponding author: sandra.wienhold@charite.de

Chronic lung diseases are frequently complicated by lung and airway infections. Today, increasing number of multidrug-resistant bacteria, including *Acinetobacter baumannii*, has given rise to serious concerns. High specificity of bacteriophages for their bacterial hosts and their effectivity in lysis make phage-therapy attractive to overcome this problem. However, phages need to be properly chosen and characterized according to scientific state-of-the-art technology. For safe application, preparations must be highly purified and free of endotoxins.

This preclinical pilot study aims at determining the efficacy, safety and tolerability of a phage preparation, in anticipation of a future clinical trial applying aerosolized lytic phages against gram-negative bacteria in patients with chronic airway infection.

The specific Phage Acibel004 [1] was produced as high-titer suspension subjected to final processing including highly efficient multi-step depletion of endotoxins by column affinity chromatography. Mice were transnasally infected with *A. baumannii* and 12 h p.i. treated with phage or solvent intratracheally. Bacterial burdens in relevant organs were determined. Leukocytes were differentiated, clinical parameters measured and histopathological analyses performed. Furthermore, presence of vital phages was determined.

Phage application led to a significantly higher survival rate. Treated mice recovered faster from infection-associated loss in body temperature. Furthermore, clinical outcome of phage-treated mice was improved compared to solvent treatment. Bacterial loads in lungs and BALF of the phage-treated group were significantly reduced by 48 h p.i. Phages were detected in BALF, lungs and plasma of the infected and treated mice. The numbers of immune cells were unaffected by phage treatment. No adverse effects were observed.

The current preclinical data further support the concept of developing a phage-based therapy against pulmonary *A. baumannii* infections.

1. Merabishvili M, Vandenheuvel D, Kropinski AM, Mast J, De Vos D, Verbeken G, Noben JP, Lavigne R, Vaneechoutte M, Pirnay JP. Characterization of newly isolated lytic bacteriophages active against *Acinetobacter baumannii*. PLoS One. 2014;9:e104853.

P40 Isolation and characterization of lytic bacteriophages from different sources active against bacteria associated with prosthetic joint infections

ANN-BRIT KLATT, ANDREJ TRAMPUZ, MARIAGRAZIA DI LUCA
Charité – Universitätsmedizin Berlin, Berlin, Germany
Corresponding author: ann-brit.klatt@charite.de

In recent years, phage therapy has attracted attention as a promising alternative treatment option for biofilm-associated infections. To establish a successful phage therapy, access to a comprehensive stock of different phages covering a broad bacterial spectrum is crucial. Aim of this study was to screen human and environmental sources for presence of lytic phages of selected bacterial strains.

Saliva samples and sewage water were screened for presence of lytic phages active against 20 and 10 clinical strains of *Staphylococcus aureus* and *Escherichia coli*, respectively, isolated from patients with prosthetic infections. A clinical isolate of *Pseudomonas aeruginosa*, pan-drug resistant, and laboratory strains of methicillin-resistant *S. aureus* (MRSA) ATCC 43300 and *E. coli* ATCC 25922 were also tested. Detected phages were isolated and lytic activity against host strains was evaluated by crystal violet and isothermal microcalorimetry. Phages were further characterized by transmission electron microscopy.

Six bacteriophages specific for MRSA ATCC 43300 were isolated from saliva. Bacteriophages for *E. coli* strains were isolated both from sewage water (n=3) and saliva samples (n=1), each for one different clinical strain. Bacteriophages active against pan-drug resistant *P. aeruginosa* strain were found in both source (n=3). Morphological analysis performed by TEM revealed that the isolated phages seem to belong to different families. Treatment of bacterial host strains biofilms with phages determined a considerable reduction of total biomass, up to 39 % and 84 % in the case of MRSA and *E. coli*, respectively.

Both sewage and saliva samples provided bacteriophages specific against selected bacterial strains associated with prosthetic joint infections. Phages specific for *E. coli* are more distributed and more common in screened samples compared to MRSA-specific phages. Moreover, 24 h phage treatment of *E. coli* and *S. aureus* biofilms lead to a reduction, although a complete eradication was not observed in the tested conditions.

P41 Three cases of ultima ratio bacteriophage therapy in the clinic for cardiothoracic, transplantation and vascular surgery

EVGENII RUBALSKII[1], ANDREY ALESHKIN[2], CHRISTIAN KÜHN[1], BAKR MASHAQI[1], IRINA KISELEVA[2], SVETLANA BOCHKAREVA[2], CHRISTINA SALMOUKAS[1], STEFAN RÜMKE[1], ELDAR ZULKARNEEV[2], OLGA BASHKINA[3], KHALIL GALIMYZANOV[3], OLEG RUBALSKY[3], AXEL HAVERICH[1]

[1] Hannover Medical School (MHH), Department of Cardiothoracic, Transplantation and Vascular Surgery (HTTG), Germany

[2] G.N. Gabrichevsky Moscow Research Institute for Epidemiology and Microbiology, Moscow, Russia

[3] Astrakhan State Medical University, Russia

Corresponding author: Rubalskii.Evgenii@mh-hannover.de

Increasing antibiotic resistance is a significant worldwide challenge, especially in medicine. The development and spread of antibiotic resistance has led to the emergence of multidrug- and even pandrug-resistant bacteria. Complications related to bacterial infection during cardiothoracic and vascular surgery are often very hard to treat due to biofilm-mediated antibiotic tolerance. Bacteriophage therapy is a promising alternative for the patients who do not respond to conventional antibiotic therapy. Phages can be used as ultima ratio therapy within the framework of the article 37 of the Declaration of Helsinki. We have successful experience with such application of personalized phage preparations in 3 patients. Patient 1 had a chronic mixed bacterial vascular graft infection of the aortic arch complicated by pleural empyema. Patient 2 was under immunosuppression therapy after heart transplantation and developed pan-resistant *Klebsiella pneumoniae* infection of lungs. Patient 3 had chronic *Staphylococcus aureus* vascular graft infection of the aortic arch. All the patients obtained bacteriophages via both local and oral roots of administration. Standard antibiotic therapy was also continued. Eradication of the pathogenic bacteria was microbiologically confirmed in the sites of infection in all 3 cases. Patient 1 survived more than 2 months after phage therapy, but later died due to new bacterial infection. Patients 2 and 3 were discharged from our clinic without signs of infection. Patient 2 shows 9-months survival. Patient 3 shows 5-months survival, but is still under periodical PET-CT control.

P42 Fibrin glue as a local drug-delivery system for bacteriophages

EVGENII RUBALSKII, BAKR MASHAQI, CHRISTINA SALMOUKAS, STEFAN RÜMKE, ERIN COLLEEN BOYLE, CHRISTIAN KÜHN, AXEL HAVERICH
Hannover Medical School (MHH), Department of Cardiothoracic, Transplantation and Vascular Surgery (HTTG), Germany
Corresponding author: Rubalskii.Evgenii@mh-hannover.de

The aim of the present study was to evaluate whether bacteriophages could be encorporated in fibrin glue for prolonged, localized phage release. Purified bacteriophages in 0.9 % NaCl solution were mixed *ex tempore* with the thrombin component of a fibrin glue (Baxter Deutschland GmbH). The initial titer of each phage was above 1×10^{11} pfu/ml. NaCl solution without bacteriophages was used as a negative control. The addition of the NaCl-bacteriophage solution to the fibrin glue did not affect the mechanical properties of the glue. The kinetics of bacteriophage release from the fibrin glue was demonstrated with the *Pseudomonas aeruginosa* phage PA5. Release of fibrin glue-incorporated bacteriophages was compared to fibrin glue samples soaked in a solution of PA5 phage and the same concentration. Fibrin glue samples were placed separately a saline solution and incubated at 37 °C with constant shaking. Phage titers in the saline solution were measured over time. After 4 days, fibrin glue with incorporated bacteriophages released more than 10 times higher phage titers compared to fibrin glue soaked in bacteriophages. Peak bacteriophage release was observed after complete dissolving of the fibrin glue samples with incorporated phages. The kinetics of bacteriophage release belonging to different families, having various virion sizes and host range activity will be tested as well as in vitro efficacy of the phage-containing fibrin glue. Under the adequately selected concentration and host range activity, bacteriophages are likely to be a powerful antibacterial addition to fibrin glue for treatment and prevention of perioperative infections.

P43 *Staphylococcus* phage SSP134 specific to a wide range of coagulase-negative *staphylococcus* species

YULIYA KOZLOVA, VERA MOROZOVA, ARTEM TIKUNOV, NINA TIKUNOVA
Institute of Chemical Biology and Fundamental Medicine SB RAS, Laboratory of Molecular Microbiology, Novosibirsk, Russia
Corresponding author: ulona@ngs.ru

Drug-resistant staphylococci are leading causes of hospital-acquired infections. Recent years coagulase-negative staphylococci (CoNS) became causative infectious agents in human and domestic animals.

The bacteriophage SSP134 was isolated from the swab taken from the axillary area of the volunteer. The host range of phage SSP134 was determined using 175 strains of genus *Staphylococcus* from Collection of extremophilic microorganisms and type cultures of ICBFM SB RAS. *Staphylococcus* strains were previously isolated from humans and animals. Eightynine coagulase-positive strains, including *S. aureus*, *S. pseudintermedius/intermedius*, and eightysix coagulase-negative strains were tested. It was revealed that phage SSP134 was active against a range of 26 strains including *S. aureus*, *S. warneri*, *S. simulans*, *S. epidermidis*, *S. haemolyticus*, *S. equorum*, *S. capitis*, and *S. succinus* strains. Thereby, SSP134 was a lytic phage that infected mostly coagulase-negative staphylococci.

According electron microscopy phage SSP134 was classified as Podoviridae family member. NGS sequencing revealed linear double-stranded DNA genome with the size of 18275 bp. Genome analysis of the sequence identified 20 potential ORFs. The genome was submitted to GenBank (accession number KY471386). According to phylogenetic analysis of SSP134 genome and related sequences from GenBank Database this phage was classified as a member of genus P68virus.

Thus, phage SSP134 may be a potential candidate for therapy of CoNS infections.

P44 The efficacy of *Salmonella*-specific bacteriophage in weaned piglets challenged with *Salmonella typhimurium*

JEONGHEE HAN[1], SUNGJAE KIM[1], YOUNGIHN KIM[2]

[1] Kangwon National University, Institute of Veterinary Science, College of Veterinary Medicine, Songpa-gu, Seoul, Republic of Korea

[2] CTCBIO, Republic of Korea

Corresponding author: hanjh@kangwon.ac.kr

Salmonellosis which is mainly caused by S*almonella (S.) typhimurium* has been a constant problem in swine industry. Recently, excessive use of antibiotics is being discouraged in livestock industry because of an emerging crisis of antimicrobial resistance. Bacteriophages which can lysis specific bacteria are considered as alternative antimicrobial sources.

The healthy 3-weeks-piglets were randomly allocated into 4 groups (NB-NC, NB-C, B-NC and B-C). NB-NC was non-treatment group. B-NC and B-C were fed with 1 g of *S. typhimurium*-specific bacteriophage (1x10^8 PFU/g, CTCBIO Co., Korea) per 1kg feed from 1st day. NB-C and B-C were inoculated orally with 5ml of *S. typhimurium* (1x10^{10} CFU/ml) per one piglet on the 7th day. Body weights and feed intakes were recorded daily to calculate ADG and FCR. The severity of diarrhea was checked daily and fecal samples were collected at 0, 1, 3 and 7 dpi for quantitative analysis of the antigen by real-time PCR. All animals were euthanized for necropsy on 14th day. Intestine tissues (jejunum, ileum and colon) were collected for quantitative analysis of the antigen and H&E stain. V/C ratio (Villus height: crypt depth) were measured by microscopic observation to evaluate intestinal integrity.

There were no significant differences between the groups before the challenge during the period before the challenge. Otherwise, in the growth performance and diarrhea, B-C was significantly improved compared with NB-C after the challenge. In quantitative analysis of the antigen, NB-C was higher than B-C in fecal sample, and ileum and colon, significantly. In V/C ratio, B-C was higher than NB-C, significantly. Overall, feeding supplemented with bacteriophages improved growth performance and intestinal integrity by reducing the level of infection.

P45 Application of phages in poultry farming as One Health approach

IMKE SCHMIDT[1], ANNA BIERBRODT[2], JOHANNES WITTMANN[1], SOPHIE KITTLER[3], RUTH MENGDEN[3], TATIANA LEHNHERR[4], MICHAEL FINK[2,4], CHRISTINE ROHDE[1], MANFRED ROHDE[5], HANSJÖRG LEHNHERR[4]

[1] Leibniz Institute DSMZ – German Collection of Microorganisms and Cell Cultures, Braunschweig, Germany
[2] FINK TEC GmbH, Hamm, Germany
[3] University of Veterinary Medicine Hannover, Foundation, Institute for Food Quality and Food Safety, Germany
[4] PTC Phage Technology Center GmbH, Bönen, Germany
[5] Helmholtz Centre for Infection Research, Braunschweig, Germany
Corresponding author: ims16@dsmz.de

Escherichia coli is among the most common pathogens in poultry and the causative agent of colibacillosis. This frequent disease creates significant economic losses and is commonly treated by antibiotics, which in turn promotes the selection of multidrug resistant (MDR) bacteria. A high incidence of MDR bacteria is problematic not only for animal health but also because of the potential for zoonotic transfer to humans through contaminated food. Therefore, there is a high demand to develop new strategies as alternatives to antibiotic therapies. In accordance with the One Health concept that was a leading theme during the 2016 World Health Summit, which interfaces human, animal, and environmental health, this project aims to isolate and characterize phages to fight *E. coli* infections in broilers. As a proof of principle broilers will be treated prophylactically with a phage preparation and infected artificially with a traceable strain to distinguish it from the commensal flora. If successful *in vivo*, the use of a phage preparation could reduce the application of antibiotics in livestock farming.

Thus far, we isolated phages from sewage (21 phages), surface water (4), manure (34), poultry (18) respectivily horse dung (1), and hospital wastewater (5). The characterization revealed a diversity of morphotypes (77 % Myo-, 17 % Sipho-, and 6 % Podoviridae) and similarities in particular to T4- and T5-like phages. Host range analyses were performed by spot tests on 72 *E. coli* strains with different resistance patterns.

We identified E28 as suitable strain for *in vivo* experiments due to its kanamycin and potassium tellurite resistances. 11 of the isolated phages infect E28 with varying efficiency. In order to avoid the development of phage resistance we composed a mixture of virulent phages that differ in host coverage, genotype, growth parameters, and inhibit the growth of E28 *in vitro*.

P46 Phages application to control *Pseudomonas aeruginosa* contaminations from terminal water points of use

VANESSA MAGIN[1], NATHALIE GARREC[1], YVES ANDRES[2]
[1] CSTB Centre scientifique et technique du bâtiment, Plateforme Aquasim, Nantes, France
[2] IMT, Institut supérieur Mines Telecom Atlantique, Nantes, GEPEA, Génie des Procédés Environnement et Agroalimentaires, Nantes, France
Corresponding author: vanessa.magin@cstb.fr

Pseudomonas aeruginosa belongs to the group of multidrugs resistant bacteria. This microorganism is an opportunistic pathogen present in some water network. Contaminations are often located at terminal point-of-use, especially in hospital and thermal institutes giving rise to sanitary and economical risks. While, different processes are routinely used to treat water outlets, some contaminations remained persistent due to the formation of biofilm. The penetration barrier provided by biofilms exopolymers accumulation around cells, preventing biocides action. In this context, the research challenge is focused on the development of an alternative treatment which could be, in addition to existing methods, participate to *P. aeruginosa* biofilms elimination of this kind of surfaces.

Bacteriophages are exclusively natural predators viruses of bacteria. They are ubiquitous in the environment allowing us to have a wide diversity available. Study‘s aim will be to test the potential efficiency of phages as biocontrol agent against *Pseudomonas aeruginosa*.

Seven *Pseudomonas aeruginosa* strains included the reference strain PA01 and environmental strains were used to studied activity of 9 bacteriophages. Two phages have been isolated from the environment with double layer plate method and 7 others have been previously described. Bacterial strains were cultivated in minimum medium and efficiency of phages was studied on exponential culture, stationary phase or biofilm by optical density (OD 600 nm) analysis and vPCR.

This study highlights the specificity of some phages towards environmental strains of *P. aeruginosa* studied. Bacteria susceptibility depends of their physiological cells state (included planktonic or sessile) but also of the phage titer used. While phages can specifically reduce the population of *P. aeruginosa* strain, resistance effects can be observed and must be taken into account. In this way, further investigations are needed to optimize phages infection. One issue will be to associate phages activity in one cocktail with broad host range spectrum.

P47 The use of *Salmonella* bacteriophages in bearded dragons: pilot study on the application, passage time, effectivity, and reisolation

KEVIN RENFERT[1], WOLFGANG RABSCH[2], MICHAEL PEES[1]

[1] Universität Leipzig, Klinik für Vögel und Reptilien, Leipzig, Germany

[2] Robert Koch Institute, National Reference Centre for Salmonella and other bacterial Enterics, Wernigerode

Corresponding author: kevin.renfert@vogelklinik.uni-leipzig.de

Introduction
Reptile and Exotic Pets Associated Salmonellosis (REPAS) in humans, e. g. caused by bearded dragons, has been documented as zoonotic risk. This pilot study aimed to provide information on the possible use of *Salmonella* phages in reptiles.

Material and Methods
Two *Pogona vitticeps* were used to establish an effective application scheme and examine the shedding period of specific phages, as well as the bacterial flora following application. Stomach pH was determined and drugs were tested to increase the pH in order to allow phage passage into the intestines. After application of an O1-Phage, feces and swabs were collected for reisolation and examination for *Salmonella* serovars as well as accompanying intestinal flora.

Results
A regular stomach pH of 1-2 was found that could be increased to 3 or 4 using buffer solutions. Phages could successfully be reisolated from day 11 to at least 36 days after application. With the phages used, no significant effect on the excretion of *Salmonella* and no change in the intestinal flora were determined.

Conclusion
Phages can successfully be applied to reptiles, pass the intestines and be shed over a prolonged period. Further research is necessary to determine the effect to *Salmonella* shedding.

P48 Engineered phages for efficient control and rapid detection of viable *Erwinia amylovora* cells

LARS FIESELER[1], YANNICK BORN[1], BRION DUFFY[1], MARTIN LOESSNER[2]
[1] Zurich University of Applied Sciences, Life Sciences, Wädenswil, Switzerland
[2] ETH Zurich, Institute of Food, Nutrition and Health, Switzerland
Corresponding author: lars.fieseler@zhaw.ch

Erwinia amylovora causes fire blight, a devastating disease of Rosaceae plants. Fire blight leads to high economical losses in the global pome fruit industry every year. It can be reliably controlled by antibiotics (mainly streptomycin). However, alternatives are in demand due to the emergence of resistant strains and regulatory restrictions. Currently, detection of fire blight relies on immunological, culture- or PCR-based methods. Weak points of these methods are the lack of sensitivity, low speed or the inability to discriminate between live and dead bacteria. Owing to their high host specificity, bacteriophages are promising alternatives for both control and detection of bacteria. We have previously isolated and characterized *E. amylovora* phages and investigated their ability to control bacterial growth. We observed a synergistic effect between L1, a T7-like podovirus, and the myovirus Y2. A depolymerase enzyme encoded by L1 (DpoL1) is responsible for the synergism. DpoL1 is part of the tail fibers and it degrades the exopolysaccharide capsule of *E. amyolovora*. To enhance biocontrol efficacy of Y2, we introduced a truncated version of the depolymerase gene (dpoL1-C) into the genome. The additional DNA had no adverse effects. Y2::dpoL1-C showed a better control efficacy *in vitro* than its parental phage and significantly reduced cell numbers of *E. amylovora* on detached flowers. In addition, Y2 was genetically engineered to create a bioluminescent reporter phage. To do so, a luxAB gene fusion of *Vibrio harveyi* was introduced into the genome of Y2. Infection of *E. amylovora* by Y2::luxAB induced the expression of luciferase, which enabled the rapid detection of viable *E. amylovora* cells by means of light-emission with a detection limit of 3.8×10^3 CFU/ml within 50 min.

P49 Disposable phage-based electrochemical biosensors for *Listeria monocytogenes*

JÚLIA MAGALHAES[1], CLÁUDIO ALMEIDA[1], VÂNIA FERREIRA[2], PILAR GONÇALVES[1], PAULA TEIXEIRA[2]

[1] LAQV/REQUIMTE, FEUP/University of Porto, Porto, Portugal

[2] CBQF, Escola Superior de Biotecnologia, Centro Regional do Porto da Universidade Católica Portuguesa, Porto, Portugal

Corresponding author: jmagalh@fe.up.pt

Foodborne diseases represent a global public health challenge. A key challenge to determine the safety of food and guarantee a high level of consumer protection is the availability of fast, sensitive and reliable methods to identify specific hazards before they become a health problem. Gastrointestinal disease caused by *L. monocytogenes* is rare compared to other agents of foodborne illness, but invasive listeriosis can be very severe and has a high fatality.

Electrochemical biosensors for bacterial cells detection have attracted considerable interest due to their low cost, high speed, simplicity and the accurate and precise results they provide. The biological recognition element is a central point in biosensor design. Lytic phage offer several possibilities to develop label-free biosensors for bacteria detection and it is possible to use them both as highly selective receptors and as an agent to promote cell lysis thus offering a pool of biochemical markers for detection of microorganisms.

In this work, two types of label-free potentiometric biosensors for *Listeria monocytogenes* which detected respectively the binding and the lysis are presented. The biosensors were obtained by coupling *L. monocytogenes* lytic phages with polymeric membrane ion selective electrodes. A shift in the measured potential difference was observed upon the phage-bacteria binding event. This response is related to changes in the ion diffusion layer provoked by this event. The lysis event was detected monitoring the released intracellular ions. The development of these biosensors using phages and potentiometric detection with paper based analytical devices provides rapid, reliable, low-cost and easy to use devices for detection of this pathogen.

P50 Application of bacteriophages in poultry

TARAS GABISONIA, MANANA LOLADZE, NATELA CHAKHUNASHVILI, MANANA NADIRADZE, MAIA ALIBEGASHVILI, NATIA TAMARASHVILI, TAIMURAZ KATAMADZE, TAMAR KALANDARISHVILI, TATIANA ELIAVA

Eliava Institute of Bacteriophages, Microbiology and Virology, Applied Microbiology, Tbilisi, Georgia

Corresponding author: tato_gabisonia@yahoo.co.uk

Introduction: Food-borne pathogens are a major threat to food safety. Food animals are the primary reservoir for the most food-borne pathogens. The use and misuse of antimicrobials in both humans and animals have given rise to the emergence of infectious bacteria displaying resistance toward many, and in some cases all, effective antimicrobials. Widespread occurrence of multi resistance in various species of domestic and wild animals represents a serious problem with regard to public health protection. Existence of such pathogens is problematic because of possible transmission of antibiotic-resistant bacteria from animals to humans through the food supply. Bacteriophages are considered as an important alternative for biocontrol of such pathogens.

Aim: The aim of our work was to study the effectiveness of polyvalent bacteriophage preparation – “zoovetphage”, as an alternative remedy to prevent intestinal infections in poultry. The preparation is produced on the base of *Salmonella*, pathogenic *E. coli* and *S. aureus* phages.

Materials and Methods: Experiments with egg laying chickens (laying hens) were carried out at the poultry farm, near Tbilisi. 300 one-day old egg laying chickens were enrolled in this study. Chickens of first group (100 chickens) were fed with combined food, free of antibiotics or other antibacterial preparations (non-treated group). 100 chickens of second group were fed with same food as first group, but „zoovetphage“ was added to food every 2 days during 2 weeks, a total of 7 times. In the second group, 100 chickens were fed daily with food containing antibiotic tetracycline.

Results: On day 30 from start of study none of chickens died in phage treated group, 15 chickens died from non-treated group and 6 chicken died (developed colibacteriosis) in the antibiotic treated group. The effects of phage and antibiotic treatment on eggs production and quality will be studied later.

P51 Production of anti-bacteriophage antibodies for rapid detection of *Salmonella* contamination in food by lateral flow immunoassay

ANUCHA SANGWIMAN, PREEDA PHOTHAWORN, VEERACHAT MUANGSOMBUT, SUNEE KROBSRISATE
Mahidol University, Faculty of Medicine Siriraj Hospital, Department of Immunology, Bangkok, Thailand
Corresponding author: sensmith.anucha@gmail.com

Non-typhoidal *Salmonella* (NTS) infection is a major worldwide foodborne disease. *Salmonella* serovars *Typhimurium* and *Enteritidis* are the major causes of NTS. Infection in infants, elderly people and immunocompromised hosts can develop severe symptoms. Rapid detection of *Salmonella* contamination in food samples is needed to reduce risk of infection. The main goal of our study is to combine the application of bacteriophage (phage) to the lateral flow immunoassay for development of a simple, rapid, and sensitive assay. To achieve this goal, antibody specific to *Salmonella* bacteriophages is required.
Bacteriophage SE-W109, specifically infect *Salmonella enterica*, were isolated by our group. High titer of *Salmonella* bacteriophage SE-W109 was prepared by double-layer plaque assay for immunization in rabbit. To evaluate the immune responses of the injected antigen, blood samples from the rabbit were collected at regular intervals before each injection and final bleeding. Anti-*Salmonella* phage antibody titer was evaluated by indirect ELISA and the concentration was determined by Bradford protein assay. Specificity of *Salmonella* phage SE-W109 antibodies were determined by western blot analysis against various foodborne pathogens.
After purification of anti-*Salmonella* phage SE-W109 by ammonium sulfate precipitation, high titer of anti-*Salmonella* phage SE-W109 antibodies was obtained. The final concentration of antibodies was 1.04 mg/ml. Specificity of anti-*Salmonella* phage SE-W109 was determined by Western blot analysis. The result showed no cross-reaction against other foodborne pathogens.
Anti-*Salmonella* phage SE-W109 antibodies were successful developed. The antibodies have high titer and show specificity against *Salmonella* SE-W109 bacteriophage, no cross-reaction to other foodborne pathogens. This generated polyclonal antibody will be used for further development of lateral flow immunoassay strip to detect *Salmonella* contamination in food samples.

P52 Raw milk – a reservoir for uncommon lactococcal bacteriophages

NATALIA WAGNER, ERIK BRINKS, CHARLES FRANZ, HORST NEVE
Max Rubner-Institut, Department of Microbiology and Biotechnology, Kiel, Germany
Corresponding author: natalia.wagner@mri.bund.de

Phages present in milk fermentation processes can lead to continuous infections of lactic acid bacteria used as starter cultures and can cause fermentation delays, or even total failures of fermentation batches. We have previously shown that phages of *Lactococcus lactis* starter strains may exhibit a remarkably high thermal stability. Therefore, pasteurization of raw milk will not severely affect the viability of these thermo-resistant phages. Dairy phages can even survive in high numbers in whey powders after spray-drying. Raw milk might be a critical source for new phages. However, data on the dissemination of dairy phages in raw milk are scarce. It was reported earlier that lactococcal phages were present in 10 % of raw milk samples, and phages were detected in maximal titers of up to 10^4 plaque-forming units pfu/mL in raw milk.

In our study, we monitored 52 raw milk samples from different farms in northern Germany. A representative set of *Lactococcus lactis* starter culture isolates was used for phage monitoring. Notably, phages were widespread in raw milk and detected in 35 % of the raw milk samples. Different phage titers were determined within a wide range of less than 10^1 to unexpectedly high numbers of 10^6 pfu/mL. Transmission electron microscopic and DNA sequence analyses revealed that the majority of the raw milk phage population did not belong to the common group of 936 phages that are usually present in dairy samples, but were related to rarely found phage types. Therefore, it could be concluded that raw milk phages may not be regarded as a major source of dairy phages which have adapted to industrial milk fermentations.

P53 First steps in phage treatment among bacterial biofilms

KARLIS RACENIS[1], **DACE MATĪSA**[2], **AIVARS PĒTERSONS**[1], **JUTA KROIČA**[1]
[1] Riga Stradins University, Department of Biology and Microbiology, Riga, Latvia
[2] Pauls Stradins Clinical University Hospital, Riga, Latvia
Corresponding author: karlis.racenis@rsu.lv

Introduction: In the past decades multiresistant bacterial infection rate has been increased. *E. coli* causes around 85 % of UTI (urinary tract infection), especially biofilm producing strains make the treatment more complex. Bacteriophages have a promising effect in bacterial biofilms, they are replicating at the site of infection and produce substances that degraded biofilm. There is a few information about phage lytic activity in *E. coli* biofilms, especially in ESBL (extended-spectrum beta-lactamase).

Aim: To evaluate bacteriophage lytic activity among biofilm producing uropathogenic *E. coli* strains.

Material and methods: 45 *E. coli* strains were isolated from patients with UTI, 3 *E. coli* reference strains were used (ATCC – 33526, 35218, 25922). Biofilm production was detected by using 96 well microtiter plates. To determine biofilm production optical density was measured with ELISA reader. Biofilm production degree was calculated by each strain mean optical density compared with negative controls mean absorbance. Biofilm production was classified as “no”, “weak”, “moderate” or “strong” biofilm production. To evaluate bacteriophage lytic activity, spot test method was used. Bacteriophage lysate from “Piofag”, Mikrogen and 3 lytic *E. coli* bacteriophages were used. Phage effect was identified by plaque method.

Results: Bacteriophage lytic activity among moderate and strong biofilm producer strains was 86 % (18 out of 21). Moderate biofilm producers were 12 (27 %), strong 9 (20 %), weak 23 (51 %), no producer 1 (2 %) out of 45 *E. coli* strains. In ESBL *E. coli* strains (n = 8) 4 were moderate and 1 was strong biofilm producer. Bacteriophage lytic activity among those strains was 4 out of 5.

Conclusions: *E. coli* clinical isolates produce moderate and strong biofilms. Bacteriophages have a great lytic activity among biofilm producer strains as well ESBL *E. coli* biofilm producer strains. To determine bacteriophage effect in established biofilms, more studies should be done using different methods, e. g. Calgary device.

P54 Asymmetric flow field flow fractionation – a novel virus purification method to meet high purity standards of phage applications

MIRKA LAMPI, MINNA PORANEN, HANNA OKSANEN, DENNIS BAMFORD, KATRI ESKELIN
University of Helsinki, Department of Biosciences, Helsinki, Finland
Corresponding author: mirka.lampi@helsinki.fi

Safe and controlled use of phage therapy requires viral preparations of high quantity and purity as well as preservation of viral integrity. Toxins and impurities derived from the host bacteria can cause uncontrolled inflammation reactions and compromise the safety of the final preparation. The traditional ultracentrifugation-based purification methods may induce aggregation and lead to relatively low yields of infective viruses. In addition, traditional methods are laborious and time-consuming. Asymmetric flow field flow fractionation (AF4) provides an attractive alternative method for large scale virus purification being a rapid and gentle separation method preserving biological functionality.

We have optimized the AF4 conditions to be used for the purification of prokaryotic viruses with different morphologies, biochemical and -physical properties. Our results show that AF4 is well suited for virus purification as monitored by high recovery of infectious viruses and increased specific infectivity. Short analysis time (~50 min) and high sample loads enabled us to use AF4 for preparative scale purification of prokaryotic viruses. Furthermore, we show that AF4 allows rapid real-time analysis of progeny virus production in infected cells. The data presented here is from the purification of three different prokaryotic viruses: an icosahedral tailed virus (*Haloarcula vallismortis* tailed virus 1, HVTV-1), an icosahedral virus with an inner lipid membrane (*Enterobacteria* phage PRD1), and a spherical virus with an outer lipid envelope (*Pseudomonas* phage Φ6).

Direct AF4 purification from cell lysates resulted in high yields of infectious viruses and high specific infectivities of virus containing fractions, whereas host-derived contaminants were efficiently removed. Consequently, AF4 provides a one-step method to produce virus material from crude cell lysates for biochemical and -physical characterization of prokaryotic viruses to be used in applications with strict purity and safety standards including potential therapeutic applications.

P55 Characteristic of enterococcal bacteriophages produced in cells of improved phage propagation strains

AGNIESZKA GOZDEK[1], AGNIESZKA BEDNAREK[1], ALEKSANDRA GŁOWACKA[1], ALEKSANDRA RÓŻYCKA[1], JAN GAWOR[2], DARIUSZ IZAK[1], KAMIL DĄBROWSKI[1], AGNIESZKA FILIPEK[1], EWA SADOWY[3], ROBERT GROMADKA[2], BEATA WEBER-DĄBROWSKA[4], ANDRZEJ GÓRSKI[4], MAŁGORZATA ŁOBOCKA[1]

[1] Institute of Biochemistry and Biophysics, Polish Academy of Sciences, Department of Microbial Biochemistry, Warsaw, Poland

[2] Institute of Biochemistry and Biophysics, Polish Academy of Sciences, Laboratory of DNA Sequencing and Oligonucleotide Synthesis, Warsaw, Poland

[3] National Medicines Institute, Department of Epidemiology and Clinical Microbiology, Warsaw, Poland

[4] Ludwik Hirszfeld Institute of Immunology and Experimental Therapy, Laboratory of Bacteriophages, Wroclaw, Poland

Corresponding author: a.kielan@ibb.waw.pl

E. faecalis and *E. faecium* strains are among the main causes of nosocomial infections, due to the acquisition of antibiotic resistance, including the resistance to vancomycin. Several bacteriophages that infect *E. faecalis* or *E. faecium* are deposited in the collection of L. Hirszfeld Institute of Immunology and Experimental Therapy, PAS. Some of them have been successfully used in experimental treatment of enterococcal infections. In general, enterococcal phages propagate poorly in cells of strains that are isolated as their hosts from among clinical isolates. Typically, such strains grow slowly in media without blood or other components of animal origin. Additionally, they often contain prophages – a source of contaminating temperate phages in therapeutic phage preparations. To obtain a host for the production of monoclonal phage preparations of high titer, lacking components of animal origin, we selected an *E. faecalis* isolate that was sensitive to over 10 phages, adapted it to fast growth in Luria-Bertani medium and deprived of prophages. The resulting strain retained the sensitivity to the same phages from the collection as its parent, but the titer of phages in the lysates obtained was significantly higher. The high titer was beneficial for the extension of the number of strains infected to those that could not otherwise be infected due to the low infection efficiency. All tested phages appeared to be members of the Siphoviridae family. Preliminary analysis of their genome revealed that some of them are related to each other. Surprisingly, an additional phage of 29 kb genome (EF12c29) accompanied larger phages in a few phage preparations. It appeared to encode a potent endolysin that, when modified, could be produced in *Escherichia coli* cells and lysed dead as well as living cells of certain *Enterococcus sp* strains.

Support: Project No.POIG 01.03.01-02-003/08; statutory funds for the IBB, PAS

P56 Effects of milk viscosity on the accessibility of bacteriophages to their hosts

FIRUZE ERGIN[1], ZEYNEP ATAMER[2], MEIKE SAMTLEBE[2], JÖRG HINRICHS[2], AHMET KÜCÜCKCETIN[1]

[1] Akdeniz University, Department of Food Engineering, Faculty of Engineering, Antalya, Turkey

[2] University of Hohenheim, Institute of Food Science and Biotechnology, Department for Soft Matter Science and Dairy Technology, Stuttgart, Germany

Corresponding author: zeynep.atamer@uni-hohenheim.de

Bacteriophages represent one of the most abundant biological populations in nature and are defined as bacterial viruses that can kill specific target bacteria. The use of bacteriophages as an antibacterial agent was initiated in 1917, when Felix d'Herelle observed that bacteriophages lyse bacteria. The discovery of penicillin in 1928 caused to prevent using of bacteriophages as an antibacterial agent, however gaining resistance of currently available antibiotics to pathogenic bacteria reinforces bacteriophage applications again. In recent years, studies on the use of bacteriophages against pathogenic bacteria in food products have been increasing. The bacteriophage applications against the bacteria in food products depends on numerous factors such as phage concentration, accessibility to host bacteria and environmental challenges (pH, temperature, water activity, viscosity etc.).

It is reported that bacteriophages can be used against the pathogens. However, some milk components are preventing the adsorption of phages to their hosts. The aim of the present study is to investigate the activity of dairy bacteriophages as well as their accessibility to their host in milk with different viscosities. For the investigations, *Lactococcus lactis* phage P008 was used. Bacteriophage and host bacteria were inoculated into reconstituted skim milk having different viscosities. The titers of bacteriophages and the enumeration of host bacteria were monitored over the incubation period of 72 hours. The infectivity of phage particles was determined. The first results of this study will be presented and discussed.

P57 Purification and characterization of the Listeriophage vB_LmoS_293 endolysin and tail fibre proteins and their potential applications against biofilms of *Listeria monocytogenes*

VINCENZO PENNONE[1,2], MARTA SANZ GAITERO[2,3], AIDAN COFFEY[2], KIERAN JORDAN[1], MARK JOHAN VAN RAAIJ[3], OLIVIA MCAULIFFE[1]

[1] Teagasc Food Research Centre, Fermoy, Cork, Ireland

[2] Cork Institute of Technology, Bishopstown, Cork, Ireland

[3] Centro Nacional de Biotecnologia (CNB-CSIC), Departament of Macromolecular Structures, Madrid, Spain

Corresponding author: vincenzo.pennone@teagasc.ie

Listeria monocytogenes is a ubiquitous gram-positive bacterium that is a major concern for food business operators because of its pathogenicity and ability to form biofilms in food production environments. To date, a number of bacteriophages against *L. monocytogenes* have been isolated and some have been approved for use in food processing environments (ListShield and Listex). Listeriophage vB_LmoS_293, a Siphoviridae infecting *L. monocytogenes* serotypes 4b and 4e, was previously isolated from mushroom compost. Its lysogenic nature excluded it as a biocontrol candidate for *in situ* applications, however its proteins can be studied for biocontrol purposes. In particular, endolysins are proteins produced by bacteriophages in the host cell, that are able to cleave one of the five bonds of the peptidoglycan cell wall, thus allowing release of progeny phage into the environment. Gram-positive endolysins generally contain two or more domains: one or more catalytic domains, often including an amidase domain and a cell wall binding domain. Tail fibre proteins are components of the phage tail, they also contain a cell binding domain (CBD) also in addition to other hydrolases that facilitate the injection of the nucleic acids into the host cell. These tail fibre hydrolases could also potentially display antimicrobial activity. In this study, the Amidase domain of the vB_LmoS_293 endolysin (225AA of the full length 339AA), the full-length tail protein (377 AA), plus a fragment of the tail protein similar to *Lactococcus* phage TP901-1 tripod base (239 AA) were produced recombinantly for the purposes of purification and characterization. The genes encoding these peptides were cloned into the expression vector, pCri8A and transformed in *E. coli*, expressed and purified with affinity chromatography. Activity against *L. monocytogenes* cells and biofilms is currently being tested and future work will lead to *in situ* experiments in a pilot scale mushroom-producing facility.

P58 Characterization of bacteriophages infecting *Salmonella enterica* serovar Heidelberg, a pathogen responsible for meat contamination

KATARZYNA KOSZNIK-KWAŚNICKA[1], **AGATA JURCZAK-KUREK**[2], **SYLWIA BLOCH**[1], **BOŻENA NEJMAN-FALEŃCZYK**[1], **ALICJA WĘGRZYN**[3], **GRZEGORZ WĘGRZYN**[1]

[1] University of Gdańsk, Department of Molecular Biology, Gdańsk, Poland

[2] University of Gdańsk, Department of Molecular Evolution, Gdańsk, Poland

[3] Polish Academy of Sciences, Institute of Biochemistry and Biophysics, Gdańsk, Poland

Corresponding author: katarzyna.kwasnicka@biol.ug.edu.pl

Salmonellosis is one of most common foodborne diseases. Every year more than 100 million people suffers from infections caused by *Salmonella* rods all over the world, and about 90 thousand cases ends with patient's death. Main sources of human infection are contaminated chicken meat and eggs. In recent years, we observe a rapid growth of bacterial resistance to antibiotics commonly used to prevent *Salmonella* outbreaks on poultry farms. Among those multiresistant pathogens, there is *Salmonella enterica subsp. enterica* serovar Heidelberg that is responsible for outbreaks in countries such as United States and United Kingdom in recent years. The aim of this work was to analyze if bacteriophages can be used to effectively eradicate *S. enterica* serovar Heidelberg. We have characterized growth parameters of bacteriophages that were isolated from environmental samples and are able to infect *S. enterica* serovar Heidelberg. We have studied phage ability to infect bacteria in different growth conditions and we have also compared efficiency of infection with a single phage with multiple phages combined into cocktail. We hope that our work will arouse an interest in phages as potential mean of prevention against Salmonella contamination of food products.

P59 Microfluidic and membrane encapsulation of bacteriophages for controlled release applications

DANISH MALIK
Loughborough University, Chemical Engineering, Chemical Engineering Department, Loughborough, United Kingdom
Corresponding author: d.j.malik@lboro.ac.uk

Objectives: The aim of the work was to evaluate the use of novel microfluidic and membrane based techniques for encapsulation of purified (using ultrafiltration and ion exchange) bacteriophages in stimuli responsive polymers (microparticles) and liposomes (microparticles and nanoparticles) for treating multi-drug resistant gastrointestinal infections.
Methods: Bacteriophage K (*S. aureus*, myovirus) and Felix O1 (*S. enterica*, myovirus) were used in the study. Phages were amplified using a Sartorius Biostat® operated in batch mode. Phages were purified using centrifugation, ultrafiltration, anion exchange chromatography and size exclusion chromatography to remove host cell proteins and DNA.
Results: We show that we can achieve significant concentration of highly purified phages (~10^{12} PFU/ml) using downstream processing operations by removing host cell proteins and host cell DNA. We have proof-of-principle results demonstrating controlled encapsulation of phages in pH responsive polymeric microparticles and liposomal micro- and nanoparticles using microfluidic and membrane encapsulation techniques. Our approach allowed precise control over phage loading per particle (i.e. control over phage dose). We show how scalable production of highly uniform micro- and nanoparticles may be achieved using membrane emulsification with control over particle size through manipulation of membrane shear and fluid properties. Through changes to the formulation and the particle size, we also demonstrate control over the release dynamics (burst and sustained release formulations) of the phages in response to a pH or enzyme trigger. We demonstrate that encapsulated phages may be stored under refrigerated conditions following drying by addition of disaccharides such as trehalose as excipients.
Conclusions: We have demonstrated that novel microfluidic and membrane based processes can be successfully used to encapsulate phages in stimuli responsive micro- and nanoparticles. These systems would allow delivery of phages to the gastrointestinal tract giving unprecedented control over the phage dose and the release profile to treat multi-drug resistant infections.

P60 Bacteriophages as anti-biofilm agents of *Flavobacterium psychrophilum*

TOM WIKLUND[1], INGER DALSGAARD[2]
[1] Åbo Akademi University, Laboratory of Aquatic Pathobiology, Turku, Finland
[2] Technical University of Denmark, National Veterinary Institute, Lyngby, Denmark
Corresponding author: twiklund@abo.fi

The bacterial fish pathogen *Flavobacterium psychrophilum* that occurs in two different colony phenotypes, rough and smooth, is the causal agent of Bacterial Cold Water Disease in fresh water salmonid farms worldwide. This phenotypic variation influences the adherence and biofilm formation of the cells on inert surfaces. The escalating problem with antibiotic resistance in bacterial fish pathogens has led to increased interest in the use of bacteriophages as treatment. However, the effect of these bacteriophages against biofilms of fish pathogens like *F. psychrophilum* has achieved relatively little attention. The aim of this study was to test two lytic (Siphoviridae) and two non-lytic (Podoviridae and Myoviridae) bacteriophages on the development of biofilms (immediately after the bacterial cell adhesion) and on mature biofilms (after biofilm formation) of smooth and rough *F. psychrophilum* isolates. Experiments were done in 96-well microplates and biofilm production was evaluated using the crystal violet staining procedure. Our results showed that both lytic phages significantly inhibited the development of biofilms of all tested isolates, while the non-lytic phages significantly decreased the biofilms. We suggest that the disruption of the development of biofilms was probably a combination of cell lysis by the lytic phages and EPS degradation by the non-lytic phages. In contrast, mature biofilms of both smooth and rough isolates were only partly affected by the bacteriophages, suggesting that *F. psychrophilum* cells in biofilms seem to be partly protected against lytic phages.

P61 Are phages part of our immunity?

ANDREJ BALÁŽ[1,2,3], JAROSLAV BUDIŠ[1,2,3], MICHAL KAJSÍK[1,4], IVETA GAZDARICOVÁ[1], ORSOLYA BÍRÓ[5], BÁLINT NAGY[6], JÁN TURŇA[1,4], TOMÁŠ SZEMES[1,3,4]

[1] Comenius University, University Science Park, Bratislava, Slovakia
[2] Comenius University, Faculty of Mathematics, Physics and Informatics, Bratislava, Slovakia
[3] Geneton Ltd, Bratislava, Slovakia
[4] Comenius University, Faculty of Natural Sciences, Bratislava, Slovakia
[5] Semmelweis University, Genetic Laboratory, 1st Dep. of Obstetrics and Gynecology, Budapest, Hungary
[6] University of Debrecen, Department of Human Genetics, Faculty of Medicine, Debrecen, Hungary
Corresponding author: andrejbalaz001@gmail.com

Introduction: Phages are the most abundant organisms on the planet. Their life cycle consists of infecting bacterial hosts and replicating inside their cells. Attempts to use phages to our advantage in fighting with antibiotic-resistant bacteria already appeared and gain on importance due to increasing reports of failing antibiotic-based therapies.

In our study, we focused on natural occurrence of phages in humans. Our preliminary data suggests presence of bacteriophage DNA in human circulation. Materials and methods: We carried out massively parallel sequencing of total DNA from plasma of healthy individuals and individuals undergoing antiviral treatment. DNA fragments which mapped to the human reference genome (version hg38) were filtered. We assigned each unmapped fragment a taxonomic label using metagenomic classifier Clark. Taxonomic composition of samples was compared and visualized using Krona graphs. We assembled filtered reads using assembler specialized for assembly of viral genomes called Savage.

Results: We identified presence of phage sequences in a small number of reads in studied samples. In addition, we observed significant drop in phage sequences in individuals undergoing antiviral treatment. In the results, we present breakdown of the identified phage data.

Conclusions: Preliminary data suggest presence of phage DNA in circulating free nucleic acids. We hypothesize that host immune system tolerates phages to assist in control and stabilization of microenvironment. More rigorous analysis needs to be carried out and supportive experiments are needed to confirm the finding as well as to study our hypotheses.

P62 Characterization of the infection dynamics, host range and genomics of six lytic bacteriophages of the foodborne pathogen *Escherichia coli* O157:H7

BEATRIZ CABRAL E PACHECO[1], KIM STANFORD[2], TIM A. MCALLISTER[3], ANASTASIA ELIAS[1], YAN D. NIU[2], DOMINIC SAUVAGEAU[1]

[1] University of Alberta, Department of Chemical and Materials Engineering, Edmonton, AB, Canada

[2] Agriculture Centre, Alberta Agriculture and Forestry, Lethbridge, AB, Canada

[3] Lethbridge Research and Development Centre, Agriculture and Agri-Food Canada, Lethbridge, AB, Canada

Corresponding author: cabralep@ualberta.ca

Despite multiple control measures in all steps of the food production chain, outbreaks of foodborne diseases continue to be a constant concern for the general public, the food industry, and public health agencies. Shiga toxin-producing *Escherichia coli* O157:H7 (STEC O157:H7) is currently one of the major foodborne pathogens in North America, associated with over 70,000 annual cases of illness in the U.S. alone. This generates significant costs from hospitalizations, product recalls, destruction of suspected products, temporary food facilities closures, and loss of consumer trust. Healthy cattle are the primary reservoir of STEC O157:H7 and, consequently, of its bacteriophages. In the present study, six bacteriophages previously isolated from cattle fecal matter and able to infect and lyse STEC O157:H7 were characterized and assessed as candidates for biocontrol and detection applications in food products before delivery to consumers. For each bacteriophage, host range and lytic capability was determined against 30 STEC O157:H7 strains. More detailed infection dynamics and virulence index were evaluated for strains of particular interest. Moreover, bacteriophages were morphologically characterized through transmission electron microscopy, and size and digestion patterns of their respective genomes were determined. This study provides a framework for the identification and selection of bacteriophages as biosanitation agents or biosensors for STEC O157:H7.

Panel Discussion – Participants

„Quo vadis, deutsche Bakteriophagenforschung?“

(in German language only)

Moderators:
Dr. Christine Rohde & PD Dr. Wolfgang Beyer

- **Dr. Isabelle Bekeredjian-Ding**, Paul-Ehrlich-Institute (PEI), Langen
- **Dr. Brigitte Brake**, Federal Institute for Drugs and Medical Devices (BfArM), Bonn
- **Dr. Anne Endmann**, VDI/VDE Innovation + Technik GmbH, Berlin
- **Prof. Dr. Andreas Kuhn**, University of Hohenheim, Stuttgart
- **Dr. Hansjörg Lehnherr**, Phage Technology Center (PTC) GmbH, Bönen
- **Dr. Wolfgang Mutter**, HYpharm GmbH, Bernried am Starnberger See
- **Dr. Christine Rohde**, Leibniz Institute DSMZ, Braunschweig

List of Participants

ARNOLD, EILEEN
Fraunhofer IGB, Stuttgart, Germany
Eileen.Arnold@igb.fraunhofer.de

ATAMER, ZEYNEP
University of Hohenheim, Stuttgart, Germany
zeynep.atamer@uni-hohenheim.de

BAILER, SUSANNE M.
Fraunhofer IGB, Stuttgart, Germany
Susanne.Bailer@igb.fraunhofer.de

BAMFORD, DENNIS
University of Helsinki, Finland
dennis.bamford@helsinki.fi

BARBIRZ, STEFANIE
University of Potsdam, Germany
barbirz@uni-potsdam.de

BEAN, JESSICA
BASF, Ludwigshafen am Rhein, Germany
jessica-eleanor.bean@basf.com

BEDNAREK, AGNIESZKA
Institute of Biochemistry and Biophysics Polish Academy of Sciences, Warsaw, Poland
a.kielan@ibb.waw.pl

BEIMS, HANNES
LAVES, Celle Germany
Hannes.Beims@LAVES.Niedersachsen.de

BEKEREDJIAN-DING, ISABELLE
Paul-Ehrlich-Institut, Langen, Germany
isabelle.bekeredjian-ding@pei.de

BELCREDI, ALEXANDER
PhagoMed, Vienna, Austria
alexander.belcredi@phagomed.com

BERNASCONI, ODETTE JOELLE
University of Bern, Switzerland
odette.bernasconi@hotmail.com

BEYER, WOLFGANG
University of Hohenheim, Stuttgart, Germany
wolfgang.beyer@uni-hohenheim.de

BÖHRINGER, MICHAEL
Friedrich-Loeffler-Institut, Jena, Germany
michael.boehringer@fli.de

BOULANGER, PASCALE
Centre national de la recherche scientifique, Gilf-sur-Yvette, France
pascale.boulanger@i2bc.paris-saclay.fr

BRACK, MARKUS
Charité – Universitätsmedizin, Berlin, Germany
Markus.Brack@charite.de

BRAKE, BRIGITTE
Federal Institute for Drugs and Medical Devices (BfArM), Bonn, Germany
brigitte.brake@bfarm.de

BREER, HEINZ
University of Hohenheim, Stuttgart, Germany
breer@uni-hohenheim.de

BRETAUDEAU, LAURENT
Clean Cells, Bouffere, France
laurent.bretaudeau@lb4biotech.com

BRÖKER, NINA
University of Potsdam, Germany
nina.broeker@uni-potsdam.de

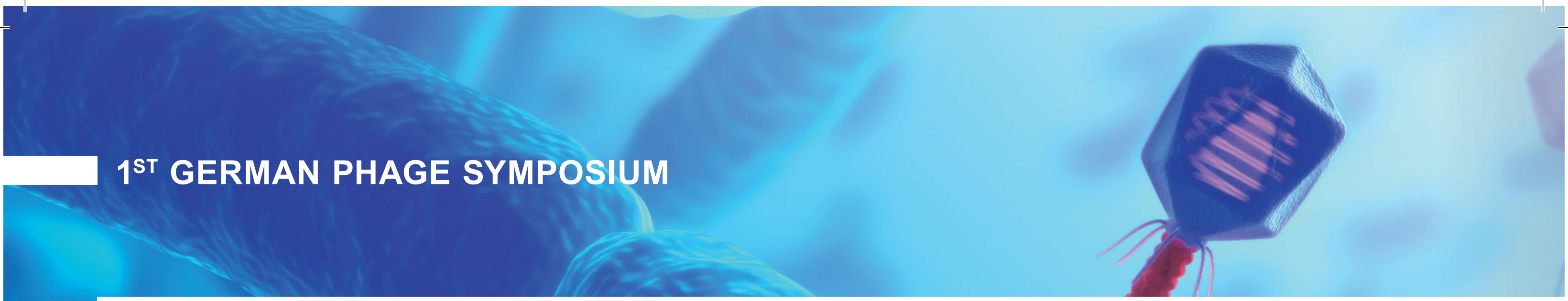

List of Participants

BROUNS, STAN
Delft University of Technology, The Netherlands
stanbrouns@gmail.com

BRÜSSOW, HARALD
Nestlé Research Center, Lausanne, Switzerland
harald.bruessow@rdls.nestle.com

BRØNSTED, LONE
University of Copenhagen, Denmark
lobr@sund.ku.dk

BUDIS, JAROSLAW
Comenius University, Bratislava, Slovakia
budis@dcs.fmph.uniba.sk

BUFE, THORSTEN
University of Hohenheim, Stuttgart, Germany
thorsten.bufe@uni-hohenheim.de

BUGERT, JOACHIM JAKOB
Bundeswehr, Institute of Microbiology Munich, Germany

CABRAL E PACHECO, BEATRIZ
University of Alberta, Edmonton, Canada
cabralep@ualberta.ca

CASTRO-MEJÍA, JOSUÉ L.
University of Copenhagen, Denmark
jcame@food.ku.dk

CHATZINOTAS, ANTONIS
Helmholtz Centre for Environmental Research – UFZ, Leipzig, Germany
antonis.chatzinotas@ufz.de

CHIBANI, CYNTHIA MARIA
University of Goettingen, Germany
cchiban@gwdg.de

COFFEY, AIDAN
Cork Institute of Technology, Cork, Ireland
aidan.coffey@cit.ie

CORSINI, LORENZO
PhagoMed, Vienna, Austria
lorenzo.corsini@phagomed.com

COX, CONSTANZE
Robert Koch Institut, Berlin, Germany
CoxC@rki.de

D'ALVISE, PAUL
University of Hohenheim, Stuttgart, Germany
paul.dalvise@uni-hohenheim.de

DENG, LI
Helmholtz Zentrum München, Neuherberg, Germany
li.deng@helmholtz-muenchen.de

DI LUCA, MARIAGRAZIA
Charité – Universitätsmedizin Berlin, Germany
mariagrazia.di-luca@charite.de

DOSKAR, JIRI
Masaryk University, Brno, Czech Republic
doskar@sci.muni.cz

DREISEIKELMANN, BRIGITTE
University of Bielefeld, Germany
b.dreiseikelmann@uni-bielefeld.de

DU, XIN
University of Tübingen, Germany
xin.du@student.uni-tuebingen.de

ENDMANN, ANNE
VDI/VDE Innovation + Technik GmbH, Berlin, Germany
anne.endmann@vdivde-it.de

List of Participants

ERAZO GARZON, ANDREA CAROLINA
University College Cork, Ireland
114224203@umail.ucc.ie

FEUERBAUM, STEFANIE
University of Hohenheim, Stuttgart, Germany
Stefanie.Feuerbaum@uni-hohenheim.de

FIESELER, LARS
Zurich University of Applied Sciences, Wädenswil, Switzerland
lars.fieseler@zhaw.ch

FINK, MICHAEL
FINK TEC GmbH, Bönen, Germany
Michael.Fink@finktec.com

FISCHER, SAMUEL
DuckTec Brüterei GmbH, Bad Belzig, Germany
samuel.fischer@wiesenhof.de

FRANZ, CHARLES
Max Rubner-Institut, Kiel, Germany
charles.franz@mri.bund.de

FRUNZKE, JULIA
Forschungszentrum Jülich, Germany
j.frunzke@fz-juelich.de

FUNK, CHRISTINA
Fraunhofer IGB, Stuttgart, Germany
Christina.Funk@igb.fraunhofer.de

GAMBINO, MICHELA
University of Copenhagen, Denmark
mgambino@sund.ku.dk

GERLACH, DAVID
University of Tübingen, Germany
d.gerlach@student.uni-tuebingen.de

GHAELI, IMA
SicoCare (under incorporation), Porto, Portugal
ema.ghaeli@gmail.com

GILLIS, ANNIKA
Université Catholique de Louvain, Louvain-la-Neuve, Belgium
annika.gillis@uclouvain.be

GLIESCHE, CHRISTIAN
University Greifswald, Germany
christian.gliesche@uni-greifswald.de

GÓRSKI, ANDRZEJ
Polish Academy of Sciences, Wroclaw, Poland
agorski@ikp.pl

GUTSAL, VALERY
Arla Foods amba, Arhus N, Denmark
valery.gutsal@arlafoods.com

HAGENS, STEVEN
Micreos BV, Wageningen, The Netherlands
s.hagens@micreos.com

HAJDO, LUKASZ
cc pharma GmbH, Densborn, Germany
l.hajdo@cc-pharma.de

HAMMERL, JENS ANDRE
German Federal Institute for Risk Assessment, Berlin, Germany
jens-andre.hammerl@bfr.bund.de

HANELT, INES
Fraunhofer IGB, Stuttgart, Germany
ines.hanelt@igb.fraunhofer.de

List of Participants

HASSELMANN, MARTIN
University of Hohenheim, Stuttgart, Germany
martin.hasselmann@uni-hohenheim.de

HEIKINHEIMO, ANNAMARI
Helsinki University, Finland
annamari.heikinheimo@helsinki.fi

HERTEL, CHRISTIAN
German Institute for Food Technology, Quakenbrück, Germany
j.niehaus@dil-ev.de

HERTEL, ROBERT
University of Göttingen, Germany
rhertel@gwdg.de

HERTWIG, STEFAN T.
German Federal Institute for Risk Assessment, Berlin, Germany
stefan.hertwig@bfr.bund.de

HINRICHS, JÖRG
University of Hohenheim, Stuttgart, Germany
J.Hinrichs@uni-hohenheim.de

HORZ, HANS-PETER
RWTH Aachen University, Germany
hhorz@ukaachen.de

JANSEN, MATHIAS
RWTH Aachen University, Germany
mjansen@ukaachen.de

JANSSEN, JOANNES
Sico Care, Porto, Portugal
joannes.janssen@chello.at

JAULT, PATRICK
Percy Military Hospital, Clamart, France
jaultpatrick@gmail.com

KACZOROWSKA, JOANNA
University College Cork, Ireland
jkaczorowska.blur@gmail.com

KALLIES, RENÉ
Helmholtz Centre for Environmental Research – UFZ, Leipzig, Germany
rene.kallies@ufz.de

KILJUNEN, SAIJA
University of Helsinki, Finland
saija.kiljunen@helsinki.fi

KITTLER, SOPHIE
University of Veterinary Medicine Hannover, Foundation, Germany
sophie.kittler@tiho-hannover.de

KLATT, ANN-BRIT
Charité – Universitätsmedizin Berlin, Germany
ann-brit.klatt@charite.de

KLEPPEN, HANS PETTER
ACD Pharma, Oslo, Norway
hans.kleppen@acspharma.com

KLUMPP, JOCHEN
ETH Zürich, Switzerland
jochen.klumpp@hest.ethz.ch

KNAPPE, DANIEL
EnBiotix GmbH, Leipzig, Germany
daniel.knappe@enbiotix.com

List of Participants

KOBERG, SABRINA
Max Rubner-Institut, Kiel, Germany
sabrina.koberg@mri.bund.de

KÖHLER, SUSANNE
Robert Koch Insitut, Berlin, Germany
koehlerS@rki.de

KOSZNIK-KWAŚNICKA, KATARZYNA
University of Gdańsk, Poland
katarztna.kwasnicka@biol.ug.edu.pl

KOZLOVA, YULIA
Russian Academy of Sciences, Siberian Branch,
Novosibirsk, Russia
ulona@ngs.ru

KRAUßE, THOMAS
Friedrich-Schiller-University Jena, Germany
thomas.krausse@uni-jena.de

KREIENBAUM, MAXIMILIAN
Friedrich-Schiller-Universität Jena, Germany
maximilian.kreienbaum@mikro.bio.uni-giessen.de

KROIČA, JUTA
Riga Stradins University, Latvia
juta.kroica@rsu.lv

KROJ, ANDREA
PTC Phage Technology Center GmbH, Bönen,
Germany
a.kroj@ptc-phage.com

KUHN, ANDREAS
University of Hohenheim, Stuttgart, Germany
andikuhn@uni-hohenheim.de

KUTATELADZE, MZIA
G. Eliava Institute of Bacteriophages,
Tbilisi, Georgia
kutateladze@pha.ge

LAMPI, MIRKA
University of Helsinki, Finland
mirka.lampi@helsinki.fi

LEDERER, PETER
Langen, Germany
drprlr@gmx.de

LEHNHERR, HANSJÖRG
PTC Phage Technology Center GmbH, Germany
h.lehnherr@ptc-phage.com

LEPRINCE, AUDREY
Université Catholique de Louvain, Belgium
audrey.leprince@student.uclouvain.be

LEPTIHN, SEBASTIAN
University of Hohenheim, Stuttgart, Germany
leptihn@uni-hohenheim.de

LI, XUEHUA
University Hospital Tübingen, Germany
xuehua.li@uni-tuebingen.de

LIDOLT, MELANIE
University of Hohenheim, Stuttgart, Germany
eidner@uni-hohenheim.de

LIESEGANG, HEIKO
University of Göttingen, Germany
hlieseg@gwdg.de

LINCETTO, CHIARA
Cambridge, UK / LMU Munich, Germany
chiaralince@hotmail.it

List of Participants

LOH, BELINDA
University of Hohenheim, Stuttgart, Germany
belinda.loh@uni-hohenheim.de

MAGALHAES, JÚLIA
LAQV/REQUIMTE, Porto, Portugal
jmagalh@fe.up.pt

MAHILLON, JACQUES
Université Catholique de Louvain, Belgium
jacques.mahillon@uclouvain.be

MALIK, DANISH
Loughborough University, UK
d.j.malik@lboro.ac.uk

MASLANOVA, IVANA
Masaryk University, Czech Republic
iva.maslanova@gmail.com

MAYER, CHRISTOPH
University of Tübingen, Germany
christoph.mayer@uni-tuebingen.de

MILLER, STEFAN
Lisando GmbH, Regensburg, Germany
stefan.miller@lisando.com

MOROZOVA, VERA
Russian Academy of Sciences, Siberian Branch, Novosibirsk, Russia
vera_morozova@ngs.ru

MUTTER, WOLFGANG
HYPharm GmbH, Bernried am Starnberger See, Germany
wolfgang.mutter@hyglos.de

NAPIERALA, RAFAL
MicroMol GmbH, Karlsruhe, Germany
info@micromol.com

NEVE, HORST
Max Rubner-Institut, Kiel, Germany
horst.neve@mri.bund.de

NIKOLICH, MIKELJON
Walter Reed Army Institute of Research, Silver Spring, USA
mikeljon.p.nikolich.civ@mail.mil

OLCHAWA, EWA
IBSS BIOMED S.A., Krakow, Poland
ewa.olchawa@biomed.pl

PAGAVA, KARAMAN
Tbilisi State Medical University, Georgia
kpagava@yahoo.com

PAJUNEN, MARIA I.
University of Helsinki, Finland
maria.pajunen@helsinki.fi

PANTUCEK, ROMAN
Masaryk University, Czech Republic
pantucek@sci.muni.cz

PENNONE, VINCENZO
Teagasc, Fermoy, Ireland
vincenzo.pennone@teagasc.ie

RABSCH, WOLFGANG
Robert Koch Institut, Wernigerode, Germany
RabschW@rki.de

RACENIS, KARLIS
Riga Stradins University, Latvia
karlis.racenis@rsu.lv

List of Participants

RENFERT, KEVIN
University of Leipzig, Germany
kevin.renfert@vogelklinik.uni-leipzig.de

ROHDE, CHRISTINE
Leibniz Institute DSMZ, Braunschweig, Germany
chr@dsmz.de

ROHMER, CARINA
University Hospital Tübingen, Germany
carina.rohmer@med.uni-tuebingen.de

ROMERO-SAAVEDRA, LUIS FELIPE
LMU Munich, Germany
frsquim@gmail.com

ROSE, THOMAS
Queen Astrid Military Hospital Brussels, Belgium
thomasrose@telenet.be

ROSSIER, OMBELINE
CNRS, Paris-Sud, France
ombeline.rossier@u-psud.fr

RUBALSKII, EVGENII
Hannover Medical School (MHH), Germany
Rubalskii.Evgenii@mh-hannover.de

SABIROVA, JULIA
Leibniz Institute DSMZ, Braunschweig, Germany
jus16@dsmz.de

SAIER, REGINE
University of Hohenheim, Stuttgart, Germany
eidner@uni-hohenheim.de

SAILE, NADJA
University of Hohenheim, Stuttgart, Germany
nadja.saile@uni-hohenheim.de

SAMTLEBE, MEIKE
University of Hohenheim, Stuttgart, Germany
Meike.Samtlebe@uni-hohenheim.de

SANGWIMAN, ANUCHA
Mahidol University, Bangkok, Thailand
sensmith.anucha@gmail.com

SCHMIDT, HERBERT
University of Hohenheim, Stuttgart, Germany
herbert.schmidt@uni-hohenheim.de

SCHMIDT, IMKE
Leibniz Institute DSMZ, Braunschweig, Germany
ims16@dsmz.de

SCHULZE, RENATE
B.R.A.I.N. AG, Zwingenberg, Germany
rs@brain-biotech.de

SHARP, NATASHA
Guild BioSciences , Charleston, USA
nsharp@GuildBioSciences.com

SIMON, KEVIN
RWTH Aachen University, Germany
kevin.simon@rwth-aachen.de

SKURNIK, MIKAEL
University of Helsinki, Finland
mikael.skurnik@helsinki.fi

STEGLICH, CLAUDIA
University of Freiburg, Germany
claudia.steglich@biologie.uni-freiburg.de

STUIVER, MAARTEN
BASF Plant Science Company GmbH,
Limburgerhof, Germany
maarten.stuiver@basf.com

List of Participants

SULAKVELIDZE, ALEXANDER
Intralytix Inc., Baltimore, USA
asulakvelidze@intralytix.com

SZEWZYK, REGINE
German Environment Agency, Berlin, Germany
regine.szewzyk@uba.de

SZEWZYK, ULRICH
Technische Universität Berlin, Germany
ulrich.szewzyk@tu-berlin.de

TAVARES, PAULO
I2BC, Gilf-sur-Yvette, France
paulo.tavares@i2bc.paris-saclay.fr

THORMANN, KAI
University of Gießen, Germany
kai.thormann@mikro.bio.uni-giessen.de

TIKUNOVA, NINA
Russian Academy of Sciences, Siberian Branch, Novosibirsk, Russia
tikunova@niboch.nsc.ru

TKHILAISHVILI, TAMTA
Charité – Universitätsmedizin Berlin, Germany
tamta.tkhilaishvili@charite.de

TROJOK, RÜDIGER
UnternehmerTUM gGmbH, Garching, Germany
trojok@unternehmertum.de

VAN DER LUIT, ARNOLD
Clean Cells, Bouffere, France
avandurluit@clean-cells.com

VAN MECHELEN, ELS
University College Gent, Belgium
Els.VanMechelen@hogent.be

VERMEULEN, STEFAN
University College Gent, Belgium
Els.VanMechelen@hogent.be

VOGEL, JÖRG
University of Würzburg, Germany
monika.meece@uni-wuerzburg.de

VOGENSEN, FINN K.
University of Copenhagen, Denmark
fkv@food.ku.dk

WAGNER, NATALIA
Max Rubner-Institut, Kiel, Germany
natalia.wagner@mri.bund.de

WAHL, ASTRID
CNRS, Marseille, France
awahl@imm.cnrs.fr

WEBER, LUCAS
University of München, Germany
lucas_weber@gmx.de

WEIDENBACH, KATRIN
University of Kiel, Germany
kweidenbach@ifam.uni-kiel.de

WEILAND-BRÄUER, NANCY
University of Kiel, Germany
nweiland@ifam.uni-kiel.de

WEIß, AGNES
University of Hohenheim, Stuttgart, Germany
agnes.weiss@uni-hohenheim.de

WENDLING, CAROLIN
GEOMAR Helmholtz Centre for Ocean Research Kiel, Germany
cwendling@geomar.de

List of Participants

WIENOLD, SANDRA-MARIA
Charité – Universitätsmedizin Berlin, Germany
sandra.wienhold@charite.de

WIKLUND, TOM
Åbo Akademi University, Turku, Finland
twiklund@abo.fi

WILMES, MIRIAM
Federal Institute for Drugs and Medical Devices (BfArM), Bonn, Germany
miriam.wilmes@bfarm.de

WITTMANN, JOHANNES
Leibniz Institute DSMZ, Braunschweig, Germany
jow12@dsmz.de

WITZENRATH, MARTIN
Charité – Universitätsmedizin Berlin, Germany
martin.witzenrath@charite.de

WOLZ, CHRISTIANE
University of Tübingen, Germany
christiane.wolz@med.uni-tuebingen.de

ZDAREK, DRAHOSLAV
CC Pharma GmbH, Densborn, Germany
d.zdarek@cc-pharma.de

Authors & Co-Authors Index

A

Aleshkin, Andrey P41
Alibegashvili, Maia P50
Alkhnbashi, Omer S. P4
Almeida, Cláudio P49
Andrade Martínez, Juan S. P8
Andres, Yves P46
Atamer, Zeynep P56
Ayora, Silvia O4

B

Babkin, Igor P5, P33
Baláž, Andrej P61
Bamford, Dennis K1, P54, Chair
Barbirz, Stefanie O6, P3
Baron, Maayan P19
Bashkina, Olga P41
Bauersachs, Thorsten P4
Bednarek, Agnieszka P55
Beims, Hannes O23
Bekeredjian-Ding, Isabelle Panel Disc.
Beyer, Wolfgang P16, Chair
Bierbrodt, Anna P45
Billaudeau, Cyrille O4
Binnenkade, Lucas P18
Bíró, Orsolya P61
Bloch, Sylwia P58
Bochkareva, Svetlana P41
Bokovaya, Olga P5
Born, Yannick K4, P48
Botka, Tibor P36
Bottacini, Francesca P25
Boulanger, Pascale O3, P9
Boyle, Erin Colleen P42
Brack, Markus P39
Brinks, Erik P27, P52
Broecker, Felix P38
Bröker, Nina P3
Brouns, Stan J.J. K2, Chair
Brüssow, Harald O26, Chair
Brønsted, Lone P2, P26
Budiš, Jaroslav P61
Bylov Hedegaard Andersen, J. P26

C

Cabral e Pacheco, Beatriz P62
Cambillau, Christian P25
Carballido-Lopez, Rut O4
Castro-Mejía, Josué L O13
Chakhunashvili, Natela P50
Chatzinotas, Antonis P19, P22
Chibani, Cynthia Maria P6
Chkhartishvili, Ekaterine O18
Coffey, Aidan O24, P57

D

Dąbrowski, Kamil P55
Dalsgaard, Inger P60
de Leeuw, Marina P19
Deng, Li O9
Denyes, Jenna K4
De Vos, Daniel O17
Di Luca, Mariagrazia P24, P31, P40
Dietert, Kristina P39
Doskar, Jiri P14, P15, P36
Du, Xin P29
Duffy, Brion P48
Dunne, Matthew K4

E

Elias, Anastasia P62
Eliava, Tatiana P50
Endmann, Anne Panel Disc.
Engelsen, Søren B O13
Erazo Garzon, Andrea Carolina P25
Ergin, Firuze P56
Eskelin, Katri P54

Ferreira, Vânia P49
Fieseler, Lars K4, P48
Filipek, Agnieszka P55
Fink, Michael P45
Fischer, Samuel O22
Franz, Charles O24, P27, P52
Fortuna, Wojciech O14
Franz, Charles O25, P27, P52
Frunzke, Julia O7

G

Gabisonia, Taras P50
Galimyzanov, Khalil P41
Gambino, Michela P26
Ganichev, Dennis P34
Garrec, Nathalie P46
Gawor, Jan P55
Gazdaricová, Iveta P61
Gencay, Y. Emre P2, P26
Ghaeli, Ima P35
Gholadze, Sopo O18
Gillis, Annika O2, P7
Głowacka, Aleksandra P55
Glünder, Gerhard O22
Golijashvili, Rati O18
Gonçalves, Pilar P49
Górski, Andrzej O14, P55
Gozdek, Agnieszka P55
Griebler, Christian P20
Gromadka, Robert P55
Gruber, Achim D P39
Guerrero-Ferreira, Ricardo K4

H

Haase, Max O5, P10
Hagens, Steven O27
Hammerl, Jens Andre P11
Han, Jeonghee P44
Hansen, Lars H O13
Happonen, Lotta J. P23
Harms, Hauke P19
Hartke, Axel P13
Hasselmann, Martin Chair
Haverich, Axel P41, P42
Heikinheimo, Annamari P28
Heinemann, Udo P3
Heller, Knut J. P25
Hendrickx, Antoni P.A. P13
Hertel, Robert P6
Hertwig, Stefan O20, P11
Hinrichs, Jörg O25, P56, Chair
Hock, Louise P7
Holm, Lars O13
Horsma-Heikkinen, Jenni P30
Horz, Hans-Peter O11
Hübner, Johannes P13
Hünnefeld, Max O7
Hupfeld, Mario K4

I

Indrakova, Adela P15
Izak, Dariusz P55

J

Jacobsen, Rasmus O13
Jacquet, Eric O4
Jaeckel, Claudia P11
Jakutyte, Lina O4
Janssen, Joannes P35
Japarashvili, Nana O18
Jault, Patrick O16
Jennes, Serge O17
Johnke, Julia P19
Jordan, Kieran P57
Jun, Jin Woo P23

J

Jung, Kirsten P13
Jurczak-Kurek, Agata P58
Jurkevitch, Edouard P19

K

Kalandarishvili, Tamar P50
Kallies, René P20, P22
Kajsík, Michal P61
Karpiskova, Renáta P36
Katamadze, Taimuraz P50
Kiesel, Bärbel P22
Kiljunen, Saija P30
Kim, Sungjae P44
Kim, Youngihn P44
Kiseleva, Irina P41
Kittler, Sophie O22, P45
Klatt, Ann-Brit P31, P40
Klumpp, Jochen K4, Chair
Koberg, Sabrina P27
Kolackova, Ivana P36
Kopf, Matthias P12
Kosznik-Kwaśnicka, Katarzyna P58
Kot, Witold O13
Kothe, Erika P21
Kozlova, Yulia P5, P33, P34, P43
Krauße, Thomas P21
Kreienbaum, Maximilian P18
Krobsrisate, Sunee P51
Kroiča, Juta P53
Krych, Łukasz O13
Kücückcetin, Ahmet P56
Kuhn, Andreas O5, P10, Chair
Kühn, Christian P41, P42
Kühner, Petra P29
Kupczok, Anne P4
Kushmaro, Ariel P19
Kutateladze, Mzia K3, Chair
Kuusela, Pentti P30

L

Labarde, Audrey O4
Lampi, Mirka P54
Lehnherr, Hansjörg P45, Panel Disc.
Lehnherr, Tatiana P45
Leiman, Petr K4
Leptihn, Sebastian O5, P10, Chair
Leskinen, Katarzyna P30
Letkiewicz, Sławomir O14
Letzel, Stefanie P32
Liesegang, Heiko O12, P6
Lindell, Debbie P12
Łobocka, Małgorzata P55
Loessner, Martin K4, P48
Loh, Belinda O5, P10
Loladze, Manana P50

M

Magalhaes, Júlia P49
Magin, Vanessa P46
Mahillon, Jacques O2, P7, Chair
Mahony, Jennifer P25, P37
Malik, Danish P59
Marti, Roger K4
Martin, Karin P21
Mashaqi, Bakr P41, P42
Maslanova, Ivana P14, P15, P36
Matīsa, Dace P53
McAllister, Tim A. P62
McAuliffe, Olivia P57
Mengden, Ruth P45
Meyer, Mareike P3
Miedzybrodzki, Roman O14
Moelling, Karin P38
Morozova, Vera P5, P33, P34, P43
Muangsombut, Veerachat P51
Mueller, Lukas O5
Mutter, Wolfgang O19, Panel Disc.

N

Nadiradze, Manana P50
Nagy, Bálint P61
Nejman-Faleńczyk, Bożena P58
Neve, Horst O21, O25, P4, P25, P27, P52, Chair
Nielsen, Dennis S O13
Niu, Yan D. P62
Noeckler, Karsten P11
Nouailles, Geraldine P39

O

Oksanen, Hanna P54

P

Pagava, Karaman O18
Pajunen, Maria I. P23
Pantucek, Roman P14, P15, P36
Pees, Michael P47
Pekarski, Irena P12
Pennone, Vincenzo P57
Peschel, Andreas P29
Pētersons, Aivars P53
Pfeifer, Eugen O7
Phothaworn, Preeda P51
Pirnay, Jean-Paul O17
Ponien, Prishila O4
Poranen, Minna P54

R

Rabsch, Wolfgang P47
Racenis, Karlis P53
Reetz, Jochen P11
Reitelseder, Søren O13
Renouard, Madalena P9
Reyes, Alejandro P8
Rogóż, Paweł O14
Rohde, Christine O28, P32, P39, P45, Panel Disc, Chair.
Rohde, Manfred P32, P39, P45
Rohmer, Carina P16, P17
Romero-Saavedra, Luis Felipe P13
Rose, Thomas O17
Roske, Yvette P3
Ross, Anton P39
Rossier, Ombeline P9
Rossmann, Friederike P13
Różycka, Aleksandra P55
Rubalskii, Evgenii P41, P42
Rubalsky, Oleg P41
Rümke, Stefan P41, P42
Ruzickova, Vladislava P36

S

Sabirova, Julia P32
Sadowy, Ewa P55
Salmoukas, Christina P41, P42
Samtlebe, Meike O25, P56
Sangwiman, Anucha P51
Sanz Gaitero, Marta P57
Sauvageau, Dominic P62
Schmidt, Herbert O10, Chair
Schmidt, Imke P45
Schmitz, Ruth A. P4
Scholz, Holger P11
Seitz, Claudius P39
Shed‘ko, Elizaveta P33
Skurnik, Mikael P23, P30
Stanford, Kim P62
Stazic, Damir P12
Steglich, Claudia P12
Stierhof, York-Dieter P29
Stribna, Sabina P14

Suttorp, Norbert P39
Sørensen, Martine Holst P2
Szemes, Tomáš P61

T

Tamarashvili, Natia P50
Tavares, Paulo O4
Teixeira, Paula P49
Tholey, Andreas P4
Thormann, Kai P18
Tikunov, Artem P43
Tikunova, Nina P5, P33, P34, P43
Tkhilaishvili, Tamta P24, P31
Trampuz, Andrej P24, P31, P40
Tuomala, Henni P30
Turňa, Ján P61

U

Ushakova, Tatiana P33

V

van Raaij, Mark Johan P57
van Sinderen, Douwe P25, P37
Verbeken, Gilbert O17
Vlassov, Valentin P34
Vogensen, Finn K. O13, P25
Voit, Annekatrin P21

W

Wagner, Natalia O25, P52
Wahl, Astrid P1
Weber-Dąbrowska, Beata O14, P55
Węgrzyn, Alicja P58
Węgrzyn, Grzegorz P58
Weidenbach, Katrin P4
Wicklund, Anu P30
Wienhold, Sandra-Maria P39
Wiklund, Tom P60
Winstel, Volker P29
Wittmann, Johannes O1, P32, P45
Witzenrath, Martin O15, P39, Chair
Wolz, Christiane O8, P17

Y

Yunusova, Anastasiya P5

Z

Zangelmi, Leo P9
Zeman, Michal P15
Ziehr, Holger P39
Zulkarneev, Eldar P41

Notes

Notes

Notes

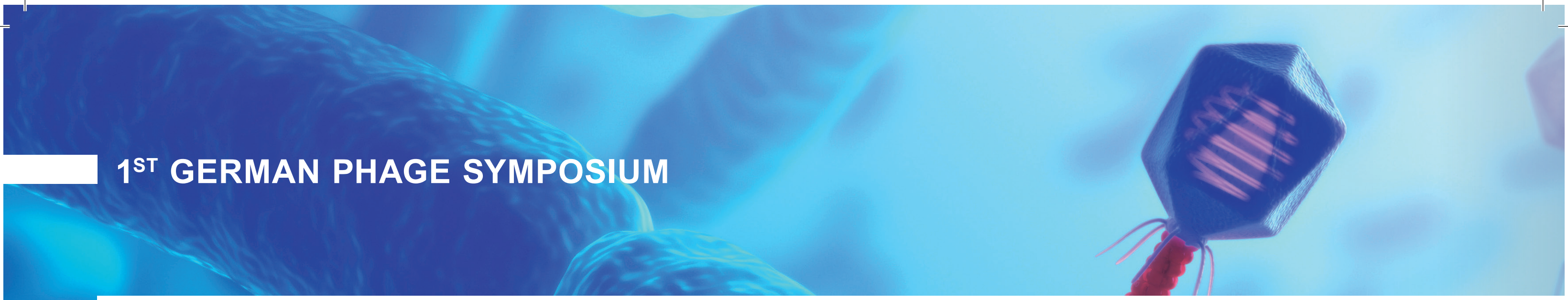

Notes

Notes

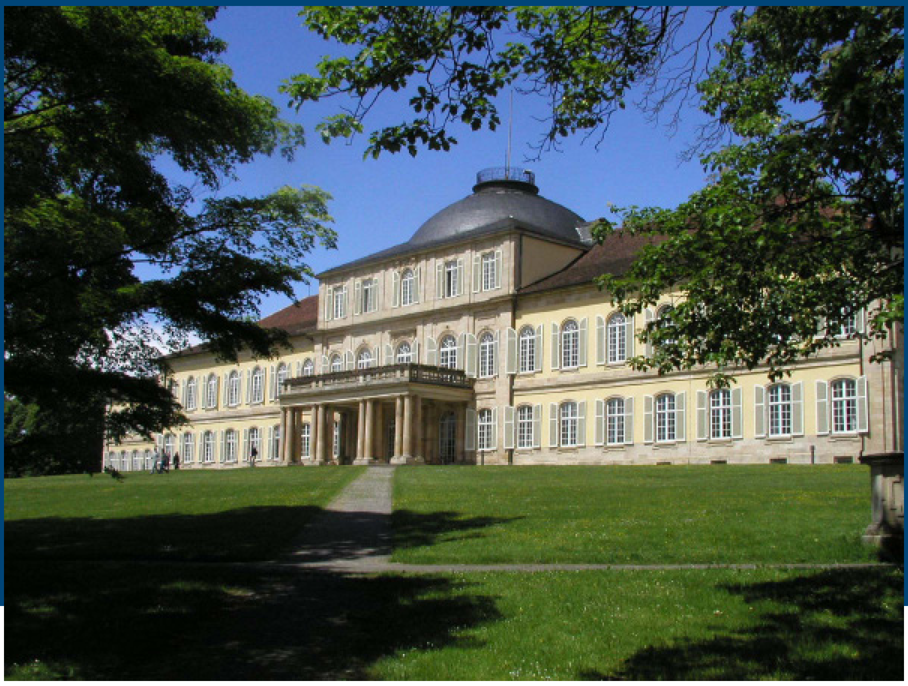

Imprint

Editor: University of Hohenheim, Research Center for Heath Sciences
Wollgrasweg 43, 70599 Stuttgart, +49 (0)711 459 24615, fzg@uni-hohenheim.de
https://health.uni-hohenheim.de
Printed at the University of Hohenheim, KIM, September 2017
Layout: unger+ kreative strategen GmbH, Esperantostraße 12, 70197 Stuttgart
Cover photo: ktsdesign – Fotolia (bacteriophage attack)
